# Low expression of ONECUT2 governs the POU6F2-beta-catenin axis to modulate cancer stemness and drives the CCL28-dependent pathway for macrophage polarization in breast cancer

**DOI:** 10.1038/s41419-026-08909-5

**Published:** 2026-06-02

**Authors:** Meng Shen, Haixia Jin, Ziqi Huang, Nan Dong, Gen Liu, Yu Zeng, Yuan Meng, Yumeng Liu, Lili Yang, Xiubao Ren

**Affiliations:** 1 Tianjin Medical University Cancer Institute and Hospital, National Clinical Research Center for Cancer, Tianjin, China; 2 Key Laboratory of Cancer Prevention and Therapy, Tianjin, China; 3 Tianjin’s Clinical Research Center for Cancer, Tianjin, China; 4 Key Laboratory of Cancer Immunology and Biotherapy, Tianjin, China; 5 Department of Biotherapy, Tianjin Medical University Cancer Institute and Hospital, Tianjin, China; 6 Department of Immunology, Tianjin Medical University Cancer Institute and Hospital, Tianjin, China; 7 Haihe Laboratory of Cell Ecosystem, Tianjin, China; 8 Department of Hematology, Oncology Center, Tianjin Union Medical Center, The First Affiliated Hospital of Nankai University, Tianjin, China

**Keywords:** Breast cancer, Cancer microenvironment

## Abstract

Cancer stem cells (CSC) and the immunosuppressive microenvironments are key drivers of breast cancer (BC) progression and drug resistance. However, the molecular mechanisms by which One Cut Homeobox 2 (ONECUT2) governs CSC and the tumor immune microenvironment (TIME) remain largely unknown. Given the critical knowledge gap, we sought to investigate ONECUT2’s regulatory impact on CSC properties and TIME profiles using BC cell lines, animal models, and clinical specimens. Here, we demonstrated that inhibition of ONECUT2, a core transcription factor, potently drives CSC characteristics and reprograms the TIME to favor macrophage polarization to the M2-type, a tumor-promoting state. Mechanistically, ONECUT2 inhibition transcriptionally activated POU6F2, which subsequently triggered beta-catenin, thereby enhancing CSC properties and chemoresistance in BC. With respect to modulating the immune microenvironment, suppression of ONECUT2 governs macrophage polarization toward the immunosuppressive M2 phenotype. CCL28 is identified as a transcriptional target of ONECUT2 required for M2-type macrophage polarization, and CCR10 as a key receptor involved in this immune modulation. These findings highlight the critical involvement of ONECUT2 in modulating BC stemness via targeting the POU6F2-beta-catenin axis and managing macrophage polarization to M2 phenotype through the CCL28-CCR10 pathway. Our study suggests that ONECUT2 modulates cancer stemness and the TIME, and that targeting its downstream POU6F2-beta-catenin axis and CCL28-CCR10 pathway may provide an effective approach for BC treatment.

## Introduction

Breast cancer (BC) is the predominant malignant tumor among women and has a profound impact on their health [1]. Cancer stem cells (CSC) exhibit intrinsic resistance to chemotherapeutic agents, which contributes to tumor progression and recurrence in BC. Exploring how chemotherapy regulates BC stemness is of great significance for reversing chemoresistance and ultimately improving patient prognosis. In our previous study [2], we identified a novel mechanism by which chemotherapy induces BC cells to secrete extracellular vesicles (EVs) that serve as critical mediators of therapeutic resistance. These EVs targeted the transcription factor One Cut Homeobox 2 (ONECUT2), thereby inducing a CSC-like phenotype and chemoresistance in BC. ONECUT2, a core member of the ONECUT family, is a transcription factor that is involved in multidirectional differentiation [3]. ONECUT2 contributes to various oncogenic processes, including tumor proliferation, metastasis, epithelial-mesenchymal transition (EMT), angiogenic signaling, endocrine therapy resistance, and subtype switching in BC [4–6]. However, the effects of ONECUT2 on CSC properties are complex, stemming from its interactions with multiple signaling pathways and molecular mechanisms that collectively reshape CSC phenotypes. As a transcription factor, ONECUT2 modulates CSC traits by regulating the downstream genes PPP2R4 [7] or TFPI [8] in gastric cancer (GC). Another study [4] elucidates the roles of ONECUT2 in stemness maintenance, verifying beta-catenin as a crucial downstream effector. In non-small cell lung cancer (NSCLC), ONECUT2 has emerged as a vital downstream effector in CSC driven by lncRNA THUMPD3-AS1 [9], highlighting its potential as a therapeutic target in NSCLC. Although our findings suggest a regulatory role of ONECUT2 in CSC properties, the underlying molecular mechanisms are poorly understood. This knowledge gap underscores the need to fully elucidate the mechanism by which ONECUT2 mediates functional plasticity in CSC, thereby defining a clear priority for future investigation. In our study, we found that ONECUT2 transcriptionally targets POU6F2 (POU domain, class 6, transcription factor 2), thereby triggering beta-catenin and modulating cancer stemness and chemoresistance in BC cell lines, animal models, and clinical specimens.

Emerging evidence indicates that the interplay between CSC and the tumor immune microenvironment (TIME) drives tumor progression and immune evasion [10–13]. Specifically, CSC can foster an immunosuppressive tumor microenvironment by recruiting and modulating suppressive immune cells [14–16]. Notably, components of the TIME, such as tumor-associated macrophages (TAMs) are educated by CSC and, in turn, create a niche that supports CSC survival and stemness through paracrine and autocrine signaling [17]. Elucidating the crosstalk between CSC and the suppressive TIME offers novel perspectives for designing effective strategies to overcome therapy resistance. In NSCLC, ONECUT2 serves as a master mediator of the exhausted CD8^+^T cell phenotype [18]; thus, it has been identified as a critical regulator of cytotoxic immune responses and a novel therapeutic target for checkpoint inhibition. Another study identified ONECUT2 as a direct target of T-bet [19], establishing a central role in the differentiation of T-helper (Th) progenitor cells to Th1/Th2 effector cells. However, the precise role of ONECUT2 in the TIME of BC remains unknown. Here, through in vivo and in vitro experiments, we observed that ONECUT2 functions as a key regulator of M2-type macrophage polarization, thereby reprogramming the TIME. This study further elucidates the regulatory network of the immunosuppressive microenvironment and screens for potential immunomodulatory factors to affect immunotherapy response in BC.

## Materials and methods

### Cells and constructs

The human BC cell lines (MDA-231, MCF-7 and BT474), mouse BC cell line (4T1) and human THP-1 cell line were purchased from the American Type Culture Collection (Manassas, VA, USA), and cultured in DMEM (for MCF-7) or RPMI-1640 (for MDA-231, BT474, 4T1, and THP-1) base medium supplemented with 10% fetal bovine serum (FBS). All cancer cell lines have been authenticated by STR genotyping and have been proven to be free of mycoplasma contamination. MDA-231, MCF-7 and BT474 cells with stable ONECUT2 overexpression (MDA-231/OC-2, MCF-7/OC-2, BT474/OC-2) were generated by ONECUT2 overexpression plasmid (OriGene; Rockville, MD), and subsequently selected by G418 solution. 4T1 cells with stable ONECUT2 overexpression (4T1/OC-2) or POU6F2 knockdown (4T1/shPOU6F2) were generated by transduction with lentivirus carrying pLVX-Onecut2 (mouse, NM_194268.3) or a POU6F2 shRNA plasmid (Tsingke Biotechnology, Beijing, China), followed by puromycin selection. The siRNAs targeting ONECUT2, POU6F2, and CTNNB1, along with a negative control siRNA, were purchased from Qiagen (Venlo, Netherlands). RNA or DNA was transfected using Lipofectamine RNAiMAX (Invitrogen; Carlsbad, CA) or Lipofectamine 3000 (Invitrogen; Carlsbad, CA) following the manufacturer’s protocols.

### Patients and tissue samples

Tissue microarrays (TMAs) comprising 99 BC samples coupled with patients’ survival data were acquired from Shanghai Outdo Biotech (Shanghai, China). All experimental procedures were conducted following approval by the Ethics Committee of Tianjin Medical University Cancer Institute and Hospital (E2021075) and conducted in accordance with the Declaration of Helsinki. Informed consent was obtained from all subjects. The clinical parameters of the patients were shown in Table S1.

### Data collection and prognostic analysis

Clinical data were retrieved from the GEO datasets (https://www.ncbi.nlm.nih.gov/geo/) GSE9893, GSE3494 (platform GPL96), GSE20685 and GSE242541. Prognostic evaluation was conducted through univariate Cox proportional hazards regression and Kaplan–Meier (KM) survival analysis. KM curves were generated using the R packages “survival” and “survminer”. High and low expression groups were defined using the optimal cutoff determined by “surv_cutpoint”. Statistical comparison of survival distributions across groups was performed using log-rank tests to determine significant differences. Differential gene expression analysis was performed using Sangerbox (http://www.sangerbox.com). TCGA-BRCA data were obtained from the UCSC Xena (https://xena.ucsc.edu), and clinical subtype information for Breast Invasive Carcinoma (TCGA, PanCancer Atlas) was downloaded from the cBioPortal database (https://www.cbioportal.org). The R packages “limma”, “ggpubr”, and “RColorBrewer” were used for statistical analysis.

### Real-time quantitative PCR and western blotting

RNA extraction by TRIZOL (Invitrogen; Carlsbad, CA) and real-time quantitative PCR (RT-qPCR) were performed as previously described [2], with β-ACTIN as a control (primer sequences in Table S2). Proteins were extracted using RIPA lysis buffer, with antibodies listed in Table S2. Nuclear proteins were extracted using the Nuclear Protein Extraction Kit (Bestbio; Shanghai, China) following the manufacturer’s protocol.

### Sphere formation assay

Treated MDA-231, MCF-7 and BT474 cells were harvested and re-suspended in serum-free medium. The single-cell suspension was diluted to a density of 2000 cells per well and seeded into ultralow attachment six-well plates. The number of spheres was counted on day 21 (for MDA-231), day 14 (for MCF-7), and day 15 (for BT474), and the sphere-forming efficiency was calculated using a diameter ≥70μm as the criterion.

### Enzyme-linked immunosorbent assay

Cultured supernatants were collected and assayed for enzyme-linked immunosorbent assay (ELISA) by ElaBoXTMHuman MEC (CCL28) ELISA Kit (Solarbio; Beijing, China), Human IL-10 ELISA Kit, Human Arginase-1 (ARG1) ELISA Kit, Human/Rat/Monkey/Porcine/Bovine Transforming Growth Factor Beta 1 ELISA Kit(TGFb1) (ABclonal; Wuhan, China) following the manufacturer’s instructions.

### Flow cytometry

Single-cell suspensions were prepared from tumor tissues/spleens of mice, or from treated BC cells and THP-1 cells, followed by staining with antibodies (listed in Table S2) for flow cytometry (FCM) analyses. All samples were analyzed using a FACS with three replicates, and at least 10,000 events were acquired for each sample.

### Dual-luciferase reporter assay

MCF-7 cells were seeded at ~60% confluence in 24-well plates. For promoter reporters, 0.5 μg pGL3-Basic luciferase reporters were transfected into MCF-7 cells with ONECUT2 or POU6F2 overexpression. Empty pCMV6-Entry or pcDNA3.1(+) vector were used as control groups. The luciferase activities were determined using the Dual-Luciferase Reporter Assay System (Promega; Madison, WI, USA), as described previously [2]. Primer sequences are listed in Table S2.

### ChIP-PCR

MCF-7 cells were harvested and processed using the ChIP Assay kit (Cell Signaling Technology, Massachusetts, USA) under the manufacturer’s instructions. We utilized JASPAR (https://jaspar.elixir.no/), an open-access database of curated transcription factor binding profiles, to identify putative binding sites. The DNA enrichment was assessed by real-time PCR using the specific primers (detailed in Table S2).

### Co-immunoprecipitation

The co-immunoprecipitation (Co-IP) assay was performed using the Epizyme Co-IP Kit (YJ201; Epizyme Biotech, Shanghai, China) according to the manufacturer’s instructions. In brief, lysates of 1 × 10^7^ MDA-231 cells were incubated with anti-ONECUT2 antibody or IgG isotype control overnight at 4 °C, subsequently co-incubated with protein A/G magnetic beads for 6 h, and further eluted for western blotting. Table S2 displays the used antibodies.

### Cell viability and IC50 calculation

Cell viability was assessed using Cell Counting Kit-8 (CCK8; MedChemExpress, New Jersey, USA). Briefly, MDA-231, MCF-7 and BT474 cells were seeded at 2500 cells/well using flat-bottomed 96-well culture plates and treated with doses of docetaxel (DTX; Beyotime Biotechnology, Jiangsu, China) or doxorubicin (DOXO; Sigma-Aldrich, St. Louis, MO, USA) in a range from 0.001 μM to 100 μM. After the cells were cultured for 48 h, 10 μL CCK8 solution was added to each well, followed by 1 h incubation at 37 °C, and absorbance at 450 nm was measured. The calculation of the half maximal inhibitory concentration (IC50) was performed using GraphPad Prism version 10.4.2 (GraphPad Software, San Diego, CA, USA).

### Immunohistochemistry (IHC) and multiplex IHC staining (mIHC)

Formaldehyde-fixed, paraffin-embedded mouse tumor tissues were processed for IHC as previously described [20]. The antibodies were listed in Table S2. The whole stained slides were evaluated according to two criteria: 1) staining intensity (−: 0, +: 1, ++: 2, +++: 3) and 2) percentage of staining-positive tumor cells (0%: 0, 1–29%: 1, 30–69%: 2, ≥70%: 3). The intensity and percentage scores were multiplied to generate a final score, which were used in statistical analyses. mIHC staining was conducted by PANO 7-plex IHC Kit (Panovue, Beijing, China). DAPI was used for nuclear localization. The antibodies and their dilution ratios were listed in Table S2.

### Cell co-culture assay

THP-1 cells were treated with 5 pg/mL PMA (SinoBiological, Beijing, China) to stimulate the transformation of macrophages. After 48 h of stimulation, THP-1-derived macrophages were pre-incubated with the conditioned medium (CM) of MDA-231, MCF-7, and BT474 cells for 48 h. THP-1 cells were harvested for further analyses or were refreshed with new medium for 48 h to collect the supernatant.

### Transwell assay

For the migration assay, treated BC cells (1.5 × 10^4^ cells/well in 24-well plates) were seeded into the upper chambers of Transwell inserts (8-μm pore size, Corning, USA) and incubated for 24 h. Cells that migrated to the lower surface were fixed, then stained with crystal violet, and counted manually under a microscope. All experiments were performed in triplicate.

### Proliferation assay

MDA-231, MCF-7 and BT474 cells were seeded in 96-well plates at an initial number of 2500 cells/well. Tumor cells were co-cultured for 24 h, 48 h, and 72 h with CM collected from THP-1 cells that had been exposed to ONECUT2-downregulated, ONECUT2-upregulated, or control BC cells. Cell viability was measured by the CCK-8 assay.

### Apoptosis assay

MDA-231, MCF-7, and BT474 cells were cultured for 48 h in CM collected from THP-1 cells that had been treated with corresponding BC cells harboring ONECUT2 knockdown or overexpression. Annexin V-FITC/PI double staining was performed using the Apoptosis Detection Kit (Yeasen Biotechnology, Shanghai, China) according to the manufacturer’s instructions and analyzed by FCM.

### Animals

All animal experiments were approved by the Institutional Animal Care and Use Committee of Tianjin Medical University Cancer Institute and Hospital (E2021128). Six-week-old female NOD/SCID/IL2Rγ-null (NSG) mice and BALB/c mice were obtained from the Weitonglihua Corporation (Beijing, China). Animals were randomly assigned to experimental groups within each strain. MDA-231 (TNBC), MCF-7 (Luminal A), BT474 (HER2-positive), and 4T1 (murine TNBC), along with their stable ONECUT2-overexpressing cells (MDA-231/OC-2, MCF-7/OC-2, BT474/OC-2, 4T1/OC-2) and POU6F2-knockdown cells (4T1/shPOU6F2), were cultured as previously described.

Orthotopic tumors were established by injecting BC cells into the fourth mammary fat pad. For the Fixed Endpoint Model, BALB/c mice (*n* = 4 per group) were randomly assigned to receive an injection of 4T1, 4T1/OC-2 cells, or 4T1/shPOU6F2 cells in a blinded manner. Tumors were harvested 20 days after injection and collected for subsequent experiments. For the Chemotherapy Response Model, mice were randomized into pre-treatment and post-treatment cohort upon reaching designated volumes. The pre-treatment cohort was sacrificed immediately for baseline analyses, and the post-treatment cohort received weekly intraperitoneal (IP) injections of DTX (15 mg/kg) or DOXO (2 mg/kg) for three weeks. All mice were sacrificed three days post-final treatment, and tumors were collected for analyses. Comprehensive details of the in vivo experiments are provided in the Supplementary Materials and Methods.

### Statistical analysis

GraphPad Prism version 10.4.2 was used for graph drawing and statistical analysis. The protein expression data were classified as “high” or “low” based on the optimal cutoff determined by maximizing Youden’s index on the ROC. The results were derived from at least three independent experiments. Statistical significance between groups was assessed using Student’s unpaired *t* test, Student’s paired *t* test, Mann–Whitney test, one-way analysis of variance for multiple comparisons, log-rank test, over-representation analysis, and the Cox proportional hazards regression models, as appropriate. Pearson correlation was used to assess linear relationships in normal distribution, and Chi-Square test or Fisher’s exact test (for small sample sizes) were used to assess relationships among factorial variables. Statistical significance was set at *p* < 0.05. Multiple testing correction was performed using the two-stage step-up procedure of Benjamini, Krieger, and Yekutieli to control the false discovery rate (FDR), with significantly enriched terms defined as those with an FDR-adjusted *p* < 0.05.

## Results

### Low expression of ONECUT2 regulates BC stemness via the beta-catenin-dependent pathway

**Fig. 1:**
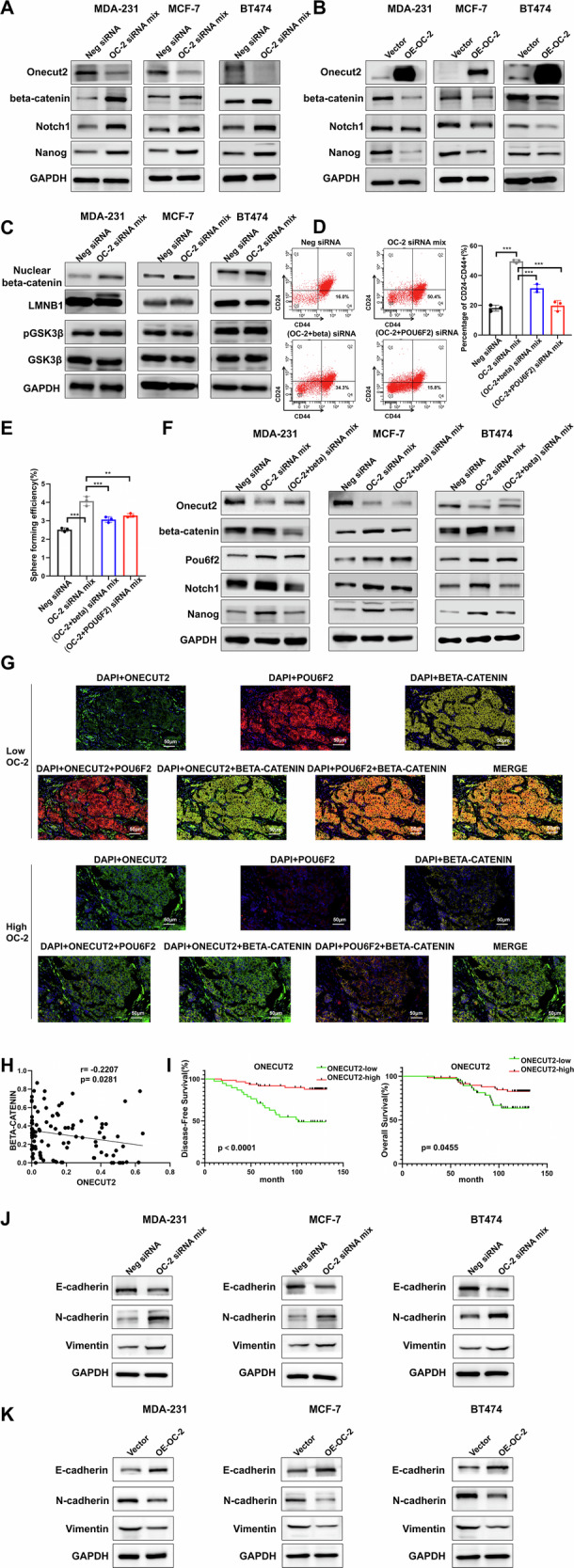
Low expression of ONECUT2 regulates breast cancer stemness via the beta-catenin-dependent pathway. **A**, **B** The expression levels of beta-catenin and stemness genes Notch1 and Nanog were verified after transfection with siONECUT2 (**A**) and an overexpressing plasmid (**B**) in MDA-231, MCF-7 and BT474 cells using western blotting. **C** Nuclear beta-catenin, pGSK3β and GSK3β expressions were detected in BC cells following ONECUT2 knockdown by western blotting analysis. LMNB1 served as a nuclear marker. **D**, **E** MCF-7 cells were transfected with a control siRNA, siONECUT2, or mixed siRNAs (ONECUT2 plus beta-catenin or ONECUT2 plus POU6F2), and collected for FCM to analyze the CD24^−^CD44^+^ cell population (**D**) and for sphere formation assay (**E**). **F** Western blots presenting the expression levels of indicated proteins in BC cells following transfection with specific siRNAs. **G** Representative mIHC staining images of ONECUT2, POU6F2 and BETA-CATENIN expression in patients with BC (DAPI: blue; ONECUT2: green; POU6F2: red; BETA-CATENIN: yellow). **H** Scatter plot showing the correlation between ONECUT2 and BETA-CATENIN (*n* = 99; *r* = −0.2207, *p* = 0.0281). **I** KM survival curves comparing clinical outcomes (*n* = 99; DFS: *p* < 0.0001, OS: *p* = 0.0455) of BC patients between high (red) and low (green) ONECUT2 expression groups. **J**, **K** Western blotting analysis of EMT markers (E-cadherin, N-cadherin, and Vimentin) in BC cells following ONECUT2 knockdown (**J**) or overexpression (**K**). Error bars represent mean ± SD of three independent experiments. *P < 0.05, **P < 0.01, ***P < 0.001. Scale bar: 50 μm.

Our previous study showed that ONECUT2 serves as a crucial determinant of CSC [2]; however, the underlying molecular mechanism remains poorly understood. Since the beta-catenin signaling pathway acts as a core set, with its target genes commonly serving as well-known stem-cell markers [21], we examined whether ONECUT2 could affect beta-catenin expression and clarify the regulatory mechanism. In MDA-231, MCF-7, and BT474 cell lines, ONECUT2 inhibition activated beta-catenin and upregulated stemness genes Notch1 and Nanog (Fig. 1A). Conversely, ONECUT2 overexpression exhibited the opposite effect (Fig. 1B). We further observed a significant accumulation in nuclear beta-catenin rather than pGSK3β in BC cells with ONECUT2 knockdown (Fig. 1C), indicating that inhibition of ONECUT2 activates the transcriptional activity of beta-catenin. However, ONECUT2 did not directly bind to the promoter of CTNNB1 (encoding beta-catenin), as evidenced by a luciferase assay showing that ONECUT2 had no effect on the CTNNB1 promoter (Fig. S1). Beta-catenin attenuated the effect of ONECUT2 on the CD24^−^CD44^+^ population known to be enriched in CSC (Figs. 1D and S2B, C), sphere-forming efficiency (Figs. 1E and S2A, S2D, S2E), and the expression of stemness gene Notch1 and Nanog (Fig. 1F) in MCF-7, MDA-231 and BT474 cells. mIHC staining confirmed a significant inverse correlation (*r* = −0.2207, *p* = 0.0281) between the expression of ONECUT2 and beta-catenin in BC patients (Fig. 1G, H). Disease-free survival (DFS) and overall survival (OS) were significantly shorter in the ONECUT2-low expression cohort (DFS, *p* < 0.0001; OS, *p* = 0.0455; Fig. 1I, Table 1), a finding corroborated by GEO dataset (GSE9893) in BC patients (Fig. S3A). Notably, reduced ONECUT2 expression was significantly associated with shorter DFS across multiple breast cancer subtypes (TNBC, Luminal A, TNBC + HER2-positive, and Luminal A + B; Fig. S3B). In our cohort, ONECUT2 showed non-significantly reduced expression in TNBC compared to favorable subtypes (*n* = 99, Fig. S3C), a result validated in the TCGA cohort with significantly lower expression in aggressive TNBC versus HER2-positive tumors (Fig. S3D). The potential involvement of ONECUT2 in regulating EMT was further evaluated. Downregulation of ONECUT2 activated the EMT program, as reflected by enhanced migratory capacity (Fig. S4A) and corresponding changes in epithelial and mesenchymal markers (Fig. 1J); conversely, its overexpression exerted the opposite effect (Fig. S4B, Fig. 1K). The effect of ONECUT2 on EMT-related traits was partially counteracted by beta-catenin in MDA-231, MCF-7, and BT474 cells (Fig. S4C, D). Collectively, these findings suggest that ONECUT2 regulate CSC and EMT via a beta-catenin-dependent pathway, and that its inhibition results in upregulated beta-catenin expression, thereby amplifying the stemness-promoting and pro-metastatic signals.

**Table 1:** Univariate survival analysis of clinical parameters and three gene expressions with DFS and OS in breast cancer patients.

				DFS		OS	
				Hazard ratio		Hazard ratio	
Clinical parameters			*n*	(95%CI)	*p* value	(95%CI)	*p* value
Age(years)							
≤50			35	1.395	**0.4311**	3.124	0.0286*
＞50			64	(0.6325 ~ 3.076)		(1.355 ~ 7.204)	
Clinical stage							
I + IIA			57	2.478	0.0196*	2.329	0.0412*
IIB ~ IIIC			42	(1.128 ~ 5.445)		(1.012 ~ 5.364)	
T classification							
T1			36	0.7215	**0.4084**	1.042	**0.9259**
T2 + T3			63	(0.3216 ~ 1.619)		(0.4439 ~ 2.444)	
N classification							
N0			54	2.18	0.0471*	1.957	**0.1092**
N1 + N2 + N3			45	(1.001 ~ 4.747)		(0.8593 ~ 4.455)	
ER							
Negative			41	0.6578	**0.282**	0.3561	0.0136*
Positive			58	(0.2998 ~ 1.443)		(0.1547 ~ 0.8201)	
PR							
Negative			54	0.8454	**0.6719**	0.3109	0.0144*
Positive			45	(0.3912 ~ 1.827)		(0.1371 ~ 0.7049)	
HER2							
Negative			88	1.167	**0.8007**	0.9301	**0.9219**
Positive			11	(0.3251 ~ 4.192)		(0.2275 ~ 3.802)	
ONECUT2							
Low			38	0.1868	<0.0001***	0.4423	0.0455*
High			61	(0.08281 ~ 0.4214)		(0.1893 ~ 1.034)	
POU6F2							
Low			59	4.106	0.0003***	3.374	0.0031**
High			40	(1.838 ~ 9.172)		(1.438 ~ 7.917)	
Beta-catenin							
Low			53	2.952	0.0074**	1.371	**0.4479**
High			46	(1.359 ~ 6.413)		(0.6020 ~ 3.120)	
Clinical subtype							
Luminal A + B			51	1.611	**0.23**	2.253	**0.06**
TNBC + HER2+			48	(0.7447 ~ 3.484)		(0.9915 ~ 5.120)	

*ER* estrogen receptor, *PR* progesterone receptor, *HER2* human epidermal growth factor receptor 2, *TNBC* triple-negative breast cancer,

*DFS* disease-free survival, *OS* overall survival *p < 0.05, **p < 0.01, and ***p < 0.001.

### ONECUT2 suppresses the transcriptional activity of POU6F2

**Fig. 2:**
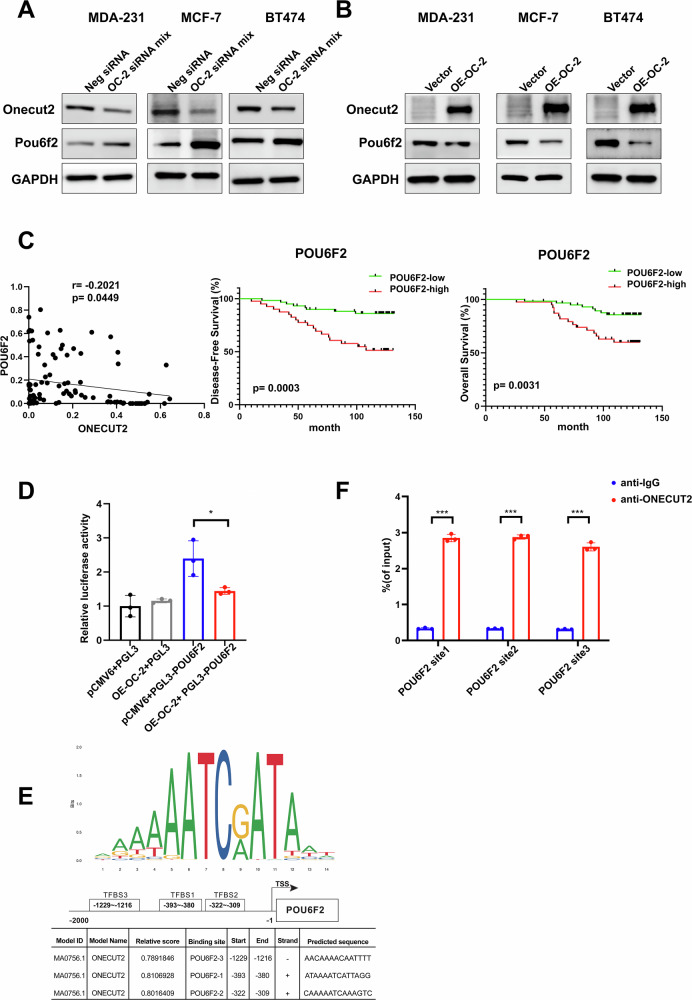
ONECUT2 suppresses the transcriptional activity of POU6F2. **A**, **B** MDA-231, MCF-7 and BT474 cells were transfected with ONECUT2 siRNAs (**A**) or its overexpression plasmid (**B**) for 48 h, protein levels of Onecut2 and Pou6f2 were detected by western blotting. **C** The correlation analysis of ONECUT2 and POU6F2 expression in BC patients (Left panel). KM curves showing DFS and OS stratified by POU6F2 expression (*n* = 99; DFS: *p* = 0.0003, OS: *p* = 0.0031; right panel; high: red; low: green). **D** Relative luciferase activities were determined after co-transfection of MCF-7 cells with a luciferase reporter plasmid containing POU6F2 promoter sequences and ONECUT2 overexpression plasmid. **E** ONECUT2-scanned motif logo. Predicted binding sites in the human POU6F2 promoters. Genomics position relative to the transcription start sites of the POU6F2, sequence, and corresponding scores. **F** Binding of ONECUT2 to the POU6F2 promoters in MCF-7 cells was determined by ChIP-PCR. Error bars represent mean ± SD of three independent experiments. *P < 0.05, **P < 0.01, ***P < 0.001.

The potential target genes of ONECUT2 were predicted using the Gene Transcription Regulation Database (http://gtrd.biouml.org/). When setting the transcription factor-binding site at the promoter [−2600, +100], we observed that POU6F2 was a promising target gene (Table S3). Given that POU6F2, reported to be regulated by ONECUT2 in embryonic retinas [3], was upregulated in ONECUT2-knockdown BC cells in our previous study [2], we conducted experiments to observe whether POU6F2 serves as a direct transcriptional target of ONECUT2, thereby mediating the regulatory effects of ONECUT2 on beta-catenin. In BC cells, ONECUT2 knockdown promoted POU6F2 expression (Fig. 2A), whereas its overexpression reduced POU6F2 expression (Fig. 2B). mIHC staining showed that ONECUT2 and POU6F2 exhibited a significant negative correlation (*r* = −0.2021, *p* = 0.0449). DFS (*p* = 0.0003) and OS (*p* = 0.0031) were significantly prolonged in patients with low POU6F2 levels (Fig. 2C, Table 1), a finding further supported by the GEO dataset (GSE3494; Fig. S5A). Our cohort analysis revealed that high POU6F2 expression significantly predicted poorer DFS and OS in aggressive subtypes (TNBC and TNBC + HER2-positive) (Fig. S5B), and was not significantly elevated in TNBC compared to HER2-positive and Luminal A subtypes (*n* = 99; Fig. S5C). The TCGA data further confirmed the significantly higher levels of POU6F2 expression in TNBC versus Luminal A. (Fig. S5D). Further, we employed a Co-IP assay to confirm the binding among ONECUT2, POU6F2, and BETA-CATENIN (Fig. S6). Dual-luciferase reporter assays (Fig. 2D) and ChIP-PCR analysis (Fig. 2E, F) demonstrated that ONECUT2 inhibits POU6F2 transcriptional activity and directly binds to its promoter region. These results provide conclusive evidence that POU6F2 is a direct transcriptional target of ONECUT2.

### POU6F2 promotes breast cancer stemness via activating beta-catenin

**Fig. 3:**
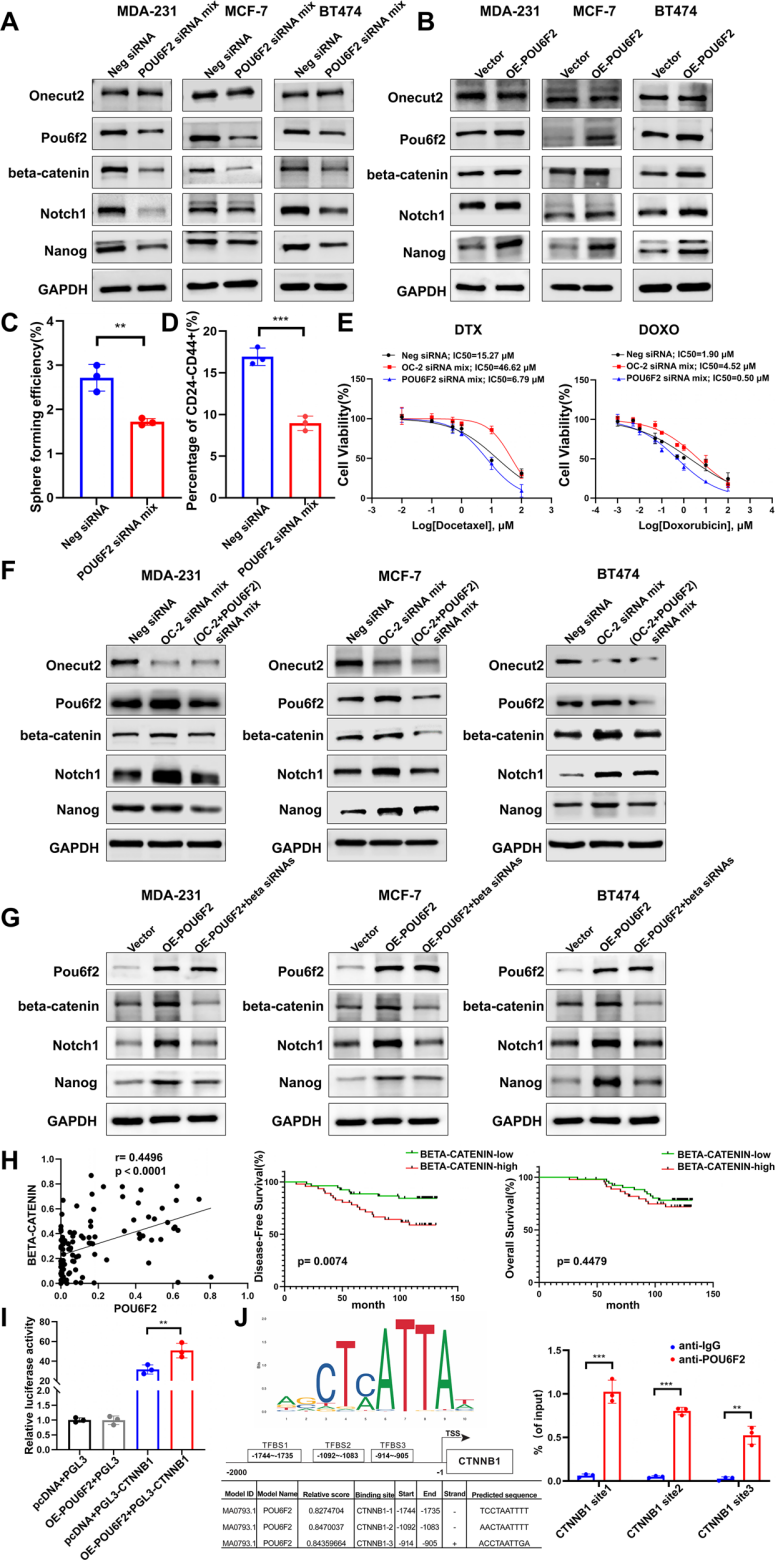
POU6F2 promotes breast cancer stemness via activating beta-catenin. **A**, **B** Western blotting was used to assess the protein levels of indicated genes after the BC cells were transfected with POU6F2 siRNAs (**A**) or an overexpression plasmid (**B**). **C** Sphere-forming efficiency was evaluated in MCF-7 cells following transfection with siPOU6F2 or a control siRNA. **D** The proportion of CD24^−^CD44^+^ cells in MCF-7 cells, with or without POU6F2 knockdown was determined by FCM analysis using anti-CD24-PE and anti-CD44-PerCP-Cy5.5 conjugated antibodies. **E** MDA-231 cells were transfected with siRNA targeting POU6F2 or ONECUT2, followed by 48 h treatment with DTX (Left panel, 0.01 μM to 100 μM) or DOXO (Right panel, 0.001 μM to 100 μM). Cell viability was measured by CCK-8 assay, with IC50 values calculated via nonlinear regression. **F** The indicated protein levels were detected in BC cells using western blotting. **G** Western blots presenting the expression levels of indicated proteins in BC cells after transfection with POU6F2-overexpressing plasmid alone or in combination with beta-catenin siRNAs. **H** The correlation analysis of POU6F2 and BETA-CATENIN expression in BC patients was performed by mIHC (Left panel). KM curves showed DFS and OS in patients stratified by beta-catenin expression levels (*n* = 99; DFS: *p* = 0.0074, OS: *p* = 0.4479; right panel, high: red, low: green). **I** Dual-luciferase reporter assays were used to observe the transcriptional regulation of CTNNB1 by POU6F2. **J** POU6F2 motif logo and predicted binding sites in the CTNNB1 promoter (Left panel). ChIP-PCR confirmation of POU6F2 binding to the CTNNB1 promoter in MCF-7 cells (Right panel). Error bars represent mean ± SD of three independent experiments. *P < 0.05, **P < 0.01, ***P < 0.001.

Based on these findings, we speculated that POU6F2 may play a core role in exerting ONECUT2-mediated suppression of beta-catenin activity and CSC characteristics in BC. POU6F2 deficiency in MDA-231, MCF-7, and BT474 cells resulted in decreased protein levels of beta-catenin and stemness-associated genes, including Notch1 and Nanog (Fig. 3A). Conversely, POU6F2 overexpression markedly increased the expression of these proteins (Fig. 3B), suggesting a regulatory function of POU6F2 on beta-catenin and the maintenance of CSC. Furthermore, the sphere formation ability and the CD24^−^CD44^+^ cell population decreased with POU6F2 knockdown in MCF-7 cells (Figs. 3C, D and S7A, B), MDA-231 cells (Fig. S7C, D) and BT474 cells (Fig. S7E, F). Inhibition of POU6F2 significantly reduced resistance to DTX and DOXO in MDA-231, MCF-7 and BT474 cells; while ONECUT2 knockdown increased resistance to both drugs in these cell lines (Figs. 3E and S8). However, direct chemotherapy failed to show a significant or consistent effect on the expression of ONECUT2 or its targets (Fig. S9). Furthermore, we observed a notable reduction of beta-catenin and stemness genes (Fig. 3F), the CD24^−^CD44^+^ cell population (Figs. 1D and S2B, C), and the sphere formation ability (Figs. 1E and S2A, D, E) in the ONECUT2-deficient group with POU6F2 knockdown. In line with this, the POU6F2-induced CSC characteristics were partially rescued by beta-catenin (Figs. 3G and S10). As shown in Fig. 3H, POU6F2 demonstrated a strong positive correlation with beta-catenin (*r* = 0.4496, *p* < 0.0001). Furthermore, patients with elevated beta-catenin expression exhibited significantly shortened DFS (*p* = 0.0074), while no statistical association was observed between beta-catenin levels and OS (*p* = 0.4479, Table 1), a pattern corroborated by GEO data (GSE20685, Fig. S11A). Elevated BETA-CATENIN expression predicted diminished DFS in HER2-positive and combined TNBC + HER2-positive cases (*n* = 99, Fig. S11B). Additionally, while BETA-CATENIN was non-significantly elevated in TNBC in our cohort (Fig. S11C), the elevated expression was confirmed as significant in the TCGA datasets (Fig. S11D).

Mechanistically, using dual-luciferase reporter assays, we found that POU6F2 served as a transcriptional activator of CTNNB1, revealing significantly increased promoter activity upon POU6F2 overexpression (Fig. 3I). ChIP-PCR analysis further confirmed that POU6F2 binds to the promoter region of CTNNB1 (Fig. 3J). Taken together, these data highlight POU6F2 as a crucial mediator in activating beta-catenin, thereby promoting cancer stemness and chemoresistance in BC.

### The low ONECUT2-POU6F2-beta-catenin axis enhances breast cancer stemness and chemoresistance in vivo

**Fig. 4:**
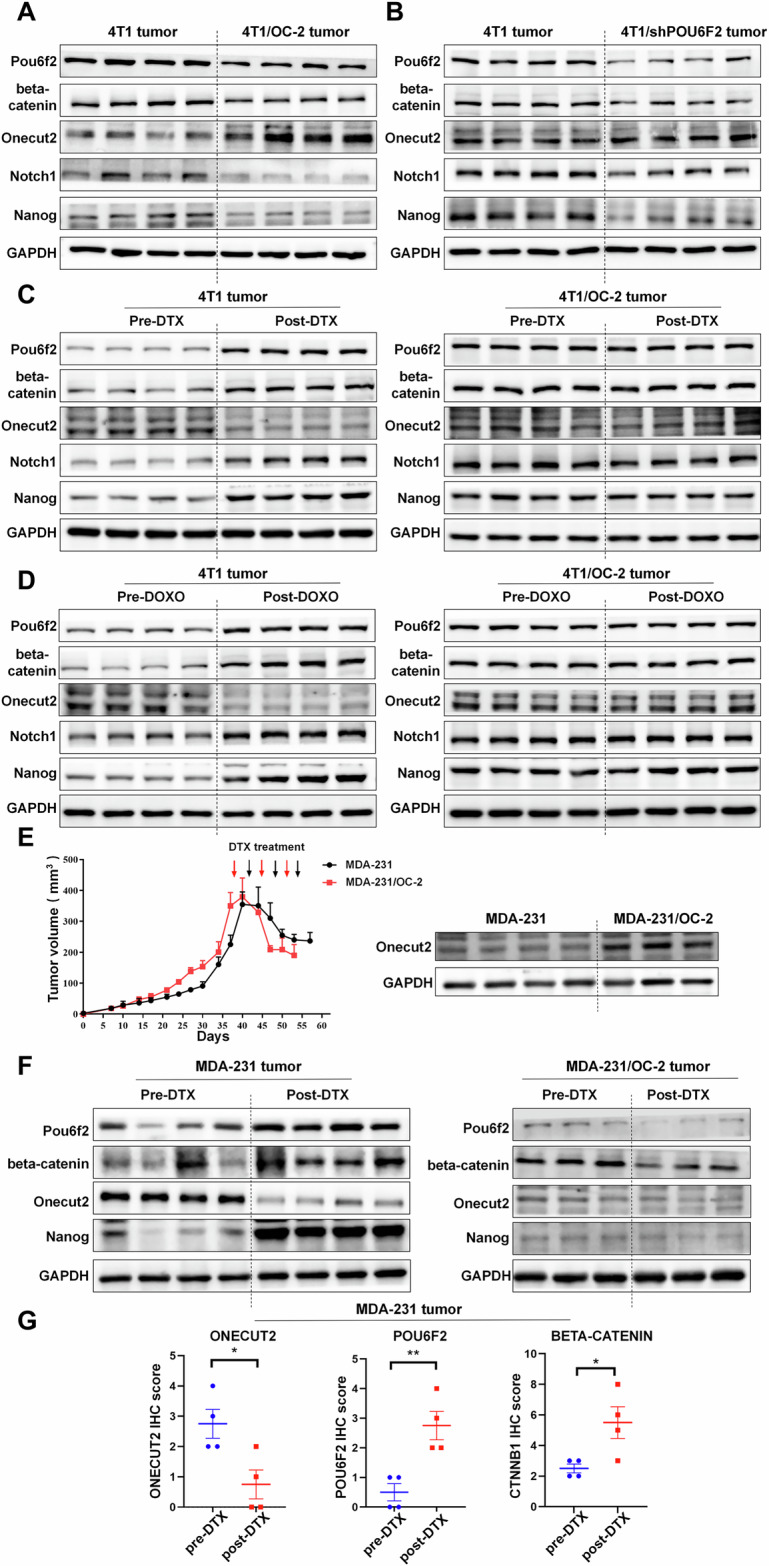
The low ONECUT2-POU6F2-beta-catenin axis enhances breast cancer stemness and chemoresistance in vivo. **A** Tumor tissues were collected from BALB/c mice bearing 4T1 (*n* = 4) and 4T1/OC-2 (*n* = 4) tumors for western blotting analysis. **B** Tumor tissues were collected from BALB/c mice bearing 4T1 (*n* = 4) and 4T1/shPOU6F2 (*n* = 4) tumors for western blotting analysis. **C** Western blots of indicated proteins in tumor tissues from 4T1 mice (*n* = 4; Left panel) and 4T1/OC-2 mice (*n* = 4; Right panel) collected before and after DTX treatment. **D** Western blots of indicated proteins in tumor tissues from 4T1 mice (*n* = 4; Left panel) and 4T1/OC-2 mice (*n* = 4; Right panel) collected before and after DOXO treatment. **E** Xenograft tumors generated in NSG mice by injecting MDA-231 (*n* = 4) and MDA-231/OC-2 (*n* = 3) cells. Tumor volume was measured, with DTX treatments marked by arrows (Left panel, red arrows for MDA-231/OC-2 tumors and black arrows for MDA-231 tumors, indicating treatment with DTX). The protein level of ONECUT2 in NSG tumor models was measured (Right panel). **F** Western blots of indicated proteins in tumor tissues from MDA-231 mice (*n* = 4; Left panel) and MDA-231/OC-2 mice (*n* = 3; Right panel) collected before and after DTX treatment. **G** The expression levels of Onecut2, Pou6f2 and beta-catenin in the MDA-231 mice tissues pre-DTX and post-DTX were detected by IHC staining. Error bars represent mean ± SD of three independent experiments. *P < 0.05, **P < 0.01.

To further evaluate the role of the ONECUT2-POU6F2-beta-catenin axis in modulating BC stemness and chemotherapy resistance, we constructed BALB/c mouse models bearing tumors derived from 4T1, 4T1/OC-2 cells, and 4T1/shPOU6F2 cells (Fig. S12). A significant decrease of Pou6f2 and beta-catenin expression was observed in 4T1/OC-2 tumors relative to 4T1 tumors (Fig. 4A). In mice bearing 4T1/shPOU6F2 tumors, we observed decreased expression of beta-catenin and stemness-related genes compared to those bearing 4T1 tumors (Fig. 4B). In chemotherapy-treated 4T1 tumor models (Fig. S13A), both DTX (Fig. 4C Left panel) and DOXO (Fig. 4D Left panel) induced a significant reduction in Onecut2 expression, paralleled by elevated levels of Pou6f2, beta-catenin, and stemness genes Notch1 and Nanog. In contrast, these treatments exerted no measurable impact on 4T1/OC-2 tumors (Fig. 4C Right panel, 4D Right panel). Consistent findings emerged in MDA-231 NSG mice following DTX/DOXO treatment (Figs. 4E–G, S13B and S14), as well as in MCF-7 (Fig. S15) and BT474 (Fig. S16) NSG mouse models following DTX treatment. Furthermore, tumors with ONECUT2 overexpression and POU6F2 knockdown exhibited better response to DTX and DOXO treatment, as evidenced by enhanced tumor regression (Figs. S13A, S14A and S15A Left panel, S16A Left panel, Fig. 4E Left panel, Fig. S17). These results further support the critical role of the low ONECUT2-POU6F2-beta-catenin axis in maintaining cancer stemness and chemoresistance in BC.

### ONECUT2 suppression drives macrophage polarization to M2 phenotype in the breast cancer microenvironment

**Fig. 5:**
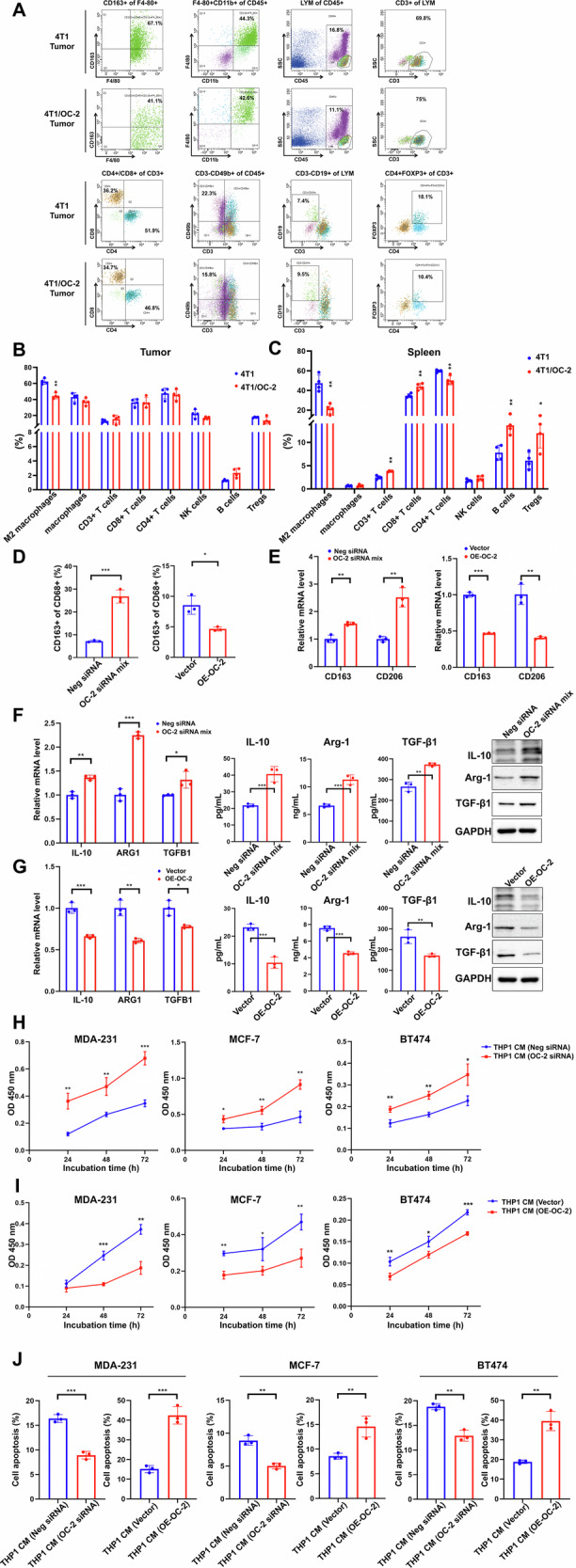
ONECUT2 suppression drives macrophage polarization to M2 phenotype in the breast cancer microenvironment. **A**, **B** FCM was used to assess dynamic changes in immunocyte subsets (M2 macrophages (CD163+ of F4-80 + ; FDR-adjusted *p* = 0.0026), macrophages (F4-80 + CD11b+ of CD45 + ; FDR-adjusted *p* = 0.2317), CD3^+^ T cells (CD3+ of CD45 + ; FDR-adjusted *p* = 0.4414), CD4^+^T cells (CD4+ of CD3 + ; FDR-adjusted *p* = 0.7269), CD8^+^T cells (CD8+ of CD3 + ; FDR-adjusted *p* = 0.8751), B cells (CD3-CD19+ of CD45 + ; FDR-adjusted *p* = 0.0542), NK cells (CD3-CD49b+ of CD45 + ; FDR-adjusted *p* = 0.2110) and Tregs (CD4+Foxp3+ of CD3 + ; FDR-adjusted *p* = 0.2110) within the tumor tissues of 4T1/OC2 (*n* = 4) and 4T1 (*n* = 4) mice. **C** FCM was used to assess dynamic changes in immunocyte subsets (M2 macrophages (CD163+ of F4-80 + ; FDR-adjusted *p* = 0.0036), macrophages (F4-80 + CD11b+ of CD45 + ; FDR-adjusted *p* = 0.4239), CD3^+^T cells (CD3+ of CD45 + ; FDR-adjusted *p* = 0.0017), CD4^+^T cells (CD4+ of CD3 + ; FDR-adjusted *p* = 0.0052), CD8^+^T cells (CD8+ of CD3 + ; FDR-adjusted *p* = 0.0039), B cells (CD3-CD19+ of CD45 + ; FDR-adjusted *p* = 0.0039), NK cells (CD3-CD49b+ of CD45 + ; FDR-adjusted *p* = 0.0846) and Tregs (CD4+Foxp3+ of CD3 + ; FDR-adjusted *p* = 0.0117) within the spleens of 4T1/OC2 (*n* = 4) and 4T1 (*n* = 4) mice. **D** THP-1 cells were co-cultured with MDA-231 conditioned medium, and the CD163^+^CD68^+^ population was assessed by FCM analysis. **E** RT-qPCR analysis of CD163 and CD206 mRNA levels in THP-1 cells treated with conditioned medium from MDA-231 cells with ONECUT2 inhibition (Left panel) or overexpression (Right panel), compared to respective control groups. **F**, **G** RT-qPCR (Left panel), ELISA (Middle panel), and western blots (right panel) analyses of IL-10, Arg-1, and TGF-β1 expression in THP-1-derived macrophages following co-culture with conditioned medium from MDA-231 cells under ONECUT2 inhibition (**F**) or overexpression (**G**). **H**, **I** MDA-231 (left panel), MCF-7 (middle panel) and BT474 cells (right panel) were co-cultured with CM from THP-1 cells that had been primed with ONECUT2-downregulated (**H**) or ONECUT2-overexpressing (**I**) breast cancer cells. Tumor cell proliferation at 24 h, 48 h, and 72 h was detected using the CCK-8 assay. **J** FCM analysis of cell apoptosis in breast cancer cells co-cultured with CM from THP-1 cells that had been primed with ONECUT2-downregulated (Left panel) or ONECUT2-overexpressing (Right panel) breast cancer cells. Error bars represent mean ± SD of three independent experiments, *P < 0.05, **P < 0.01, ***P < 0.001.

Growing evidence suggests that there are complex interactions between CSC and TIME [22, 23]. To determine the regulatory roles of ONECUT2 in the TIME, FCM was used to assess dynamic changes in immunocyte subsets within the tumor tissues and spleens of 4T1/OC-2 and 4T1 mice. Statistical analysis with FDR correction revealed a significant decrease in M2 macrophage infiltration in 4T1/OC-2 tumors (Fig. 5A, B; FDR-adjusted *p* = 0.0026, *n* = 4) relative to 4T1 tumors (*n* = 4). The differences for all other immune cell subsets were not statistically significant (FDR-adjusted *p* > 0.05). Spleen tissues from 4T1/OC-2 mice exhibited significant reductions in M2 macrophages and CD4^+^T cell infiltration, accompanied by elevated percentages of CD3^+^T cells, CD8^+^T cells, B cells, and regulatory T cells (Tregs) (Figs. 5C and S18; FDR-adjusted *p* < 0.05, *n* = 4). Consistent results were also observed for THP-1-derived macrophages. Co-culture of THP-1 cells with CM from ONECUT2-knockdown MDA-231 cells significantly increased the CD163^+^ M2 macrophage population (Fig. 5D Left panel, S19A Left panel) and upregulated the expression of the pro-tumorigenic M2 markers CD163 and CD206 (Fig. 5E, Left panel). Conversely, ONECUT2 overexpression demonstrated opposite effects (Fig. 5D right panel, S19A right panel, and 5E right panel). Macrophages pre-incubated with CM from MCF-7 (Fig. S19B, D) and BT474 cells (Fig. S19C, E) demonstrated consistent effects. Meanwhile, the expression levels of IL-10, Arg-1 and TGF-β1 were upregulated in macrophages co-cultured with CM from ONECUT2-knockdown MDA-231 (Fig. 5F, G), MCF-7 (Figs. S20A, C, E, F), or BT474 (Fig. S20B, D–F) cells. To further examine how ONECUT2-mediated macrophage polarization affects BC cell functions, we measured proliferation, apoptosis, and migration of BC cells after exposure to CM from THP-1 macrophages pre-incubated with ONECUT2-modified BC cells. Macrophages conditioned by ONECUT2-knockdown BC cells promoted proliferation and migration of BC cells but inhibited their apoptosis, whereas ONECUT2 overexpression exerted opposite effects (Figs. 5H–J and S21). Our study indicated that inhibition of ONECUT2 promoted macrophage polarization toward the M2 phenotype, highlighting its potential as a critical regulator of M2-type macrophage polarization and its role in modulating the efficacy of immunotherapy in BC.

### CCL28 is a direct target of ONECUT2 required for M2 macrophage polarization

**Fig. 6:**
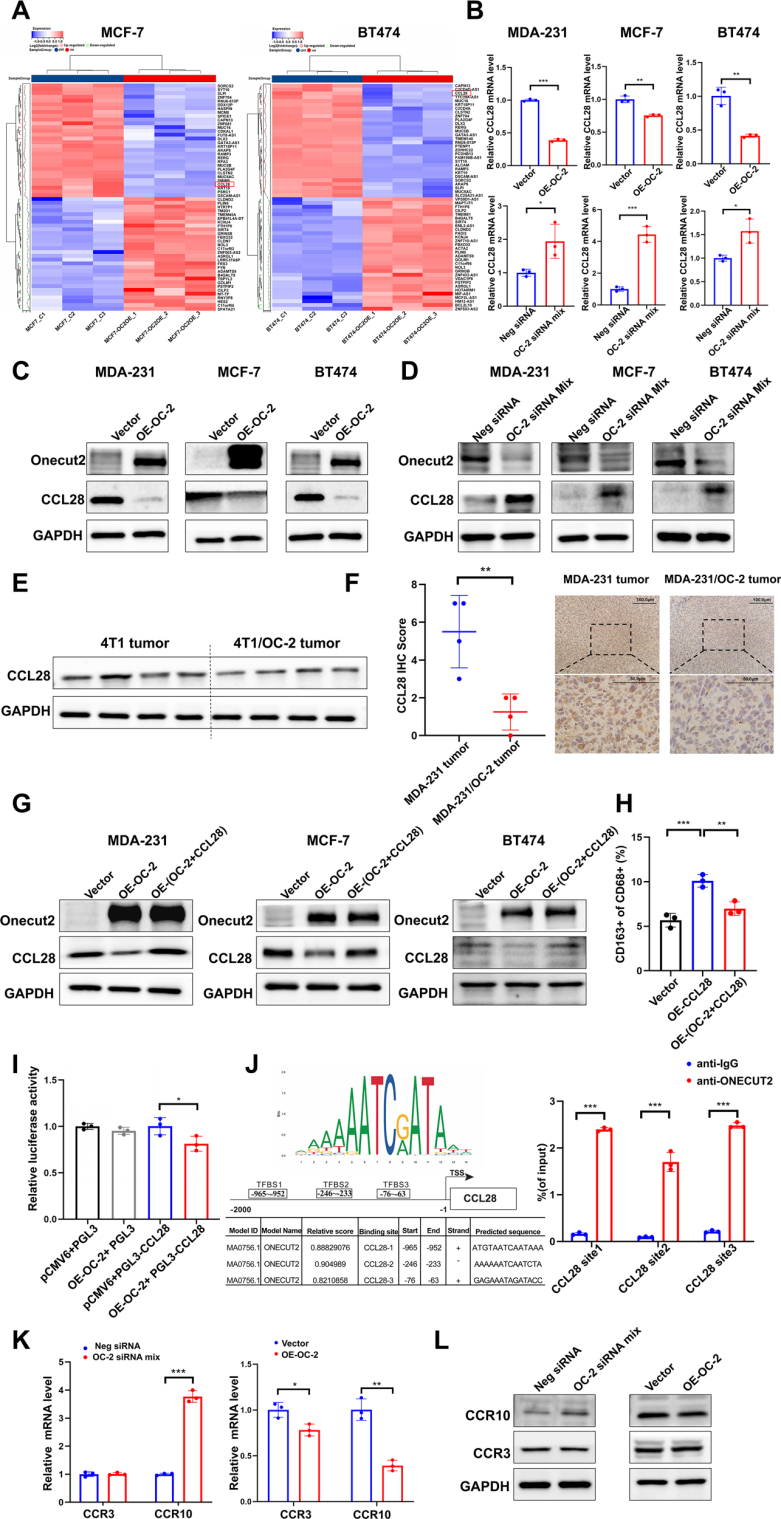
CCL28 is a direct target of ONECUT2 required for M2 macrophage polarization. **A** The publicly available RNA-seq data (GSE242541) was analyzed to identify altered genes following ONECUT2 overexpression in MCF-7 and BT474 cells. **B** RT-qPCR analysis for CCL28 mRNA levels with ONECUT2 overexpression (upper panel) or knockdown (lower panel) in BC cell lines. **C**, **D** Western blotting was used to detect the expression of CCL28 in breast cancer cells after overexpressing ONECUT2 (**C**) or silencing ONECUT2 (**D**). **E** CCL28 expression was evaluated by western blotting in the tumor tissues of BALB/c mouse models. **F** IHC analyses were performed to quantify CCL28 expression in MDA-231 (*n* = 4) or MDA-231/OC-2 (*n* = 4) xenograft tumors. **G** Western blotting analysis of CCL28 level in MDA-231 (Left panel), MCF-7 (Middle panel) and BT474 (Right panel) cells transfected with the indicated plasmids. **H** FCM analysis was used to explore the CD163^+^CD68^+^ population in THP-1 cells that had been primed with CM from treated MDA-231 cells. **I** Dual-luciferase reporter assay for detecting the activity of CCL28 promoters in MCF-7 cells transfected with ONECUT2 overexpression or control vector. **J** ONECUT2 motif logo and predicted binding sites in the CCL28 promoter (Left panel). ChIP-PCR confirmation of ONECUT2 binding to the CCL28 promoter in MCF-7 cells (Right panel). **K**, **L** RT-qPCR (**K**) and western blotting (**L**) of CCR3 and CCR10 in THP-1 cells treated with CM from MDA-231 cells with ONECUT2 knockdown (left panel) or overexpression (right panel). Error bars represent mean ± SD of three independent experiments *P < 0.05, **P < 0.01, ***P < 0.001.

To further investigate the regulatory mechanism of ONECUT2 in M2 macrophage polarization, we analyzed the publicly available RNA-seq data from Zamora et al. (GEO accession: GSE242541) [6] and observed that CCL28 was downregulated in MCF-7 and BT474 cells overexpressing ONECUT2 (Fig. 6A). Given that CCL28 [24] participates in BC progression by modulating proliferation, invasion, and metastasis, we investigated how ONECUT2 inhibition drives CCL28 expression and subsequently reprograms macrophages toward the M2 phenotype. Decreased CCL28 expression in response to ONECUT2 overexpression was confirmed in BC cell lines at both the RNA (Fig. 6B, Upper panel) and protein levels (Fig. 6C), whereas ONECUT2 deficiency produced the opposite effects (Fig. 6B Lower panel, 6D). ELISA confirmed that ONECUT2 knockdown significantly elevated the secretion of CCL28 (Fig. S22). Furthermore, CCL28 expression was downregulated in both 4T1/OC-2 tumors (Fig. 6E) and MDA-231/OC-2 xenografts (Fig. 6F) compared with their respective controls (4T1 and MDA-231). Western blotting analysis showed that CCL28 overexpression partially rescued the effect of ONECUT2 on CCL28 expression in MDA-231, MCF-7 and BT474 cells (Fig. 6G). Increased CCL28 expression in MDA-231 (Figs. 6H and S23A), MCF-7 (Fig. S23B) and BT474 cells (Fig. S23C) led to a higher CD163^+^ M2 macrophage population and elevated expression of M2 markers (CD163 and CD206), which were partially blocked by ONECUT2 overexpression (Fig. S24). Based on predictions of putative ONECUT2 binding sites in the CCL28 promoter from the JASPAR database, dual-luciferase reporter assays demonstrated that ONECUT2 significantly repressed the transcriptional activity of CCL28 in MCF-7 cells (Fig. 6I). ChIP-PCR further confirmed that ONECUT2 binds to the CCL28 promoter (Fig. 6J). Additionally, to investigate the receptors of CCL28 involved in the regulation of M2-type macrophage polarization, we examined the expression of CCR3 and CCR10 (known as CCL28 receptors). Knockdown of ONECUT2 in MDA-231 cells increased the RNA and protein levels of CCR10 in macrophages, whereas ONECUT2 overexpression induced the opposite effect. However, CCR3 protein levels in macrophages showed no significant changes when co-cultured with MDA-231 (Fig. 6K, L), MCF-7 (Fig. S25A), and BT474 (Fig. S25B) CM. The induction of CCR10 by CCL28 overexpression was reversed by ONECUT2 overexpression in BC cells (Fig. S25C–E). These findings suggest that CCR10 might act as a key molecule in ONECUT2-CCL28-mediated regulation of macrophage polarization to the M2 phenotype. Collectively, these results indicate that ONECUT2 transcriptionally represses CCL28, thereby suppressing macrophage polarization to an immunosuppressive M2-like phenotype. The ONECUT2-CCL28-CCR10 signaling axis reprograms macrophage polarization and modulates the TIME in BC.

## Discussion

Here, we demonstrate a specific mechanism by which ONECUT2 inhibition regulates cancer stemness characteristics and affects the immunosuppressive environment in BC. Our current study reveals novel mechanisms underlying cancer cell self-renewal capacity and immunoregulatory function, which alleviate chemoresistance and improve survival outcomes in BC patients. Growing evidence suggests that ONECUT2 is a multifaceted regulator of CSC properties and that its expression is significantly correlated with clinical prognosis [7, 25–27]. As shown in our previous study, chemotherapy-induced BC-derived EVs promote CSC traits and chemotherapy resistance by downregulating ONECUT2 expression. Other studies [4, 9] further reveal that ONECUT2 is an effective regulator in maintaining cancer stemness and chemoresistance, while the precise mechanism remains to be fully elucidated. Recent studies have shown that ONECUT2 is a master regulator of CSC properties through transcriptional reprogramming. ONECUT2 activates the transcriptional activity of TFPI in GC [8], thereby contributing to CSC traits and oxaliplatin resistance. Another study demonstrates that ONECUT2 inhibits PPP2R4 transcription [7], which in turn increases AKT/beta-catenin phosphorylation, thereby inducing GC stemness. Additionally, ONECUT2 was reported to be associated with the activation of the Wnt/beta-catenin signaling pathway [25]. The beta-catenin signaling was recognized as a core pathway in maintaining the CSC phenotype, with its target genes commonly serving as established stem-cell markers [28]. Modulation of homeostasis facilitates the balance of stem cell self-renewal [29]. In our current study, we identified beta-catenin as a central effector that mediates CSC maintenance and chemoresistance induced by ONECUT2 suppression. The functional heterogeneity of ONECUT2 across cancers, including its tumor-suppressive role identified in our study, possibly stems from tumor-specific microenvironments, dynamic signaling crosstalk, and interactions with tissue-specific cofactors. Survival analysis further indicated that reduced ONECUT2 expression correlated with poorer prognosis in breast cancer; most notably, its lower level in aggressive TNBC relative to favorable subtypes highlights its potential utility in patient stratification.

POU6F2 was originally cloned from the human retina and is known as retina-derived POU-domain factor-1 (RPF-1). The current understanding of POU6F2 is largely confined to the differentiation and morphogenesis of the kidney [30] and eye cornea [31]. Although several studies have elucidated the function of POU6F2 in tumor biology, its regulatory role in maintaining cancer stemness remains largely unexplored. POU6F2 exhibits dual regulatory roles in cancer, with tumor-promoting or tumor-suppressive functions influenced by microenvironmental factors across malignancies. In gastric adenocarcinoma with liver metastasis [32] and renal cell cancer [33], POU6F2 functions as an oncogene and is associated with worse clinical prognosis, aligning with our findings on its prognostic role in breast cancer. Conversely, POU6F2 exerts tumor-suppressive effects in Wilms tumor [34] and prolactinoma [35], highlighting its context-dependent dual role in cancer development. In this study, we first demonstrated that ONECUT2 inhibition activates the transcriptional activity of POU6F2, subsequently inducing CSC phenotype and chemotherapy resistance through a beta-catenin-dependent pathway, a mechanism that may explain the poorer clinical prognosis associated with low ONECUT2 and high POU6F2 expression.

We previously demonstrated that chemotherapy-induced EV-derived miRNAs downregulate ONECUT2, thereby reprogramming neighboring cells into a CSC phenotype [2]. Here, we identify the POU6F2-beta-catenin axis as the downstream mediator of this effect. Direct chemotherapy treatment does not consistently alter ONECUT2 or its targets, indicating the axis depends on EV cargo transfer from chemo-stressed to naïve cells. Given that this axis drives CSC traits, it represents an attractive therapeutic target. Beta-catenin, a key Wnt mediator, is a clinical oncology target with several agents in trials [36]. Although no direct POU6F2 therapeutics exist, its DNA-binding domain and protein-protein interaction sites are amenable to structure-guided drug design. Future therapeutic strategies could block EV miRNA-ONECUT2 signaling or target POU6F2/beta-catenin, potentially synergizing with conventional chemotherapy.

In clinical practice, breast cancer is classified into four molecular subtypes based on immunohistochemical results: Luminal A, Luminal B, HER2-positive and TNBC. The expression pattern of low ONECUT2 and high POU6F2/beta-catenin, which was most evident in aggressive subtypes (TNBC/HER2-positive), served as a key predictor of poorer clinical prognosis in our study. The low ONECUT2-POU6F2-beta-catenin axis presents a subtype-specific prognostic system, in which distinct expression signatures differentially modulate survival outcomes, warranting further investigation. In addition, dynamic changes in these markers may serve as potential biomarkers for treatment response. For instance, alterations in exosomal miRNAs targeting ONECUT2, or increased POU6F2/beta-catenin expression in biopsies, may provide early warning of emerging resistance, thereby enabling timely therapeutic adjustments before clinical progression.

The immunosuppressive TIME is a major barrier to effective cancer immunotherapies. Reprogramming TAMs into an antitumor phenotype is a promising strategy. Studies [37, 38] have revealed that TAMs engage in functional crosstalk with the CSC phenotype. TAMs undergo preferential polarization toward the M2 phenotype, which acquires potent immunosuppressive activity that mediates pro-tumorigenic functions in BC [39]. This is exemplified by findings that TAMs-derived EVs shaped the immunosuppressive microenvironment in colorectal liver metastases by increasing the stemness potential of cancer cells, thereby dampening the efficacy of anti-PD-1 immunotherapy [40]. Given this rationale, therapies directly targeting TAMs are being actively investigated as a potential treatment strategy [41, 42]. Therefore, elucidating the regulatory mechanisms within the TAM-CSC network may facilitate the identification of novel synergistic targets and predictive biomarkers for immunotherapy and chemotherapy, offering therapeutic avenues for breast cancer treatment. In our study, we analyzed the changes in various immunocyte subsets in 4T1/OC-2 tumor-bearing BALB/c mice and found a significant reduction in macrophage polarization towards the M2 type. Functionally, as evidenced by the RNA-Sequencing results shown in Fig. 6A, we figured out that CCL28 was identified as a key factor in mediating the suppressive effect of ONECUT2 on M2 macrophage polarization. ONECUT2 knockdown leads to transcriptional activation of CCL28, subsequently inducing macrophage polarization via the CCL28-CCR10 signaling axis and thereby creating an immunosuppressive TIME in BC. CCL28, a member of the CC chemokine subfamily, has been implicated in the promotion of tumor progression through immune regulation and EMT modulation [43, 44]. High CCL28 expression is associated with poor patient survival, supporting its role in mediating treatment resistance and adverse clinical outcomes [45]. While a previous study [46] on colorectal cancer reported that CCL28 suppressed macrophage polarization to the M2 phenotype and inhibited tumor progression, our findings revealed a paradoxical pro-tumorigenic role in BC, where CCL28 upregulation enhanced M2-type polarization through a CCR10-dependent mechanism. This discrepancy may stem from the heterogeneity of the TIME and the presence of other mechanisms and factors that influence the interplay between CCL28-M2 macrophage, which requires further exploration.

**Fig. 7:**
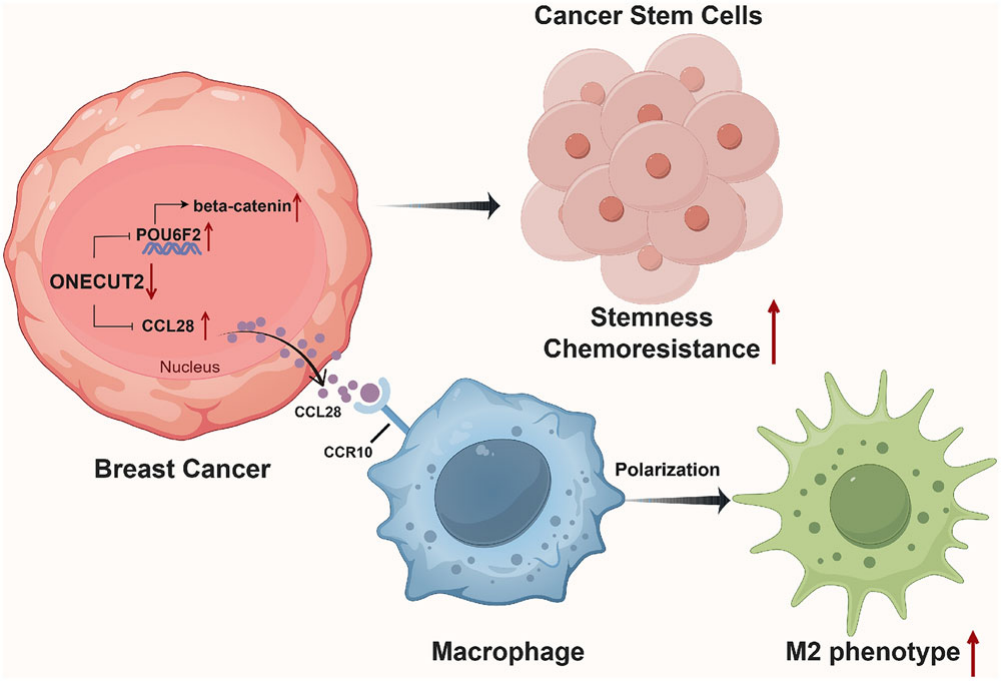
Schematic diagram. The inhibition of ONECUT2 induces breast cancer stemness characteristics and chemoresistance via the POU6F2-beta-catenin axis and facilitates macrophage polarization to M2 phenotype through the CCL28–CCR10 pathway.

Our findings are the first to demonstrate the specific characteristics of ONECUT2 that shape transcriptional profiles linked to CSC traits and TIME in BC (Fig. 7). Conceivably, targeting pivotal molecules in the ONECUT2-POU6F2-beta-catenin and ONECUT2-CCL28-CCR10 signaling pathways could reduce cancer cell stemness as well as enhance the efficacy of chemotherapy and immunotherapy in BC.

## Supplementary information

Supplementary Information

Table S1

Table S2

Table S3

Original Western blotting images

## Data Availability

The data generated in this study are available upon request from the corresponding author.
