# Supplementary material for: Low expression of ONECUT2 governs the POU6F2-beta-catenin axis to modulate cancer stemness and drives the CCL28-dependent pathway for macrophage polarization in breast cancer
Source: Cell Death Dis. 2026 Jun 2;17(1):690. doi: 10.1038/s41419-026-08909-5 (PMC13443588; doi:10.1038/s41419-026-08909-5)

Fig1A

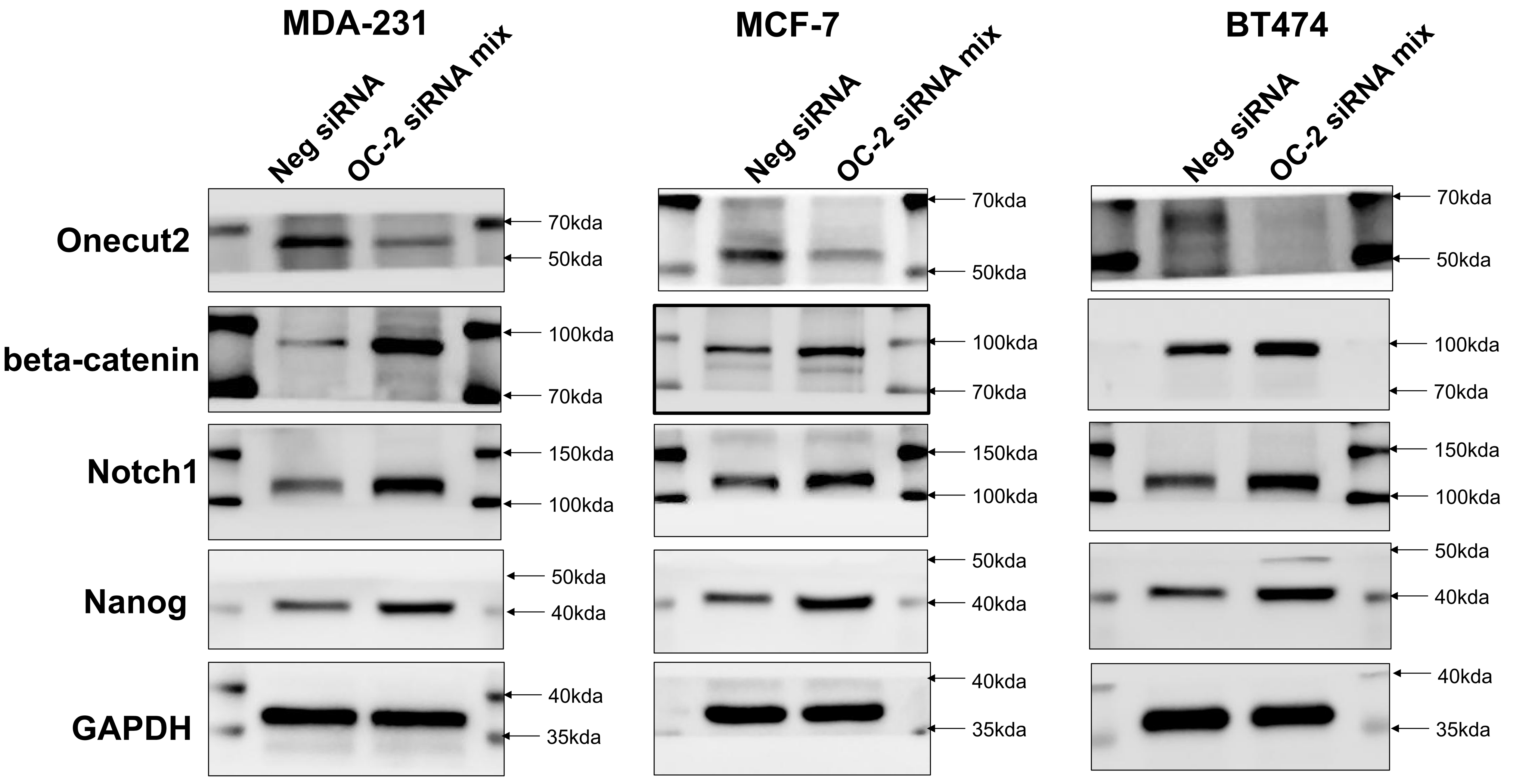

Fig1B

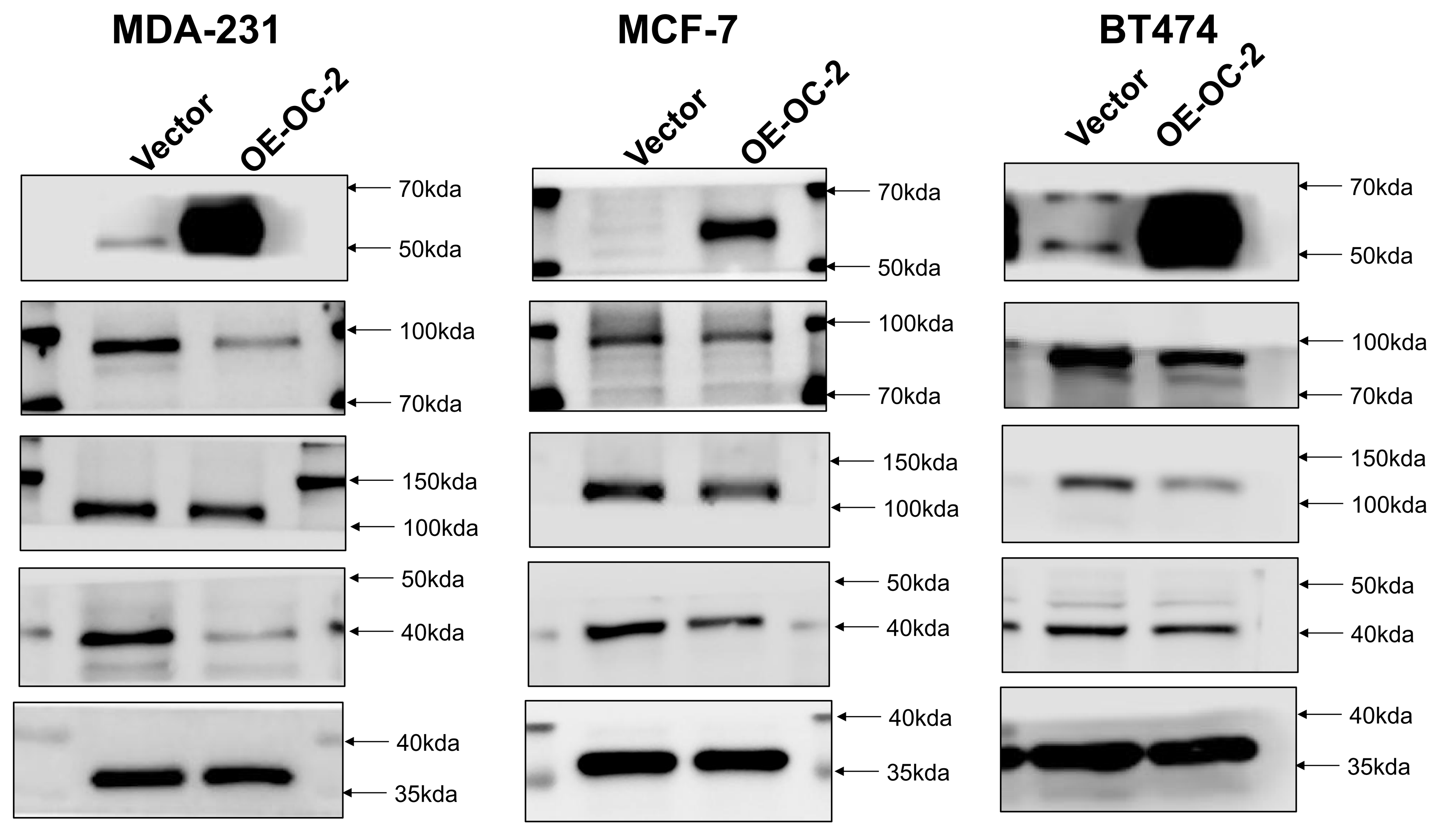

Fig1C

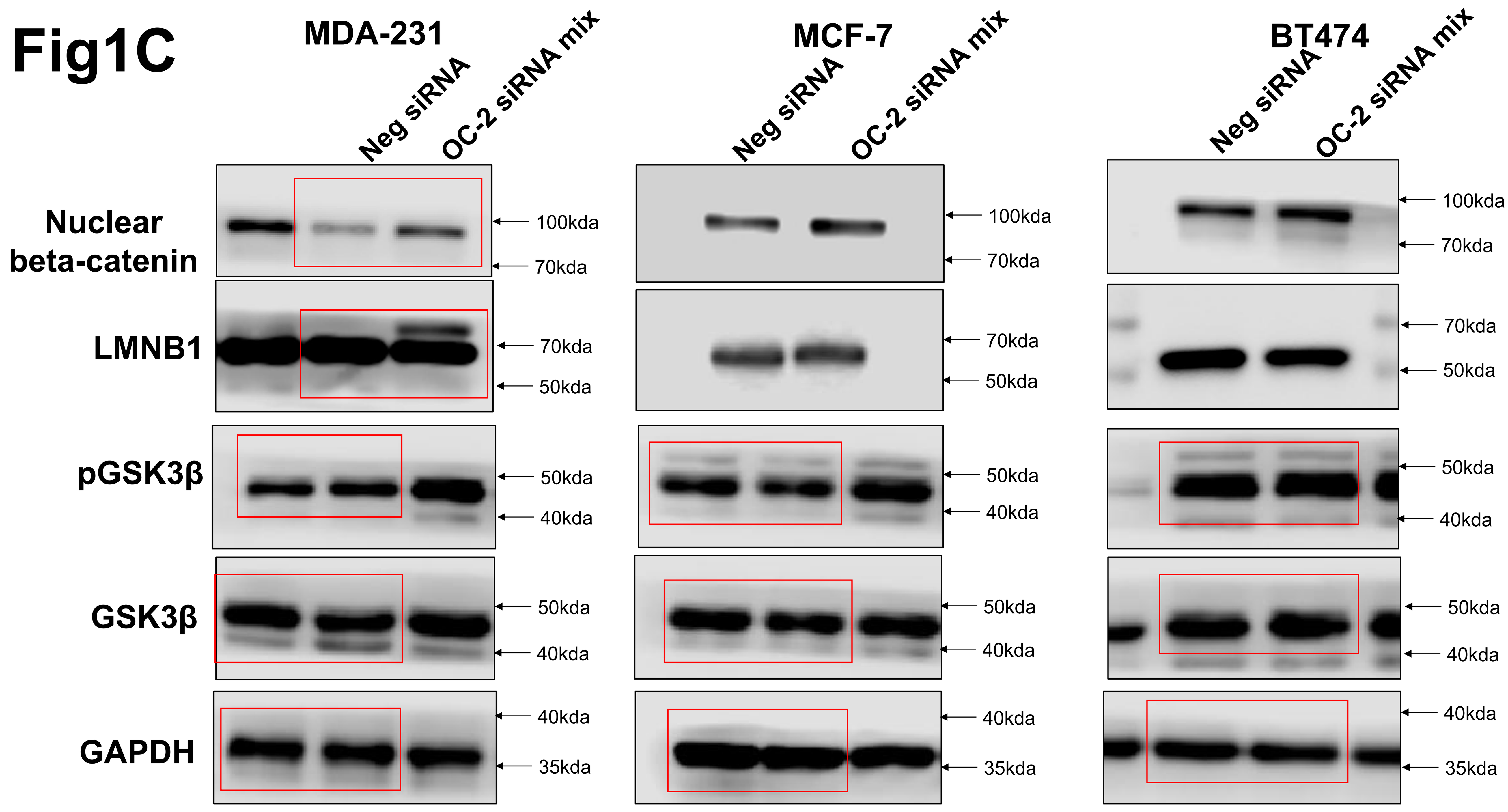

Fig1F

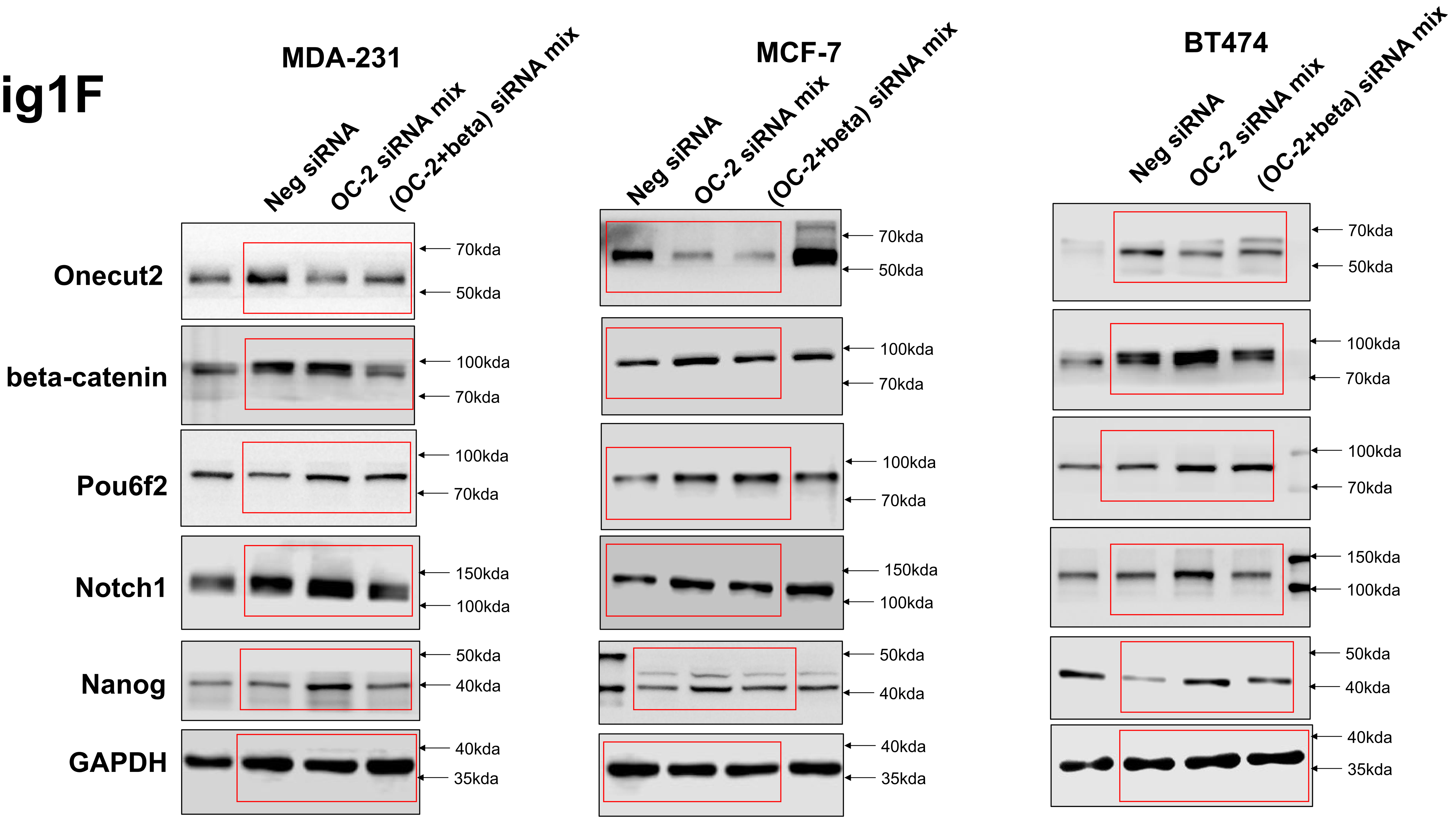

Fig1J

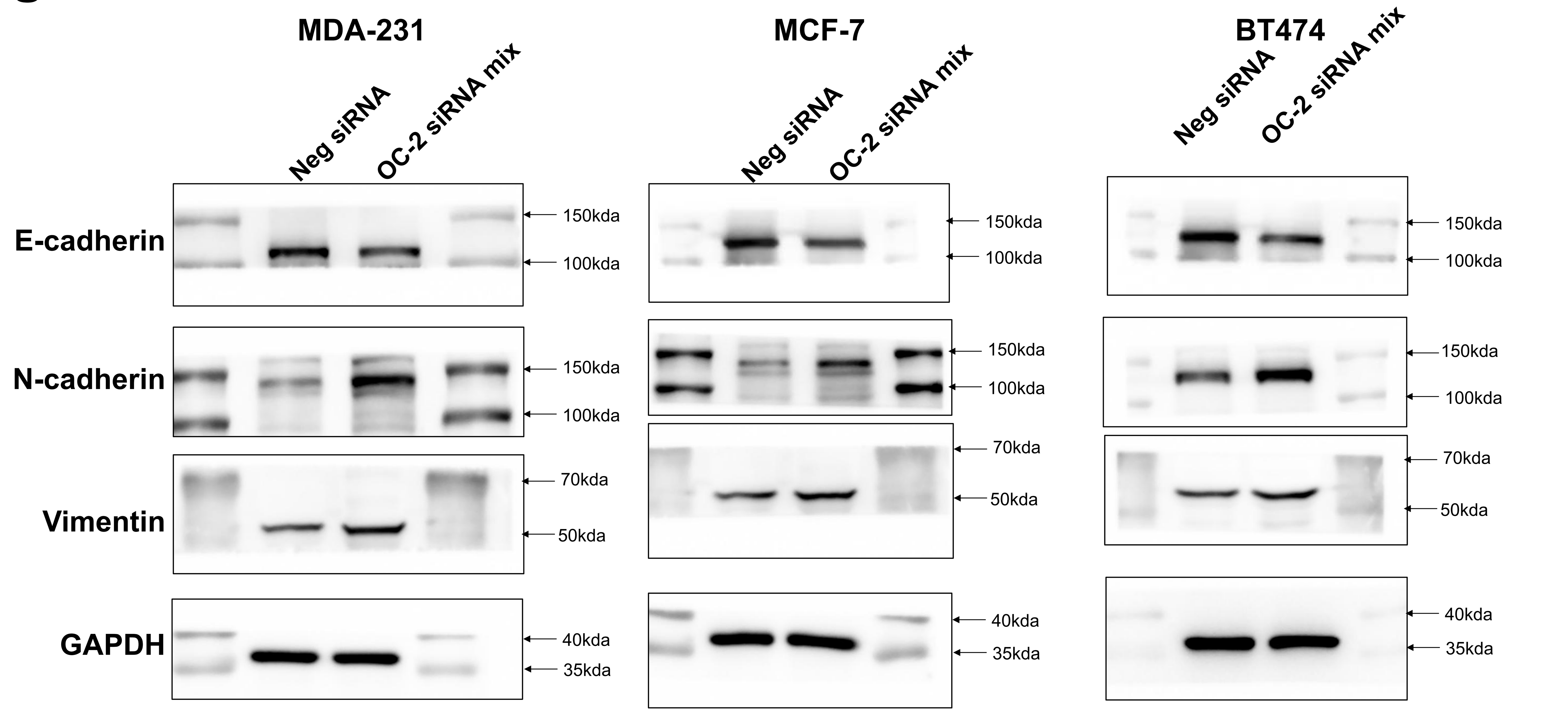

Fig1K

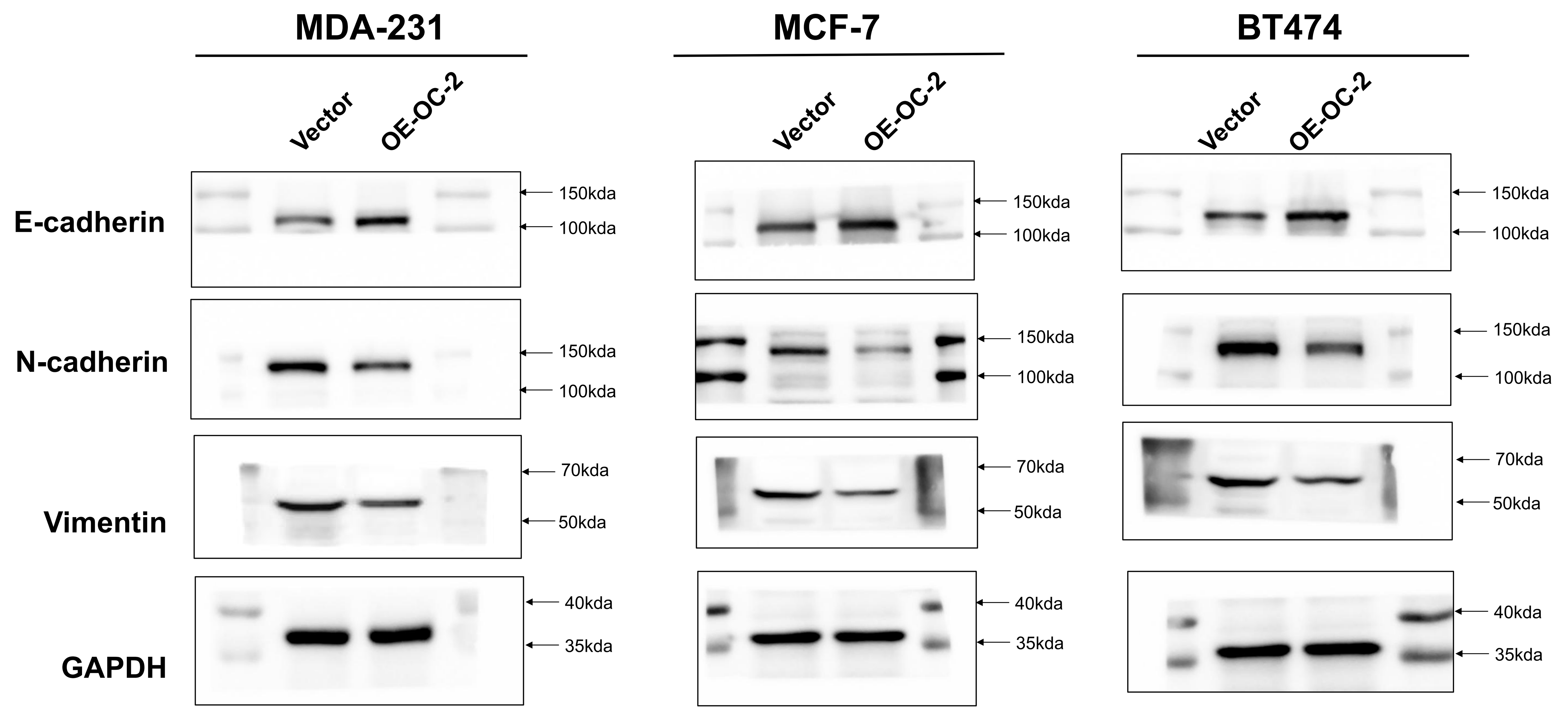

Fig2A

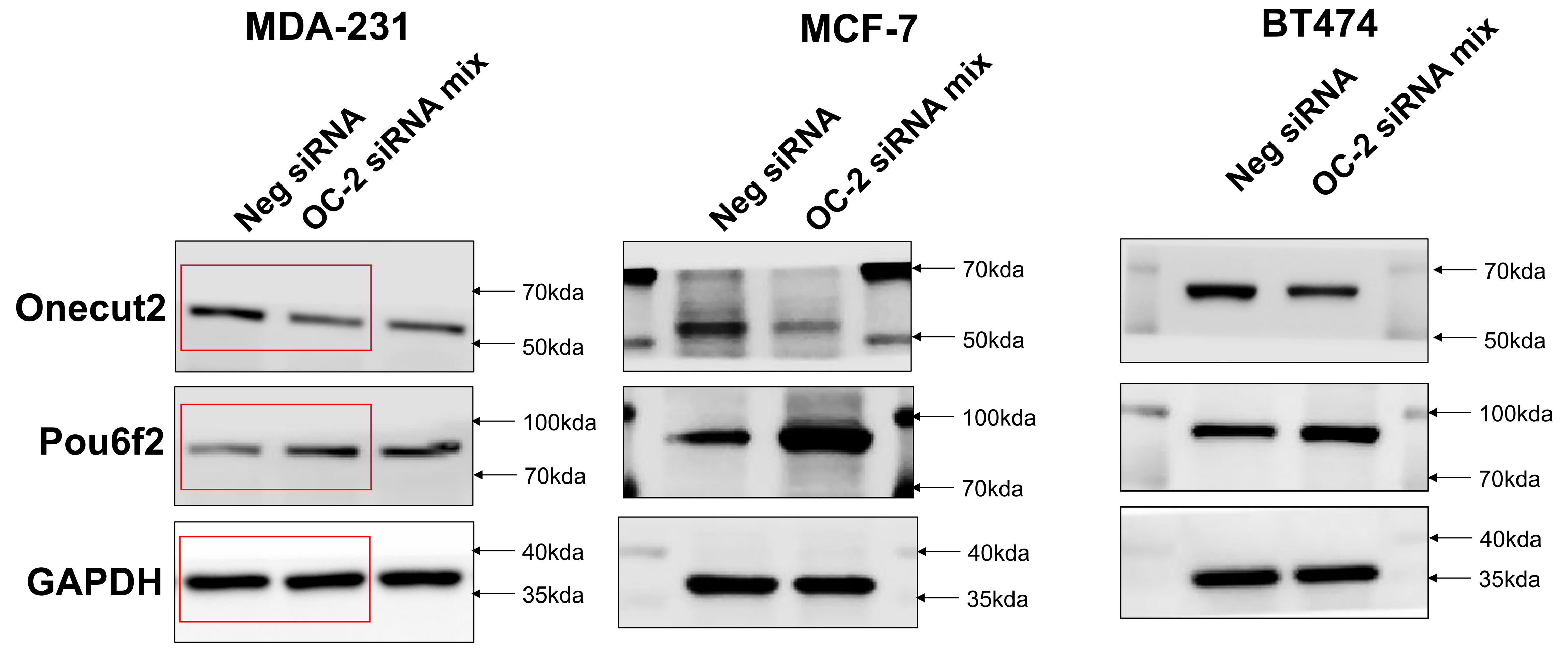

Fig2B

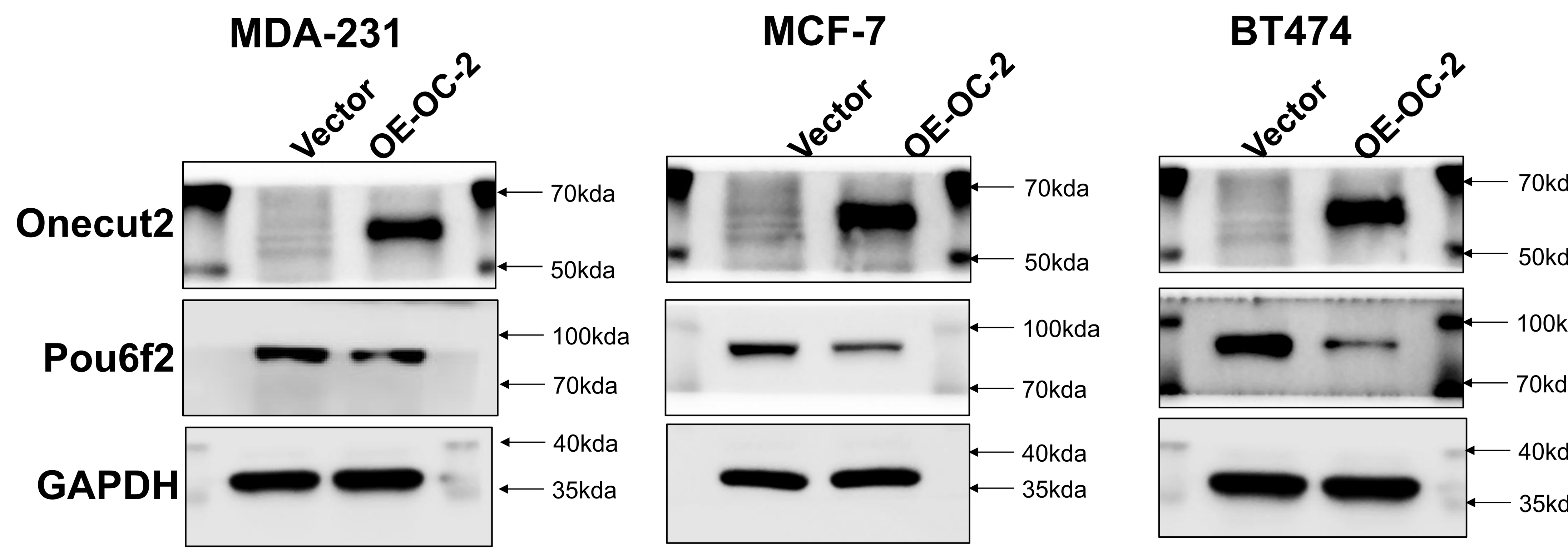

Fig3A

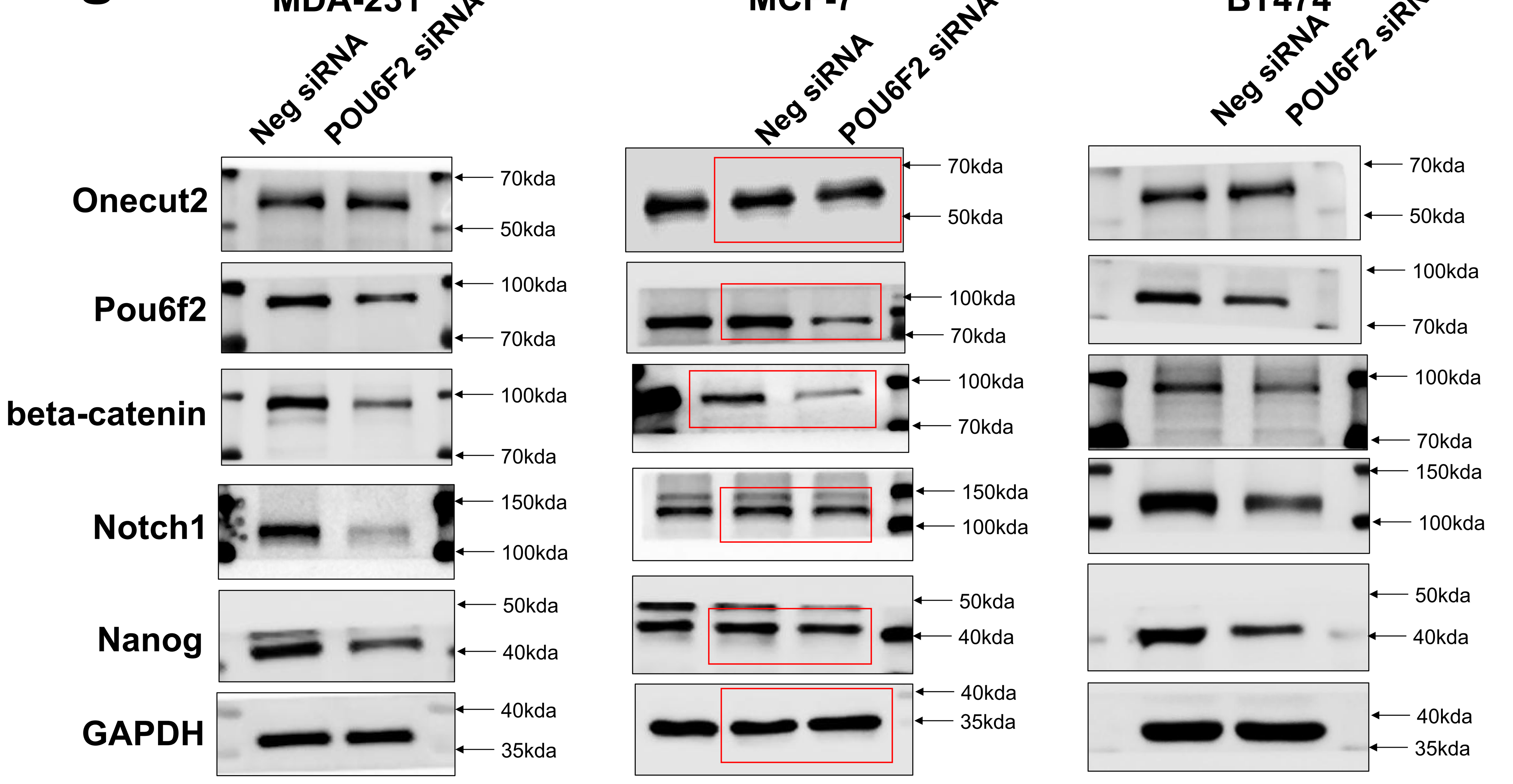

Fig3B

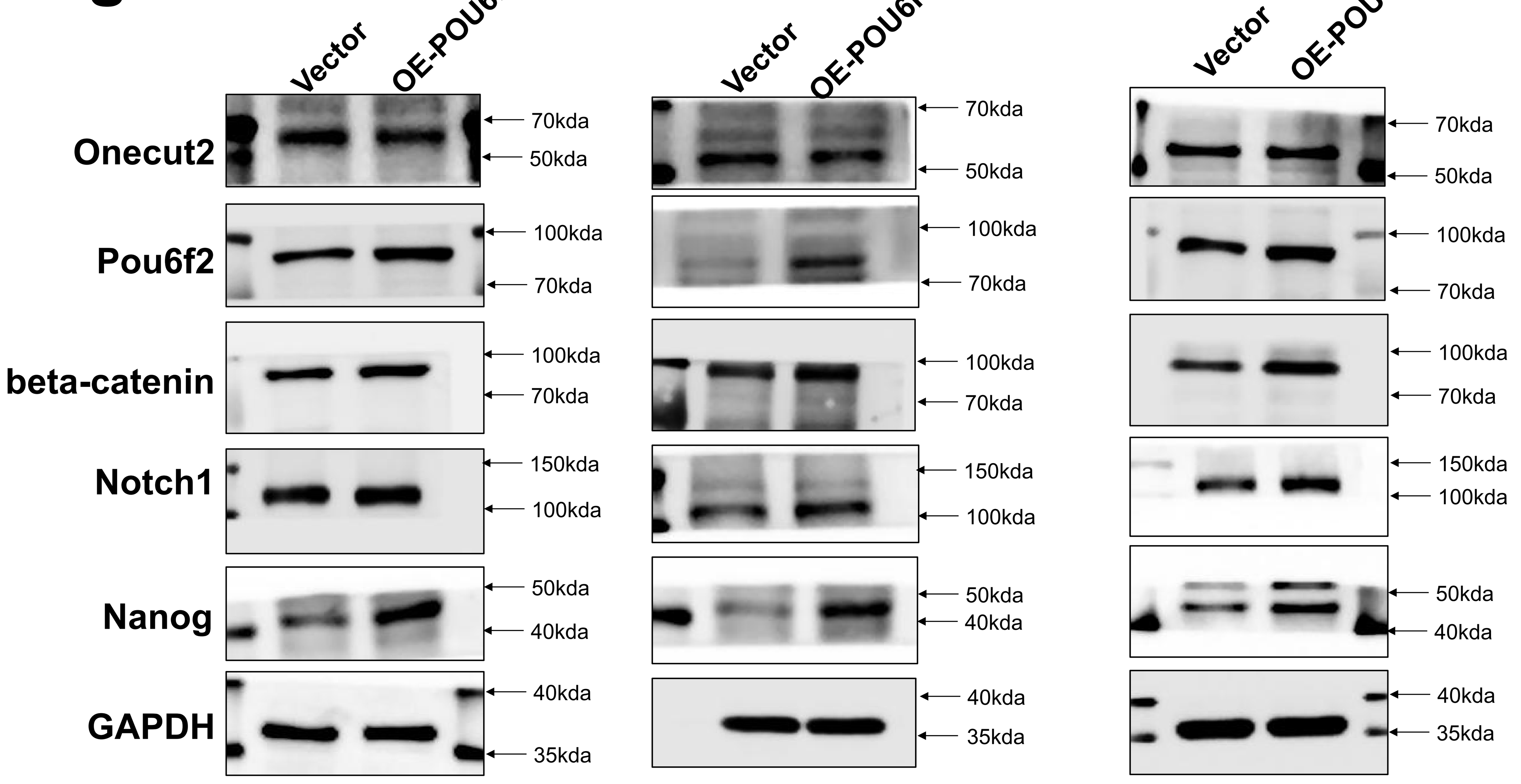

Fig3F

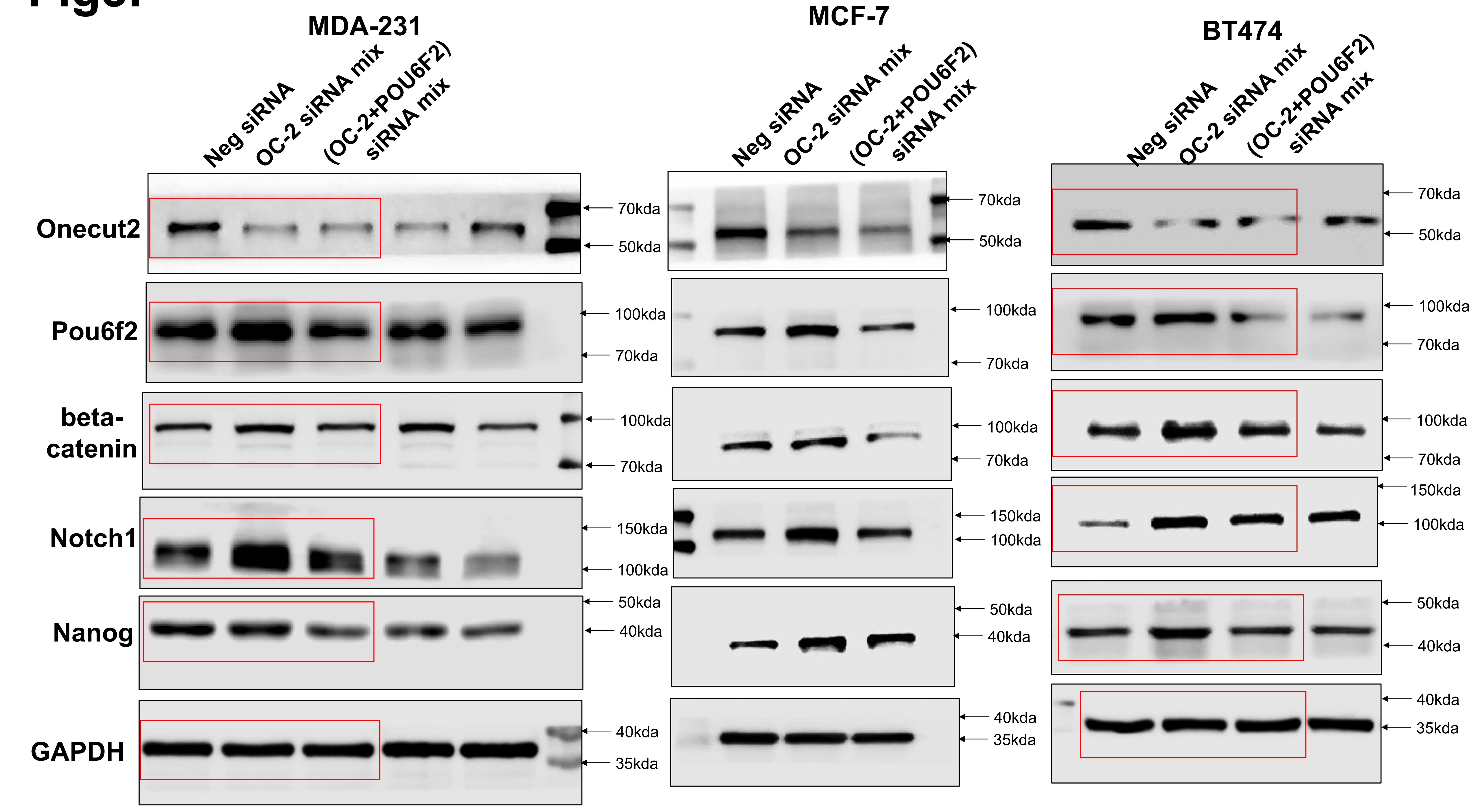

Fig3G

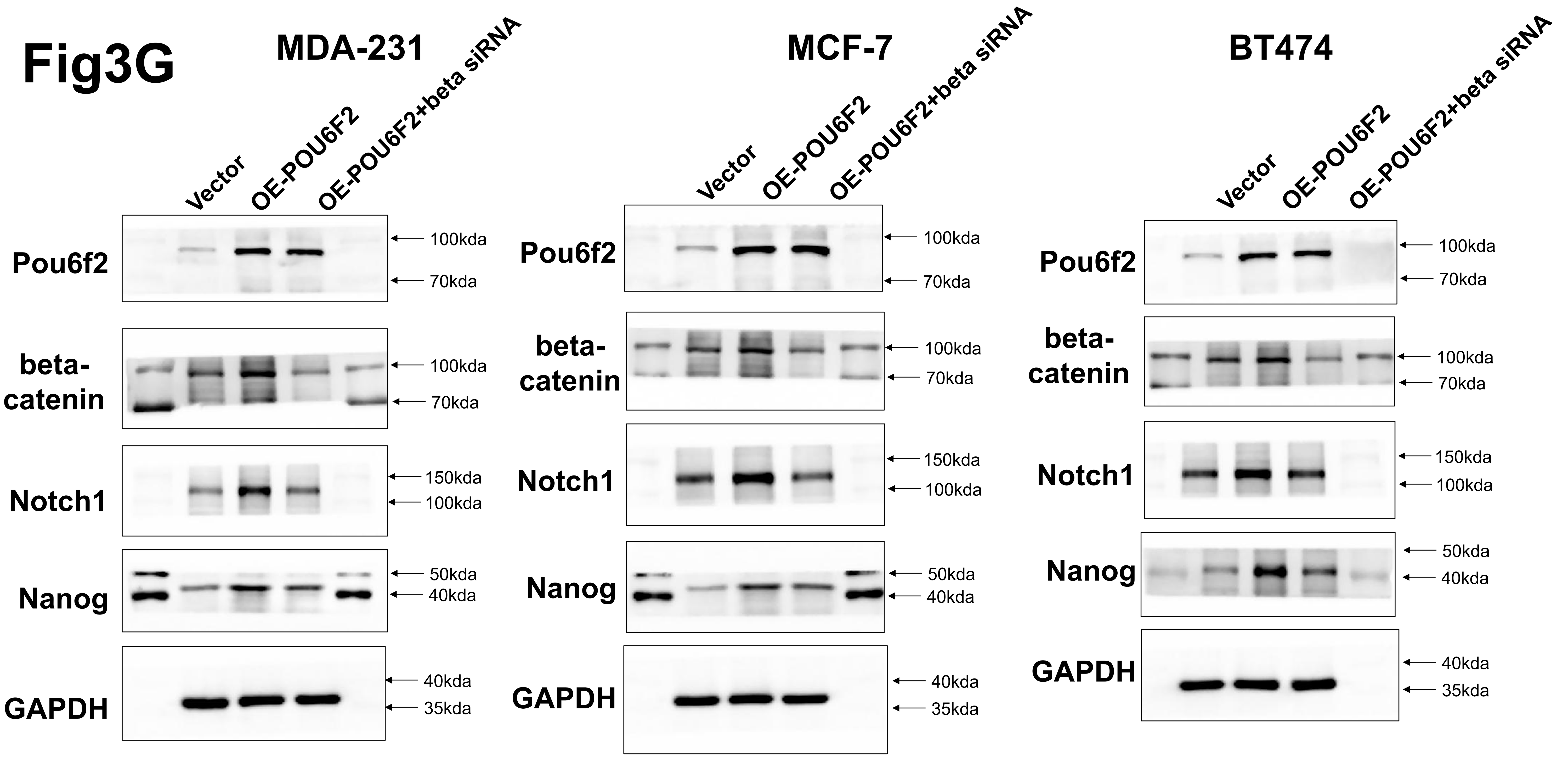

Fig4A

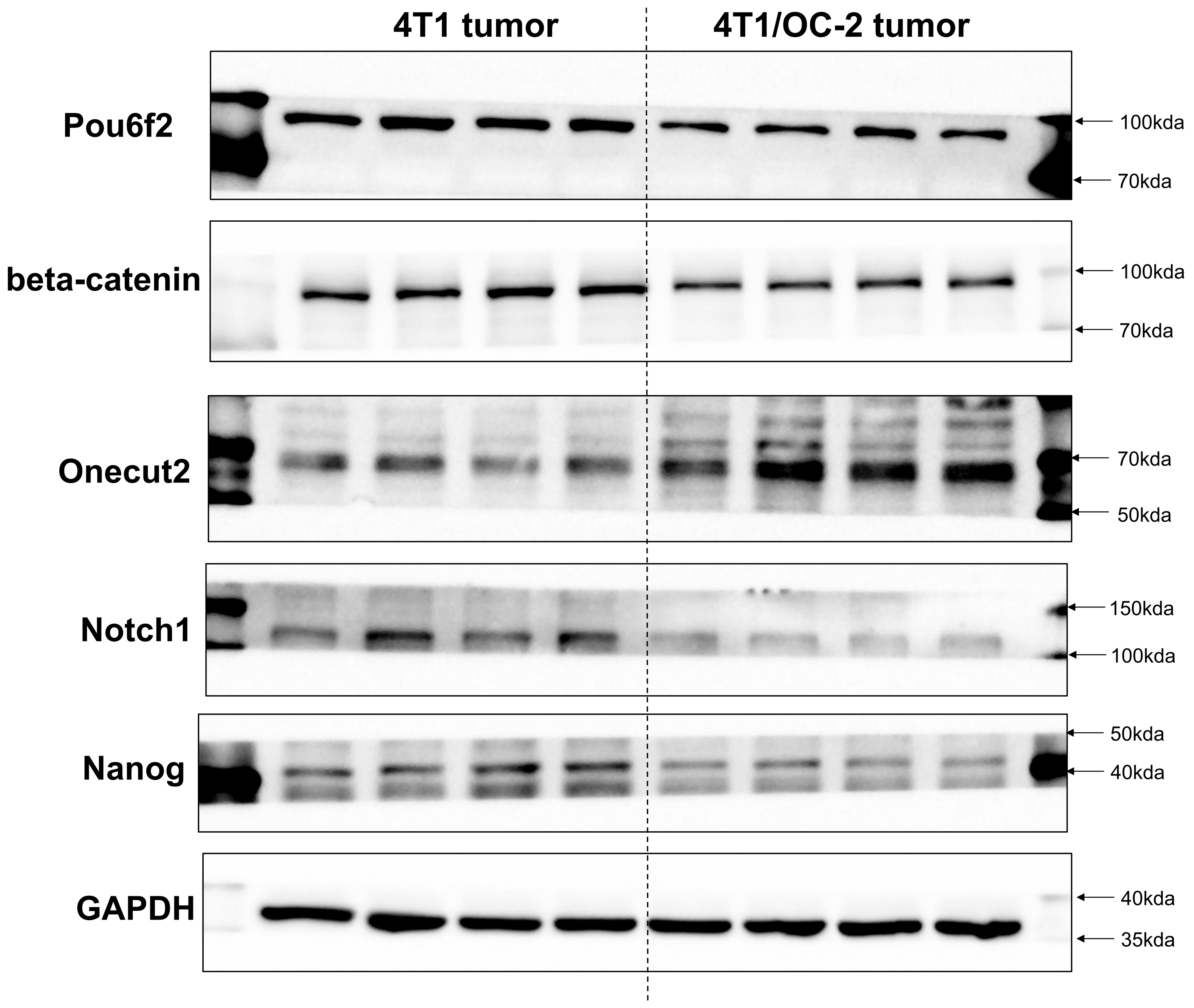
4T1 tumor
4T1/OC-2 tumor
Pou6f2
beta-catenin
Onecut2
Notch1
Nanog
GAPDH
100kda
70kda
100kda
70kda
70kda
50kda
150kda
100kda
50kda
40kda
40kda
35kda

Fig4B

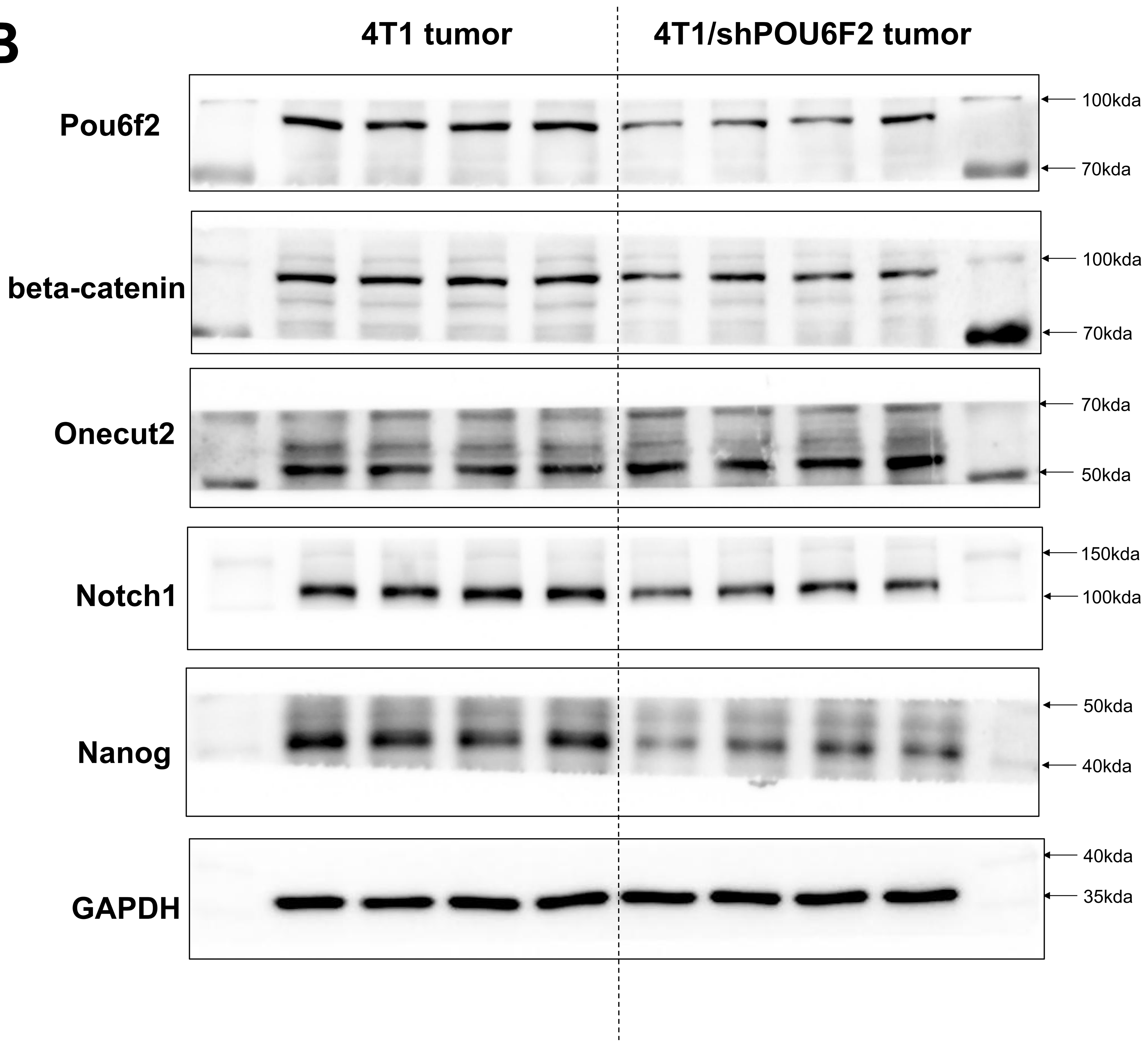
4T1 tumor
4T1/shPOU6F2 tumor
Pou6f2
beta-catenin
Onecut2
Notch1
Nanog
GAPDH
100kda
70kda
100kda
70kda
70kda
50kda
150kda
100kda
50kda
40kda
40kda
35kda

Fig4C

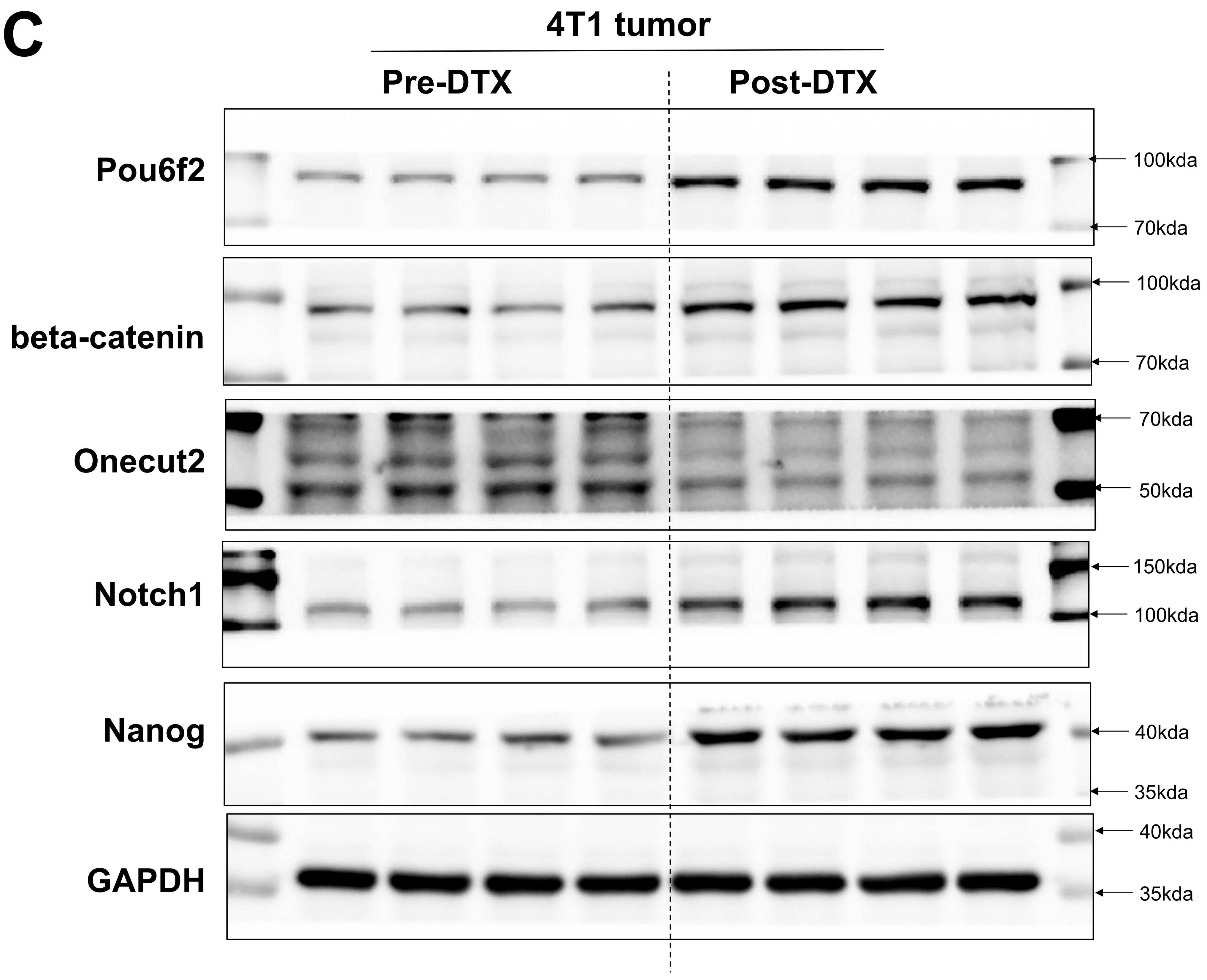
4T1 tumor
Pre-DTX
Post-DTX
Pou6f2
beta-catenin
Onecut2
Notch1
Nanog
GAPDH
100kda
70kda
100kda
70kda
70kda
50kda
150kda
100kda
40kda
35kda
40kda
35kda

Fig4C

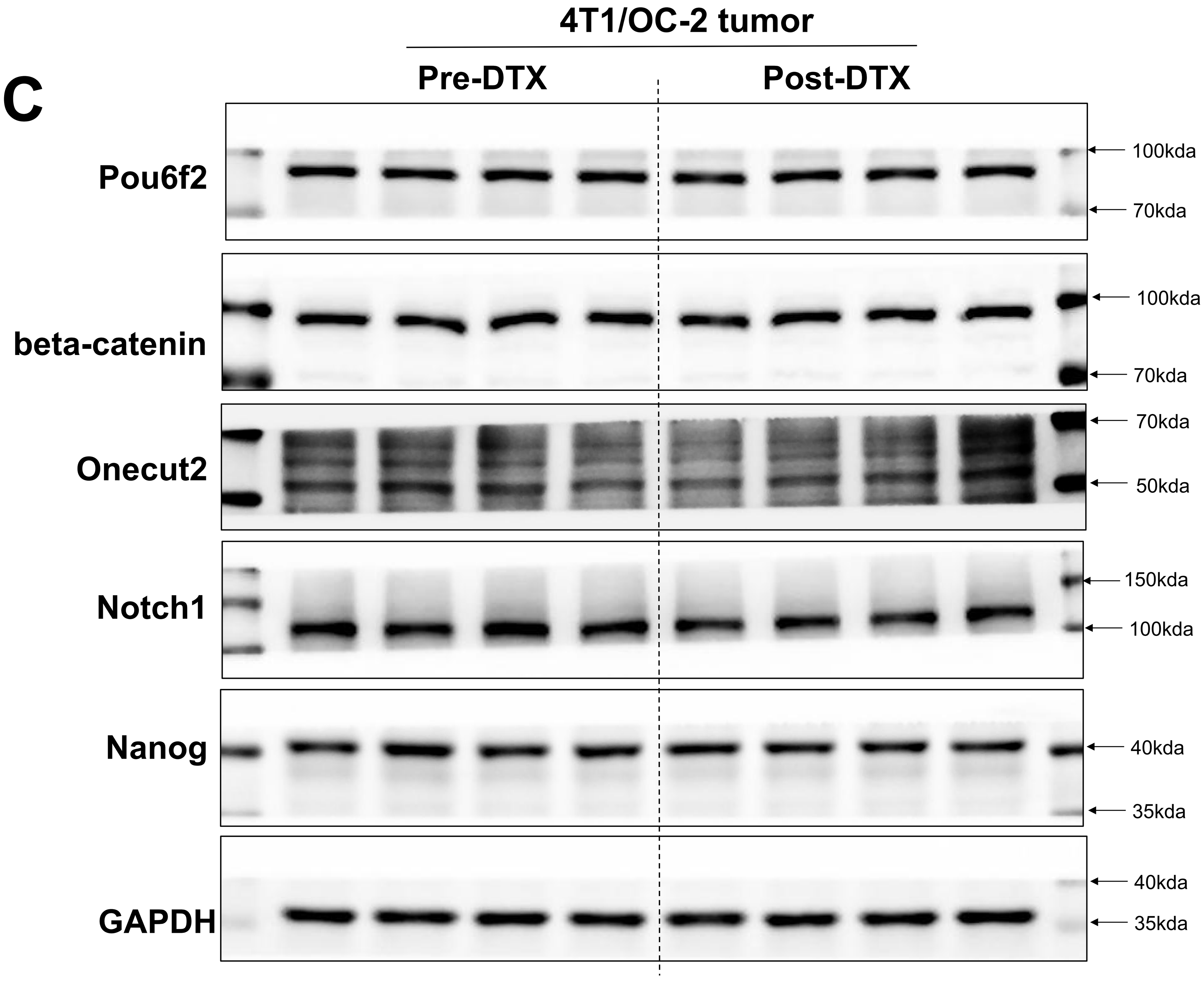
4T1/OC-2 tumor
Pre-DTX
Post-DTX
Pou6f2
beta-catenin
Onecut2
Notch1
Nanog
GAPDH
100kda
70kda
100kda
70kda
70kda
50kda
150kda
100kda
40kda
35kda
40kda
35kda

Fig4D

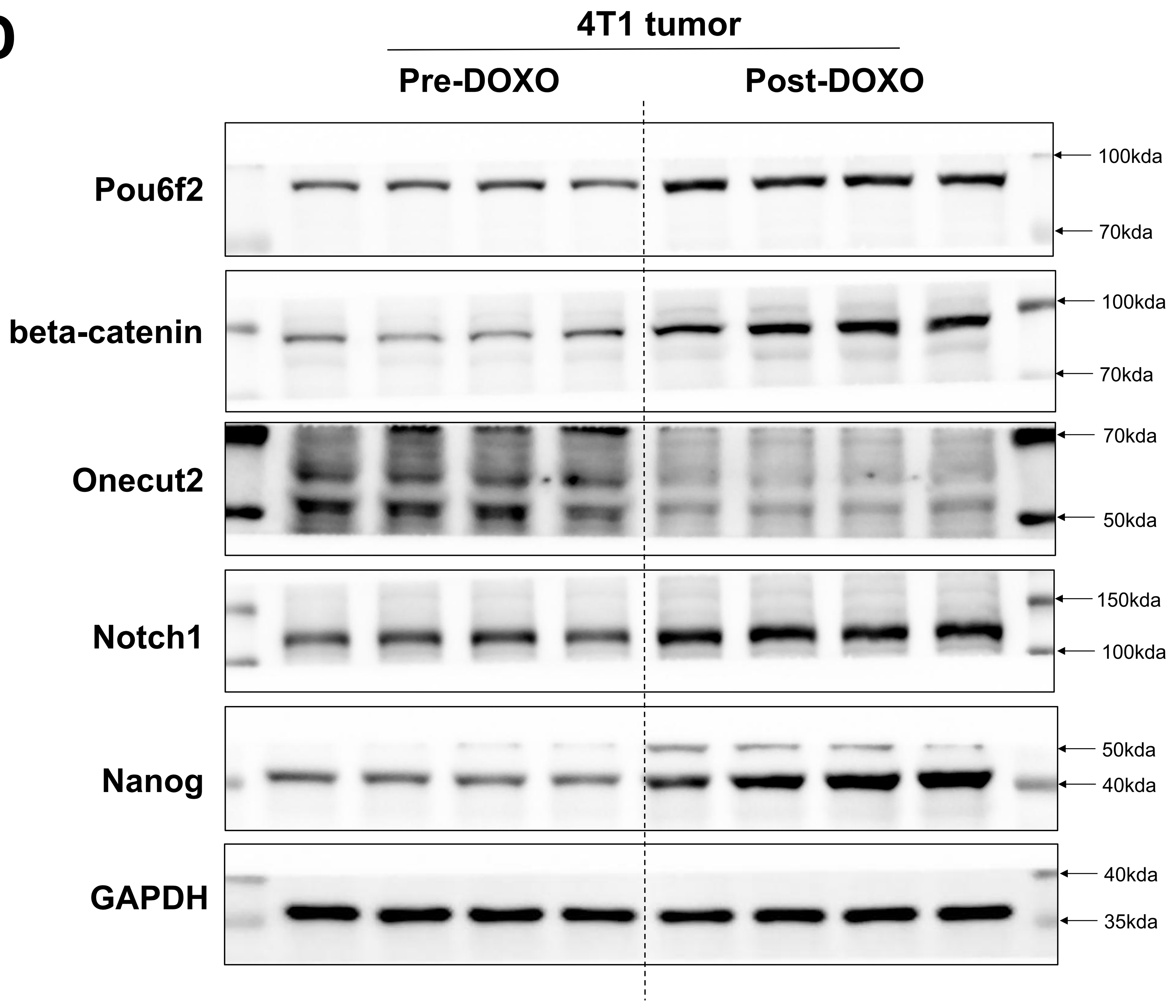

Fig4D

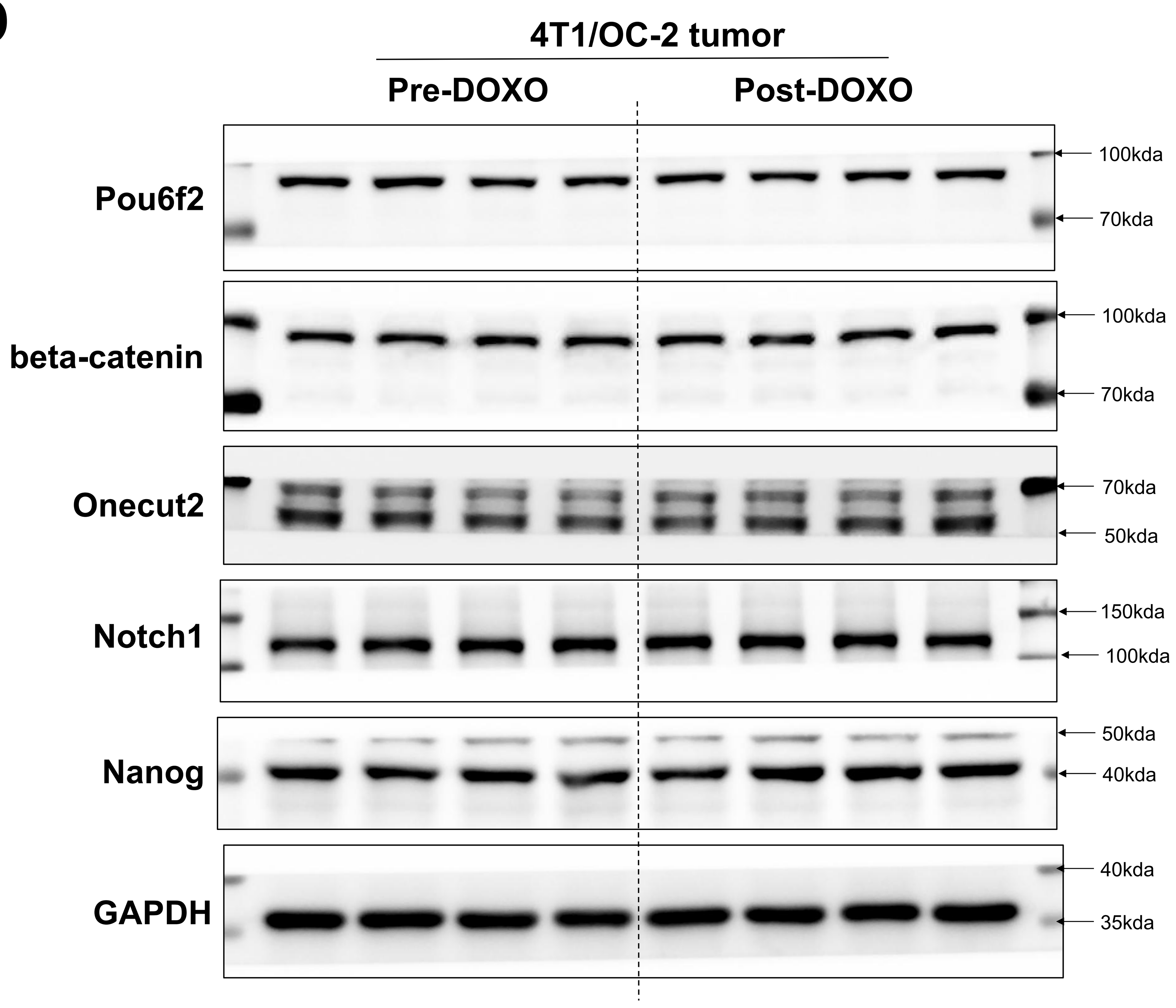

Fig4E

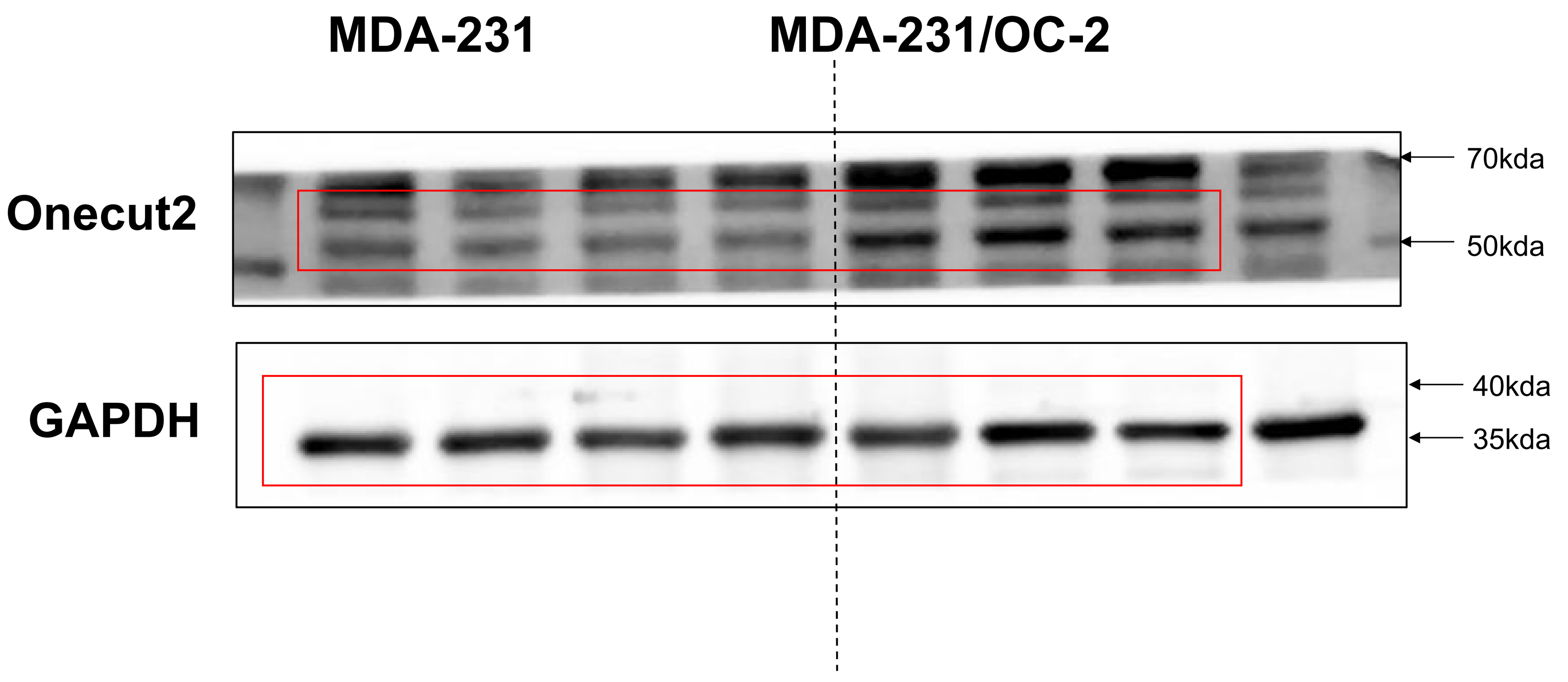

Fig4F

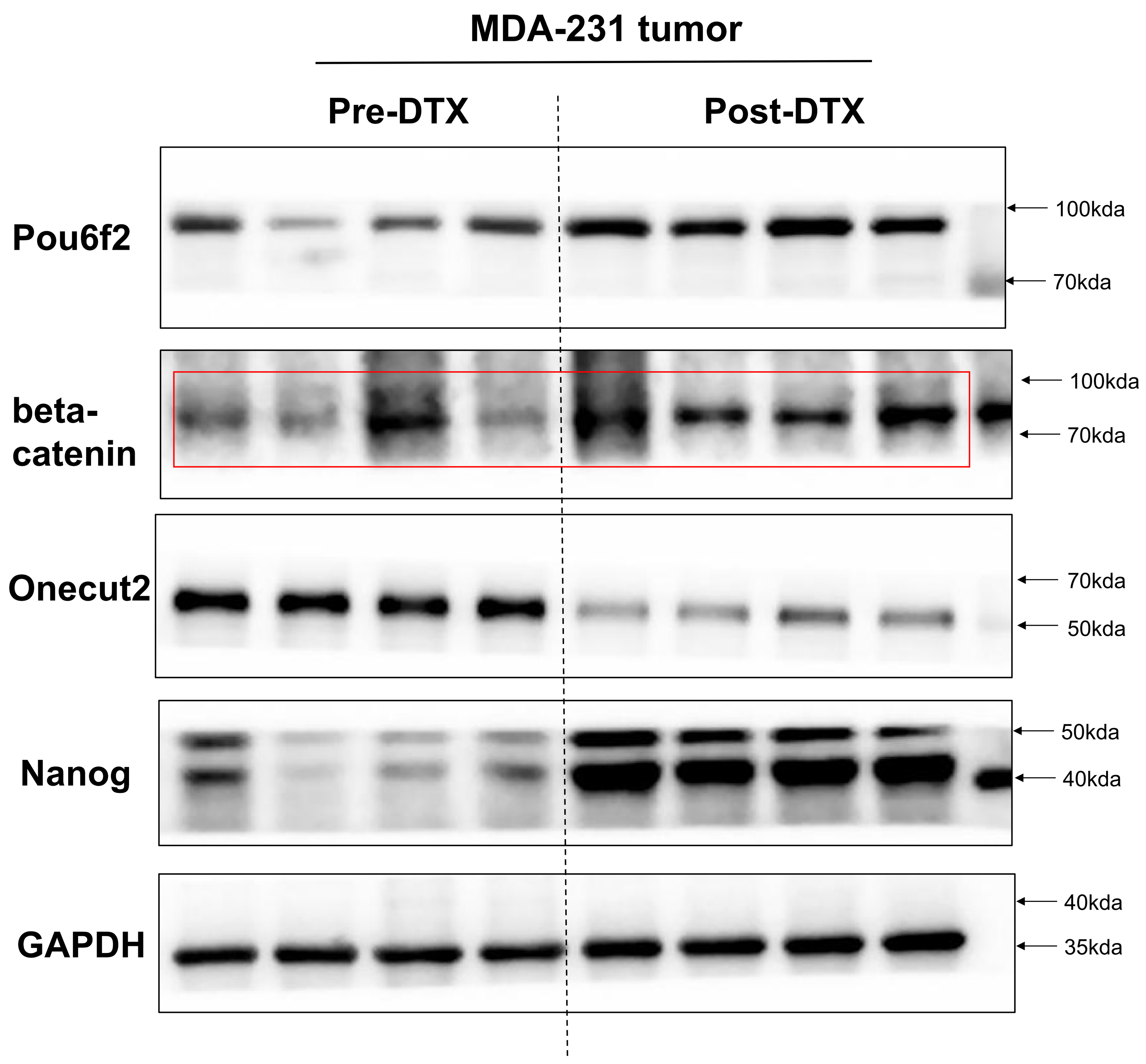
MDA-231 tumor
Pre-DTX
Post-DTX
Pou6f2
beta-catenin
Onecut2
Nanog
GAPDH
100kda
70kda
100kda
70kda
70kda
50kda
50kda
40kda
40kda
35kda

Fig4F

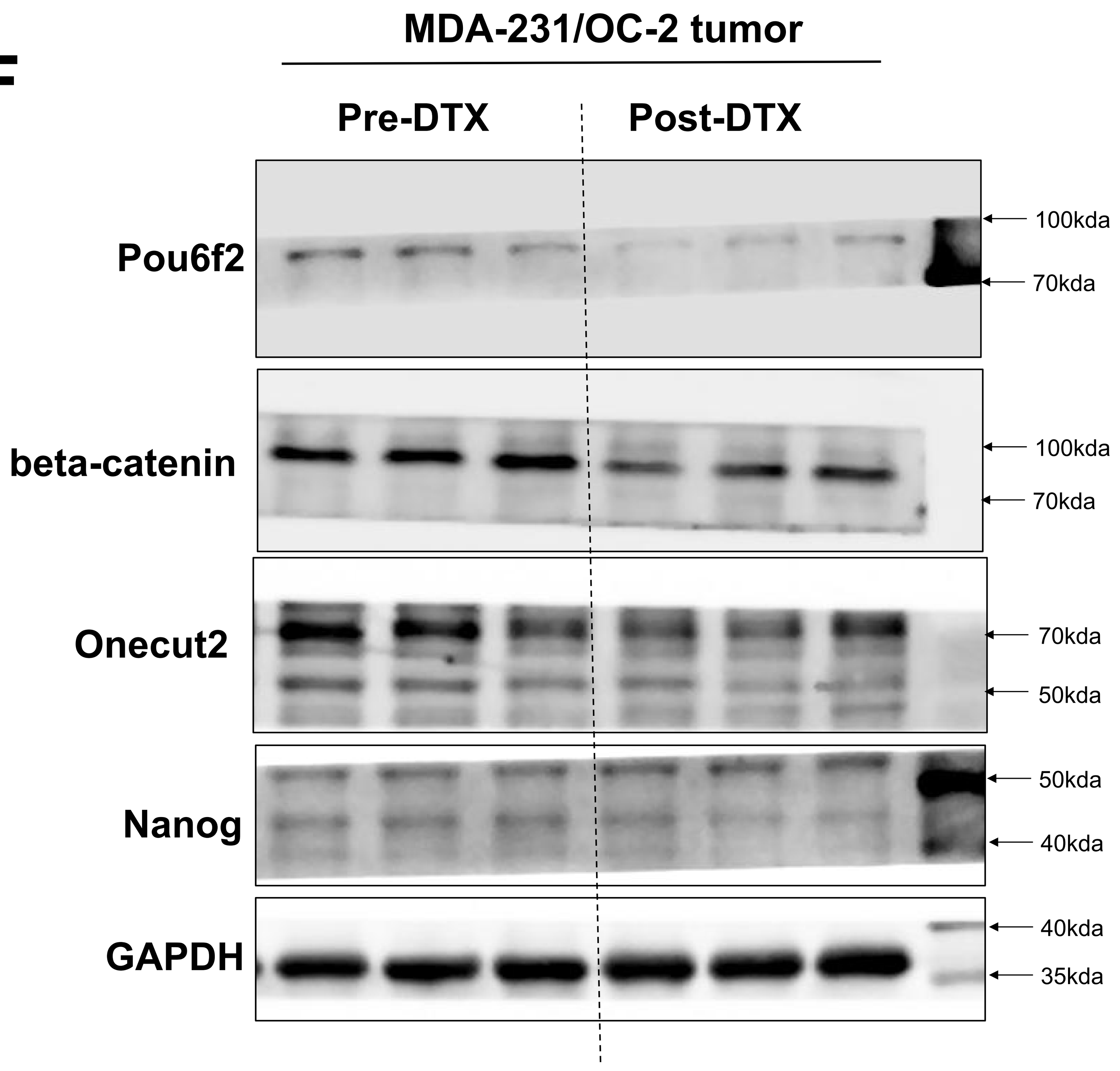
MDA-231/OC-2 tumor
Pre-DTX
Post-DTX
Pou6f2
beta-catenin
Onecut2
Nanog
GAPDH
100kda
70kda
100kda
70kda
70kda
50kda
50kda
40kda
40kda
35kda

Fig5F

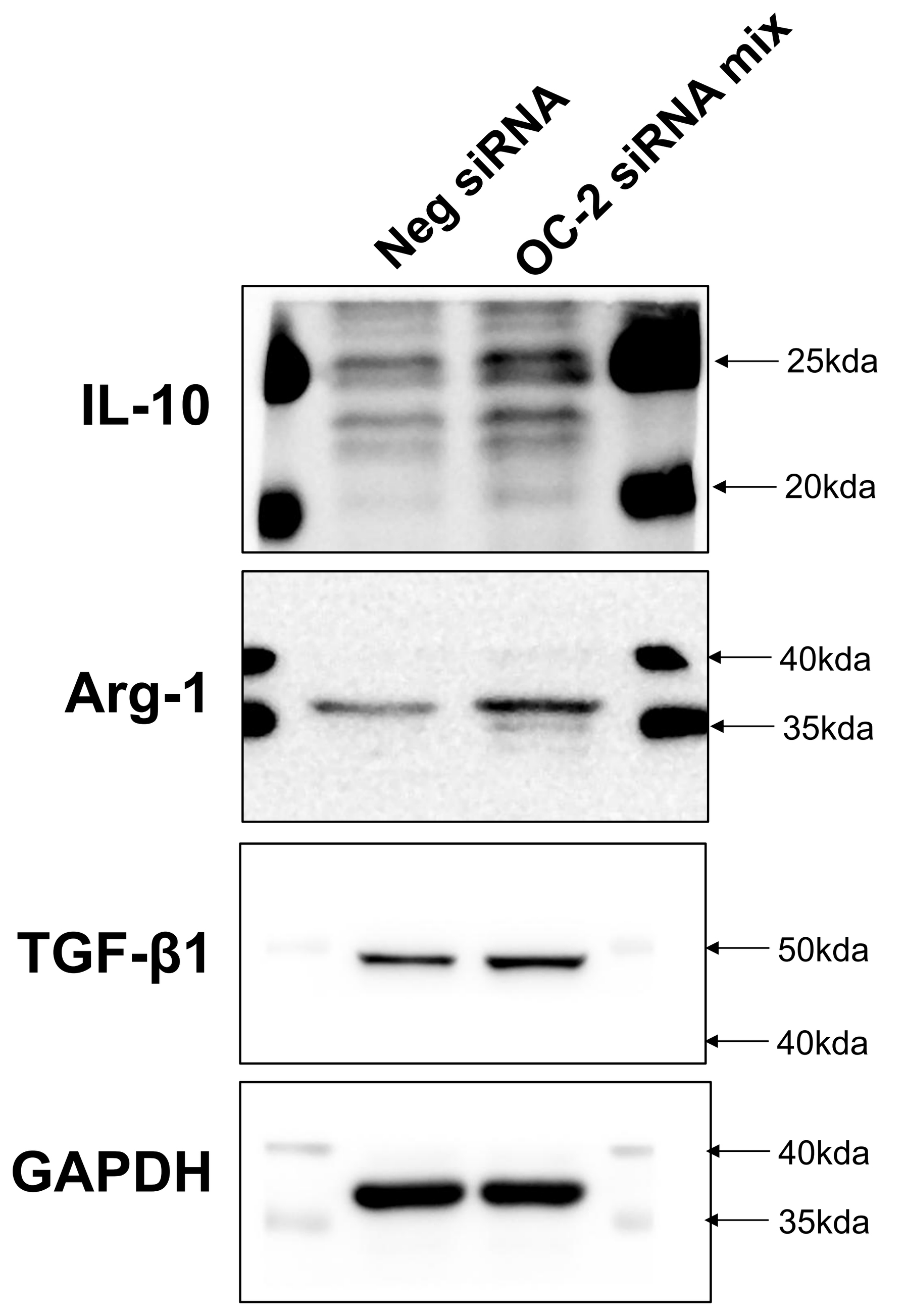
Neg siRNA
OC-2 siRNA mix
IL-10
25kda
20kda
Arg-1
40kda
35kda
TGF-β1
50kda
40kda
GAPDH
40kda
35kda

Fig5G

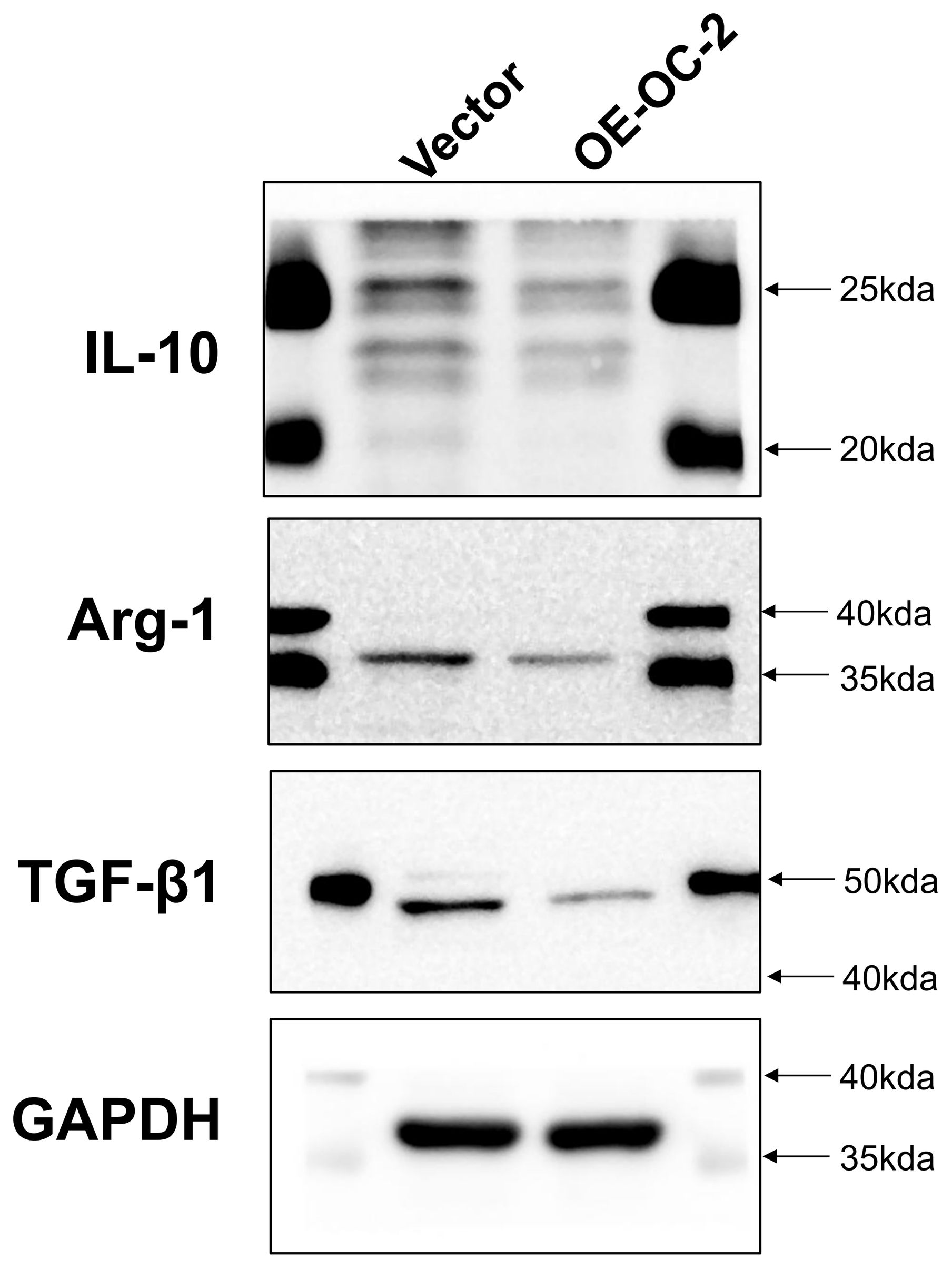
Vector
OE-OC-2
IL-10
25kda
20kda
Arg-1
40kda
35kda
TGF-β1
50kda
40kda
GAPDH
40kda
35kda

Fig6C

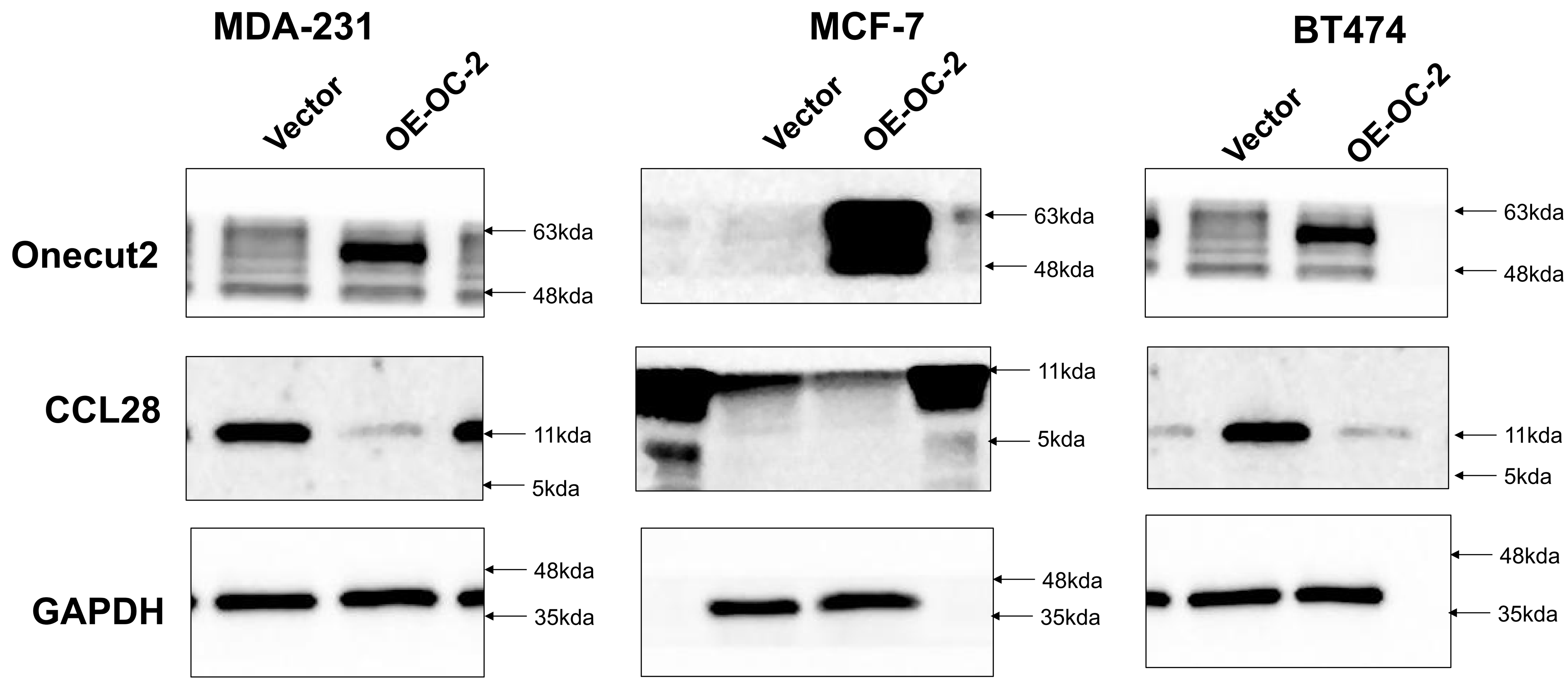

Fig6D

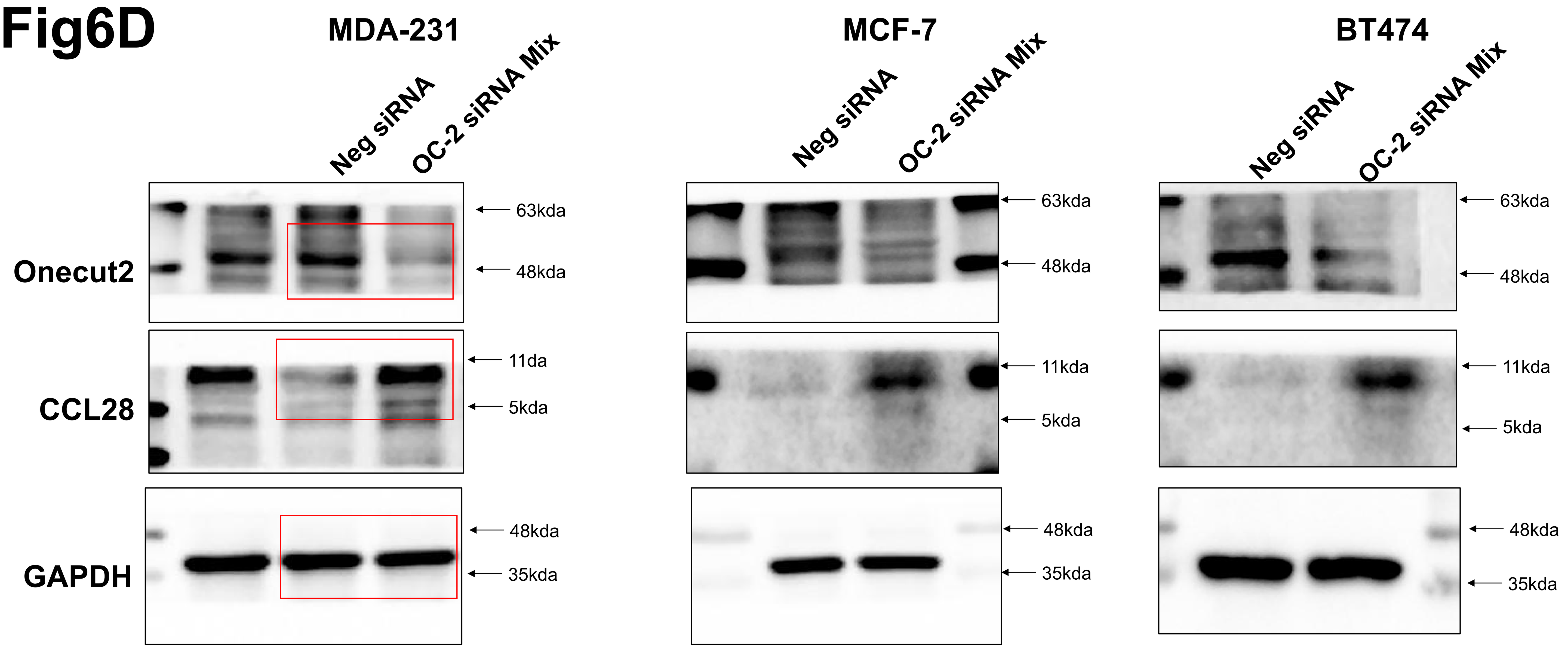

Fig6E

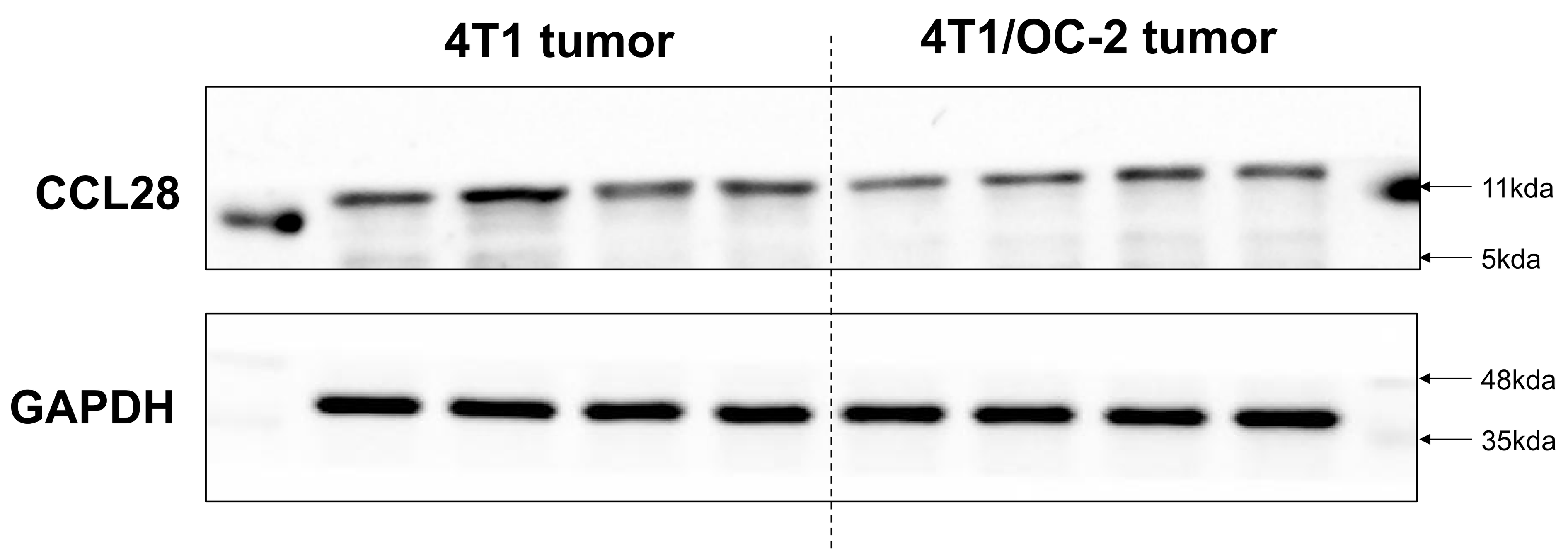

Fig6G

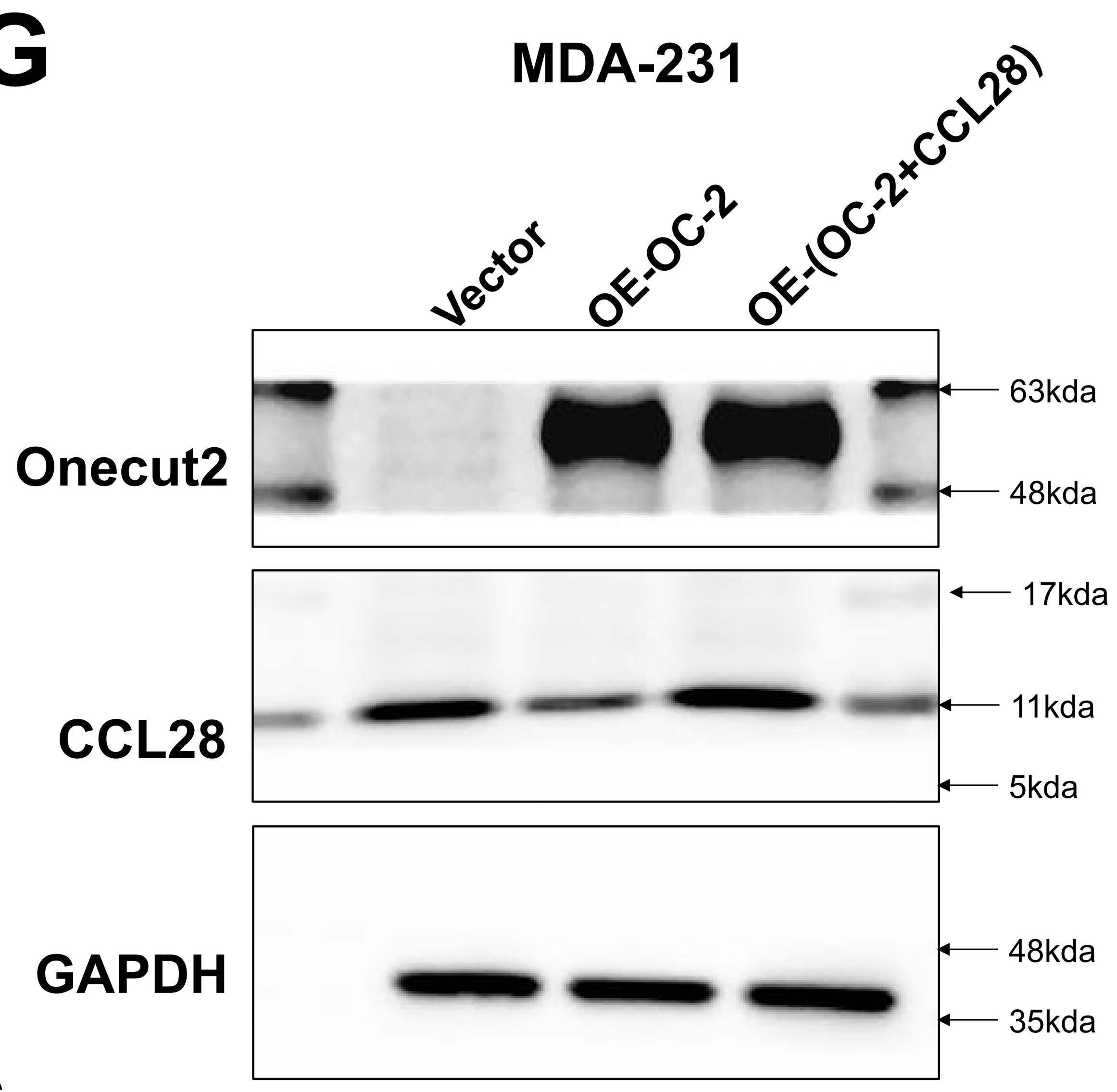

Fig6G

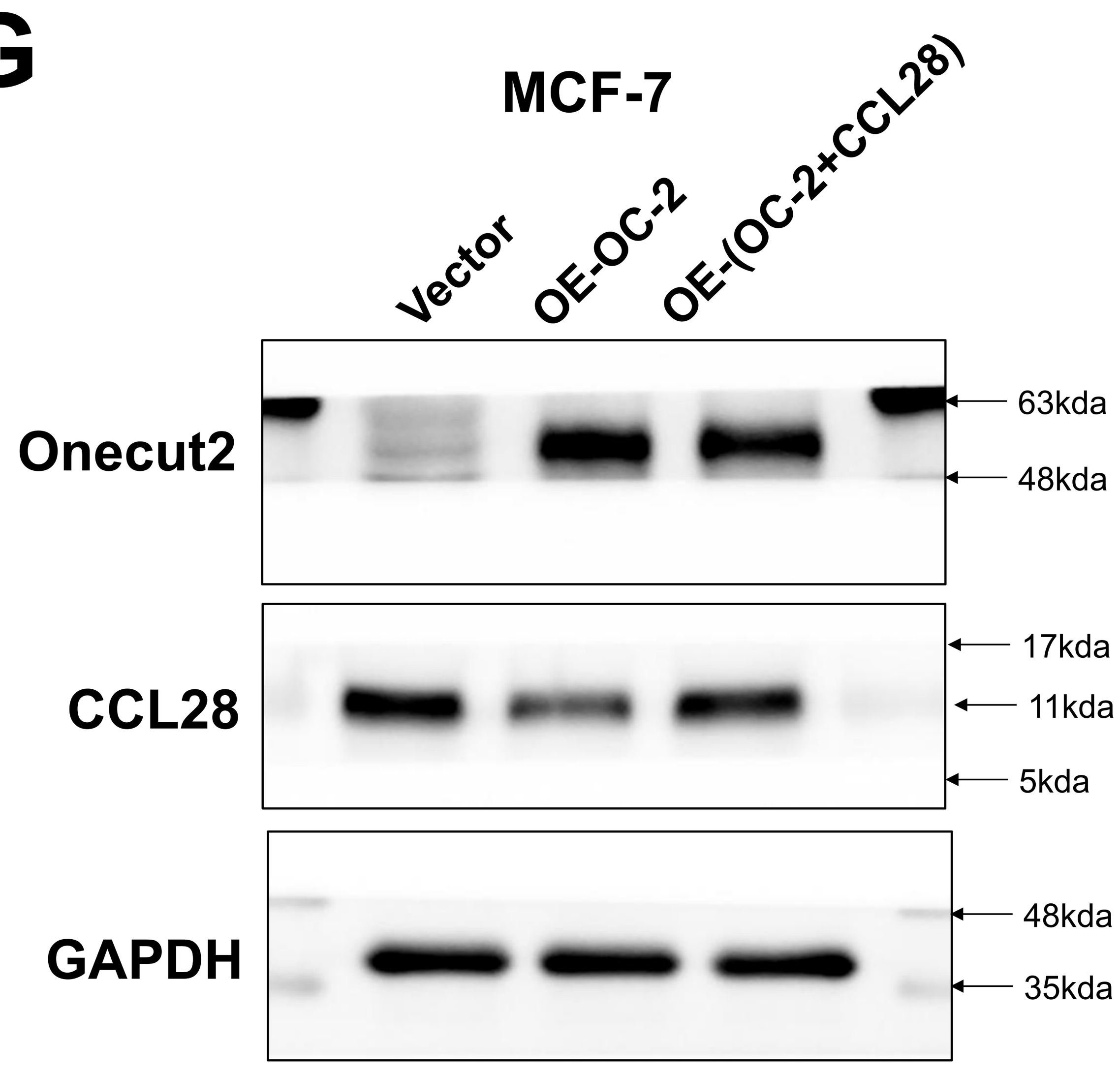

Fig6G

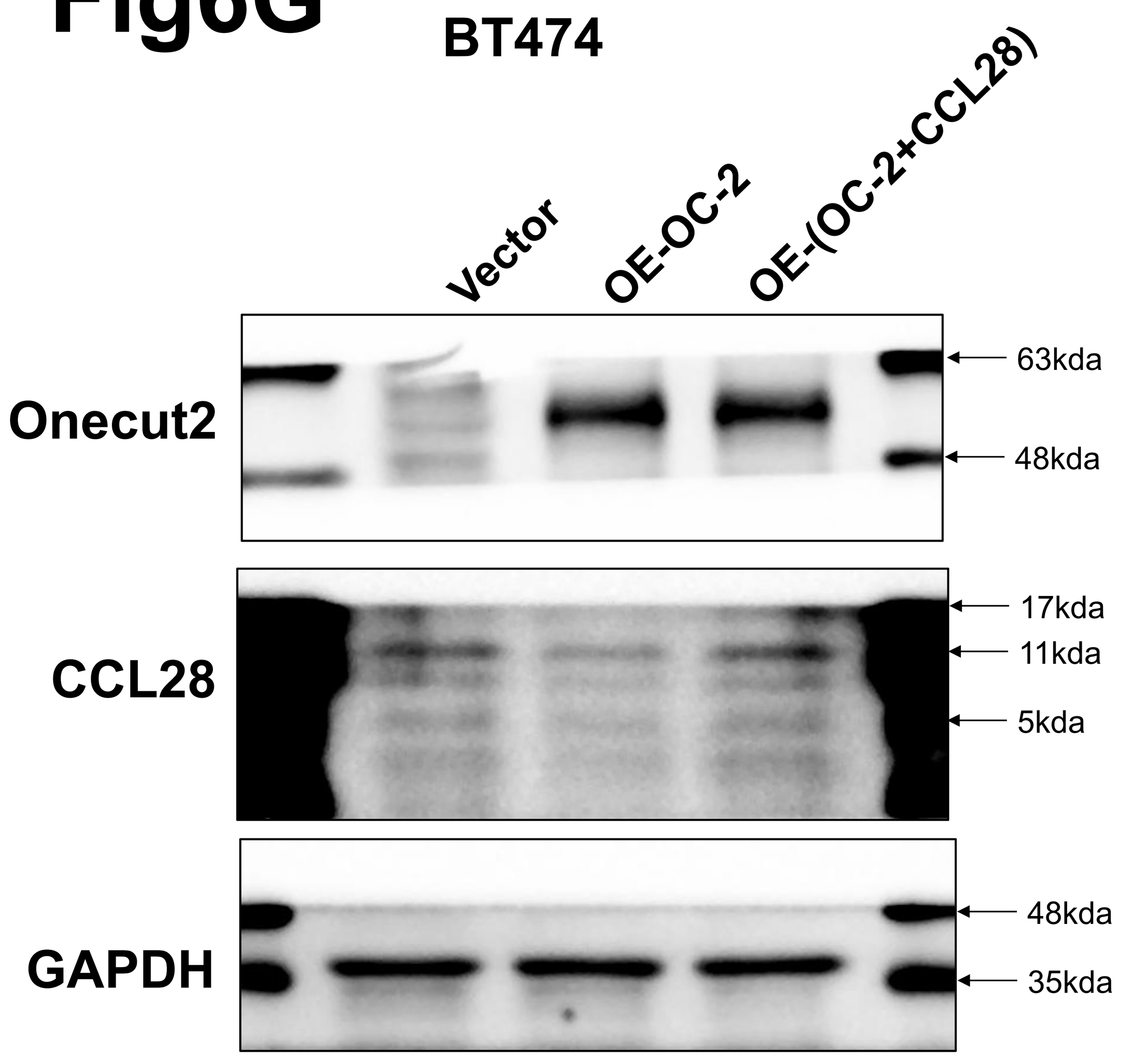

Fig6L

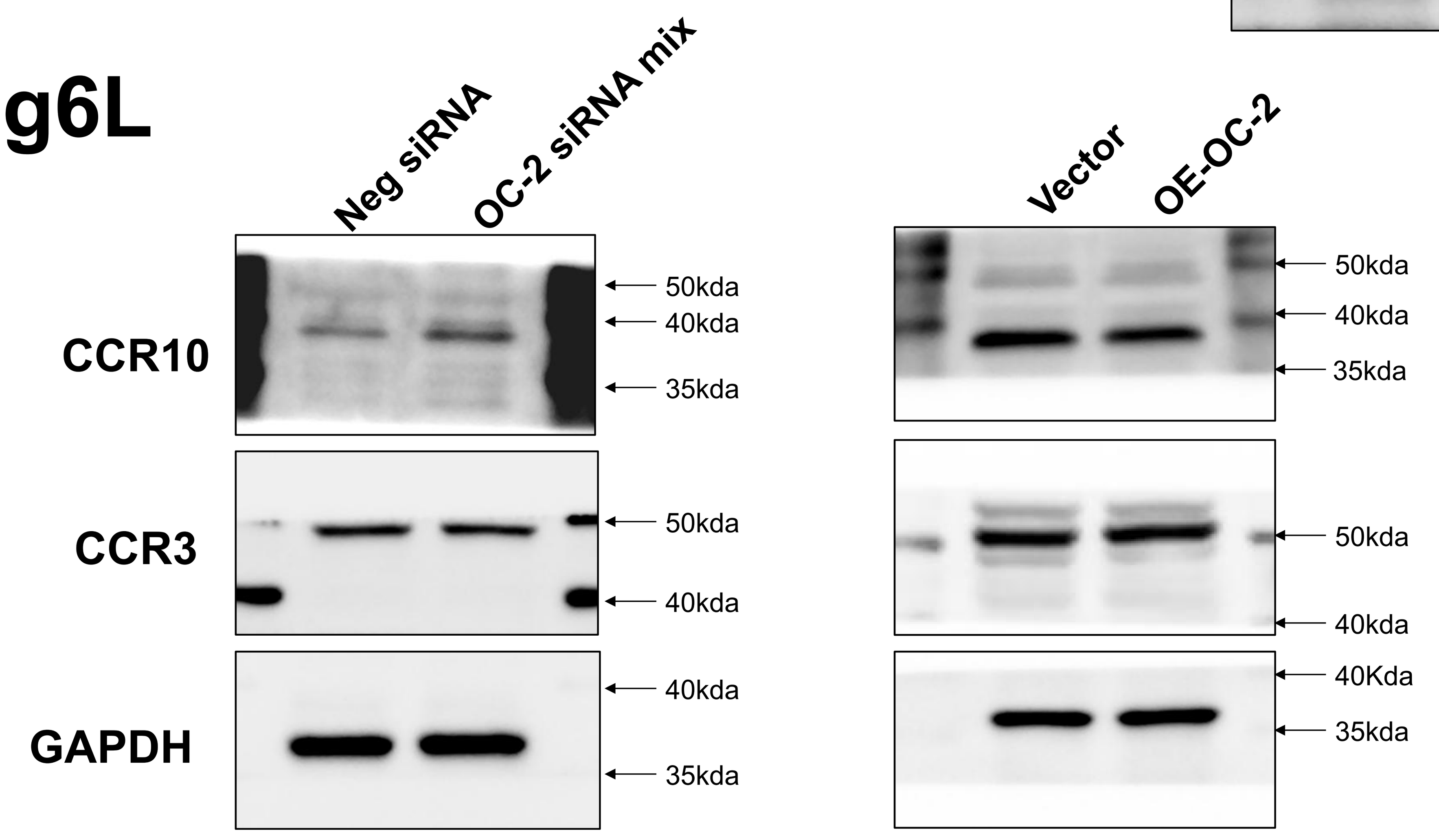

Fig. S4D

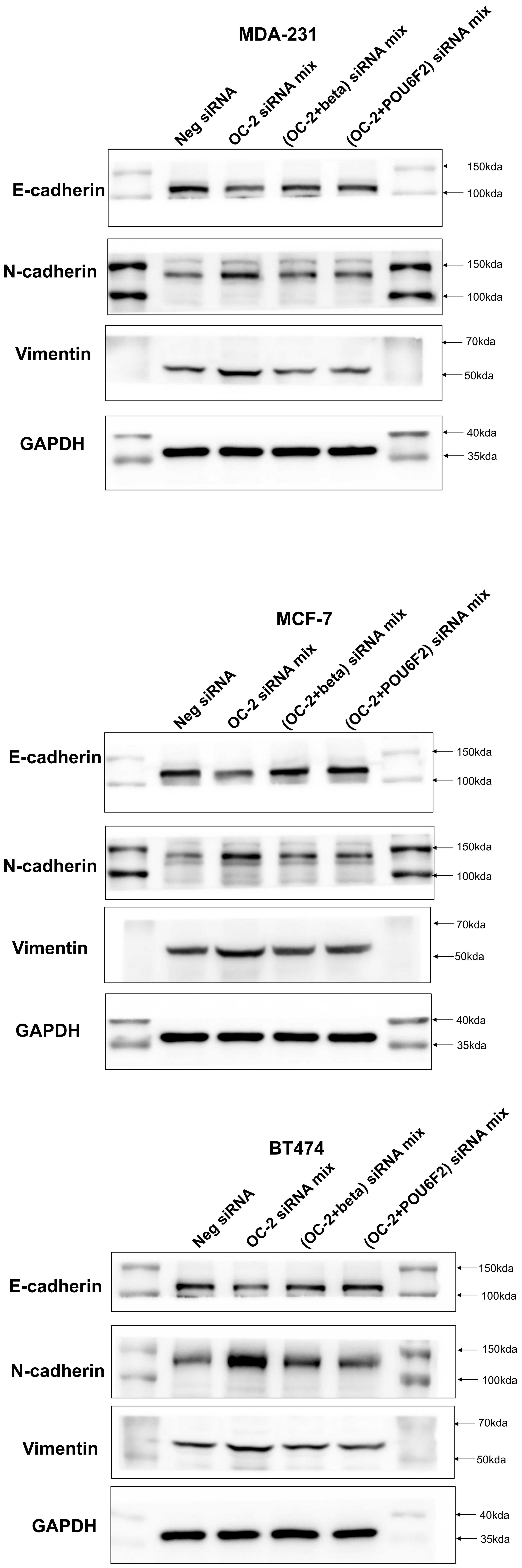

MDA-231
Neg siRNA
OC-2 siRNA mix
(OC-2+beta) siRNA mix
(OC-2+POU6F2) siRNA mix
E-cadherin
150kda
100kda
N-cadherin
150kda
100kda
Vimentin
70kda
50kda
GAPDH
40kda
35kda
MCF-7
Neg siRNA
OC-2 siRNA mix
(OC-2+beta) siRNA mix
(OC-2+POU6F2) siRNA mix
E-cadherin
150kda
100kda
N-cadherin
150kda
100kda
Vimentin
70kda
50kda
GAPDH
40kda
35kda
BT474
Neg siRNA
OC-2 siRNA mix
(OC-2+beta) siRNA mix
(OC-2+POU6F2) siRNA mix
E-cadherin
150kda
100kda
N-cadherin
150kda
100kda
Vimentin
70kda
50kda
GAPDH
40kda
35kda

FigS6

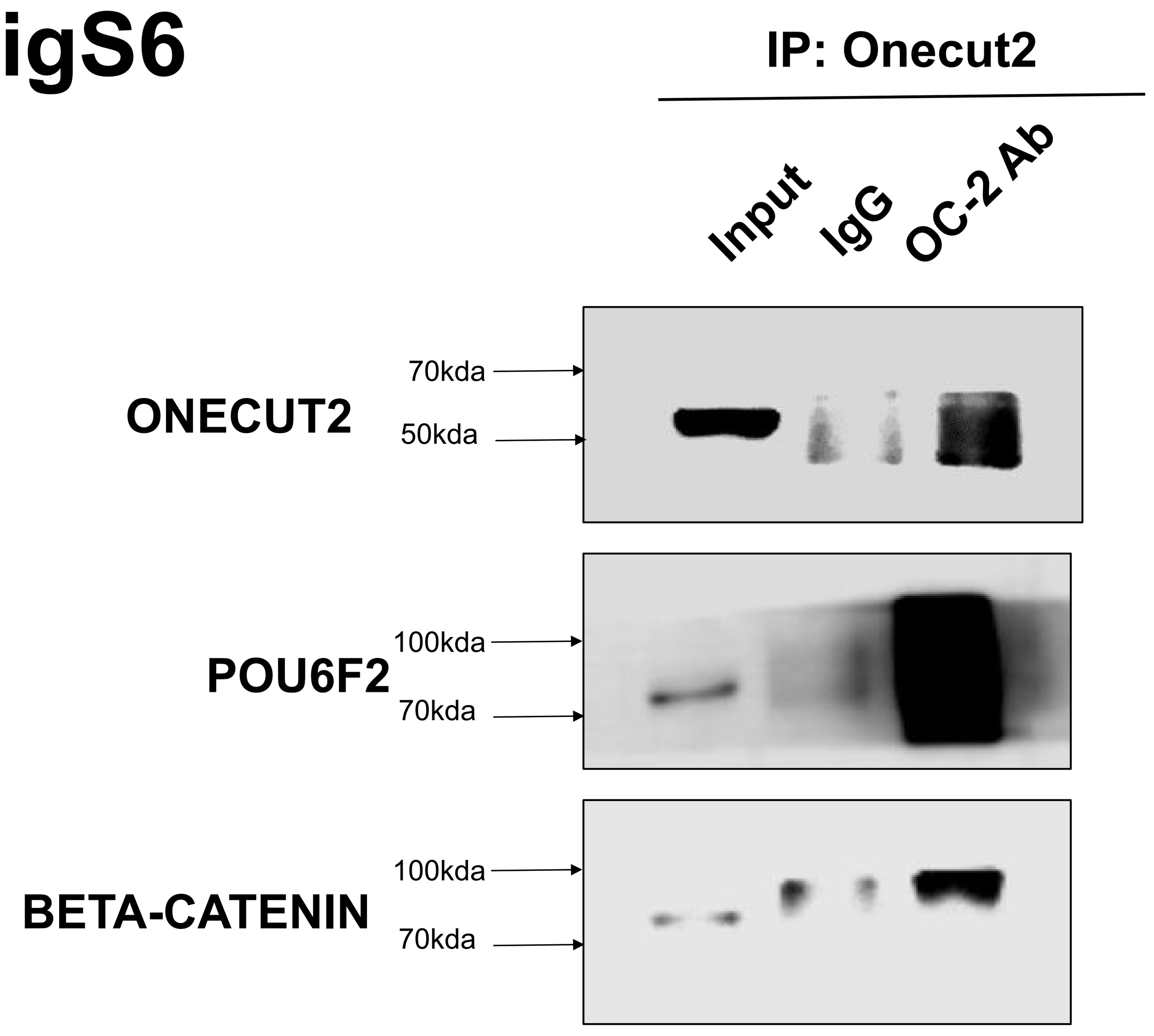

FigS9A

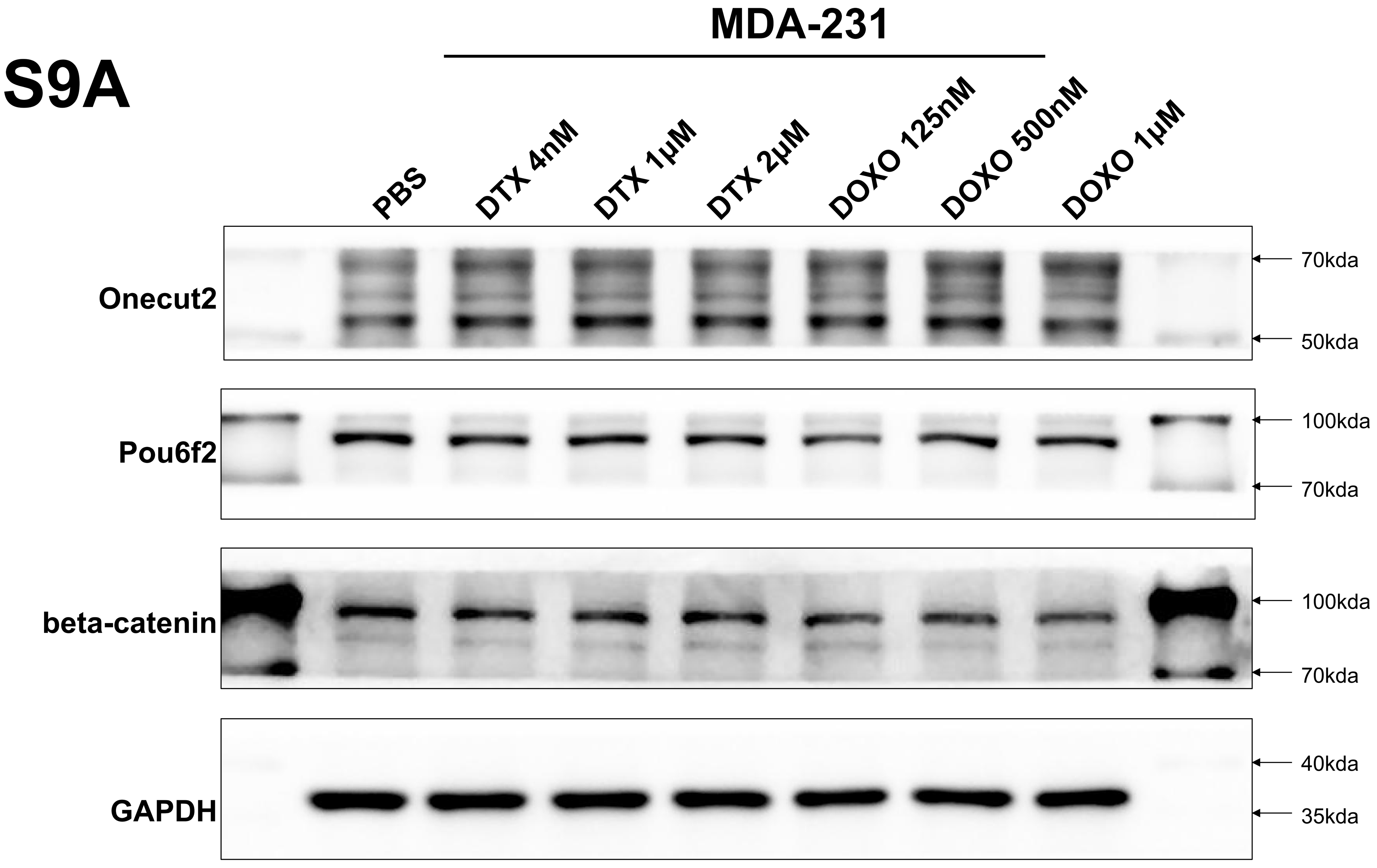

FigS9B

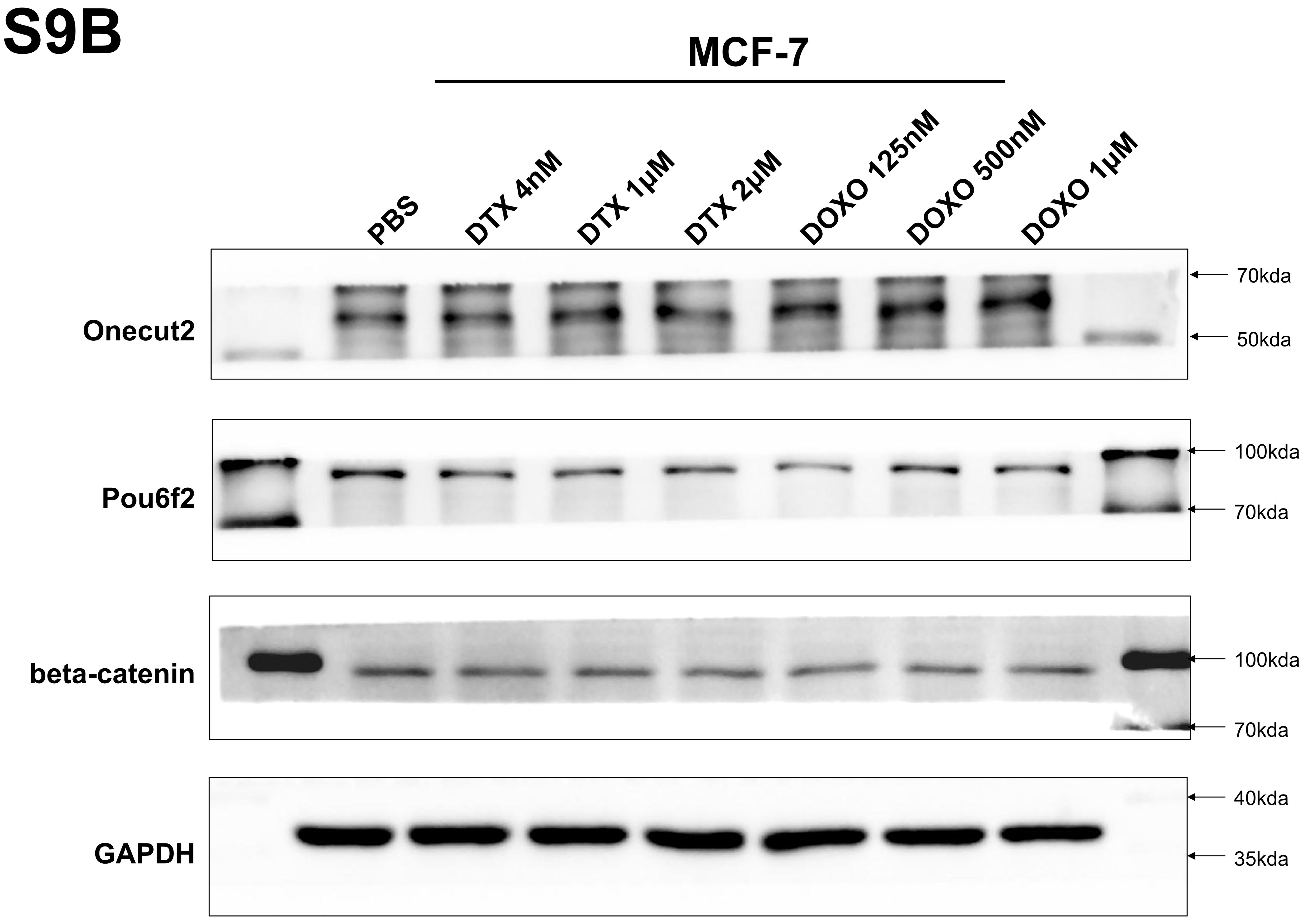

FigS9C

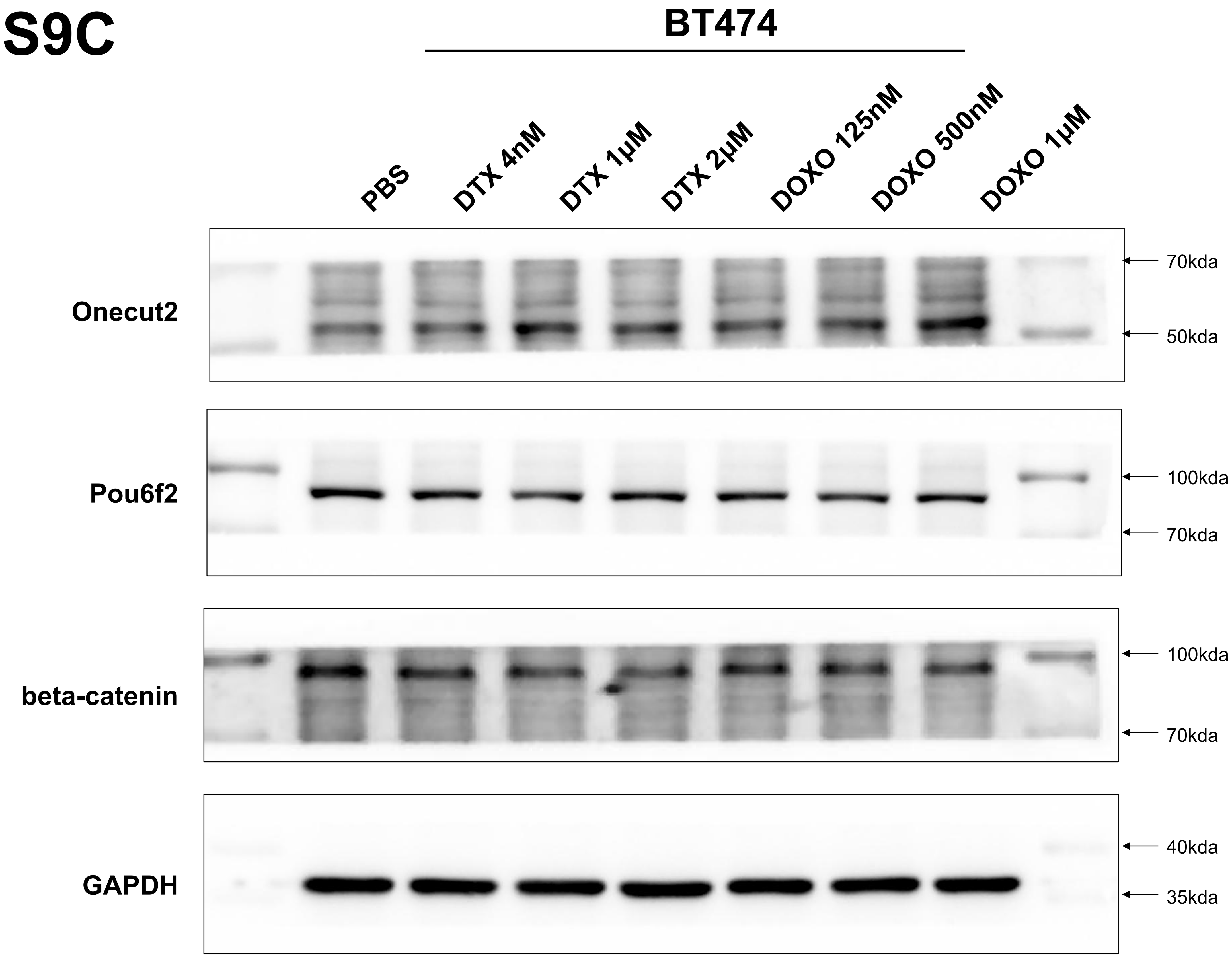

FigS14B

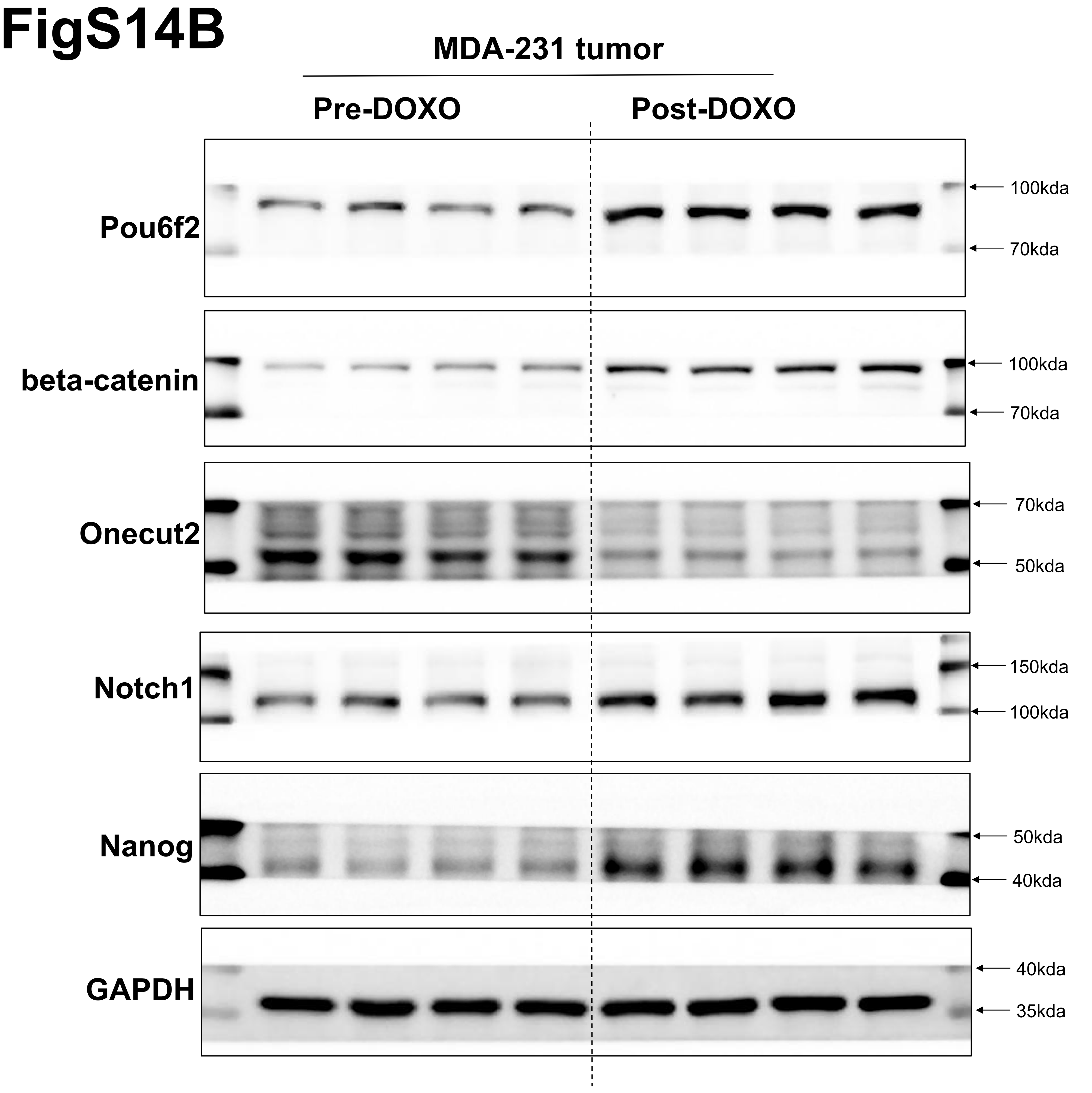

FigS14B

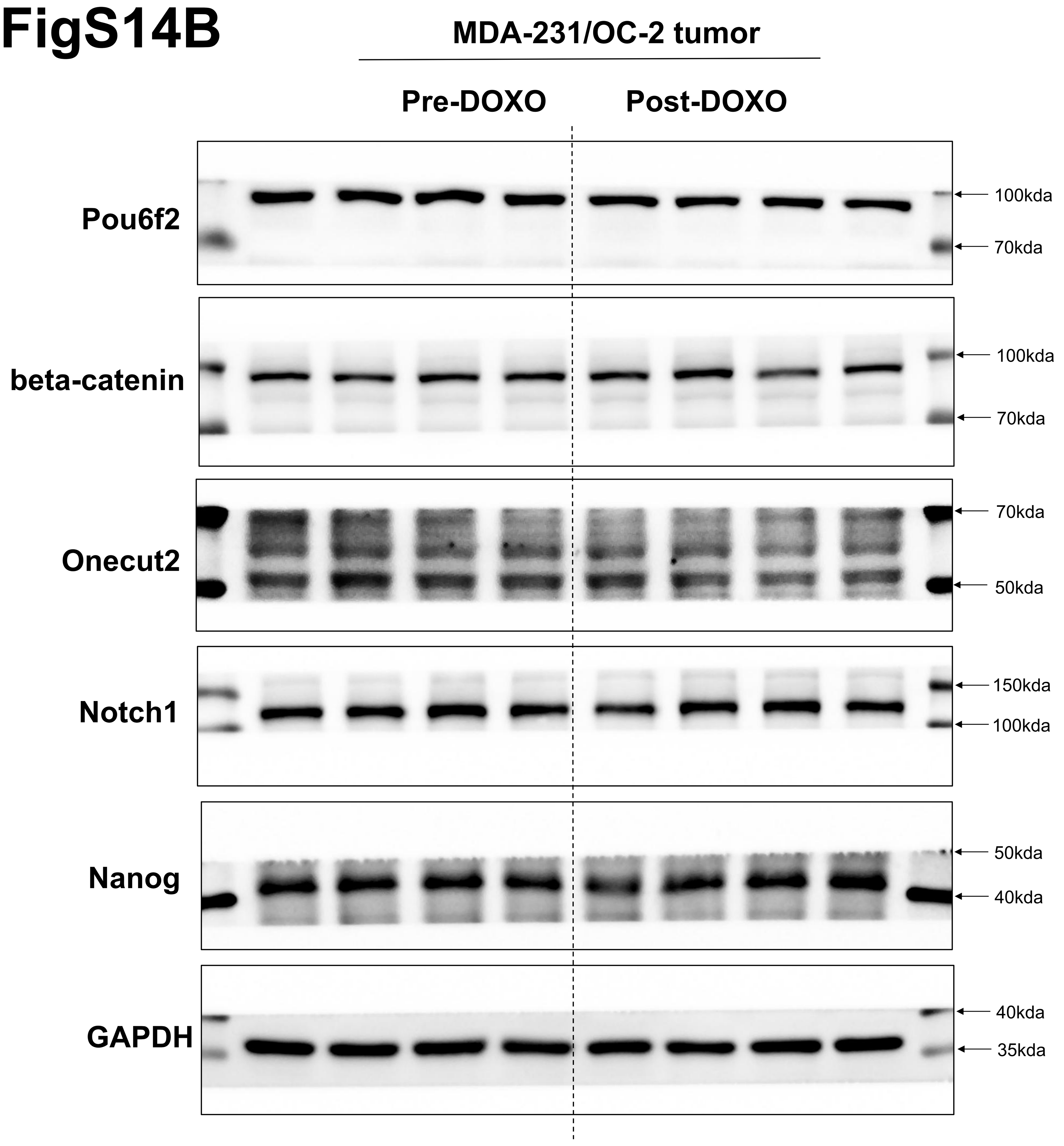

FigS15A

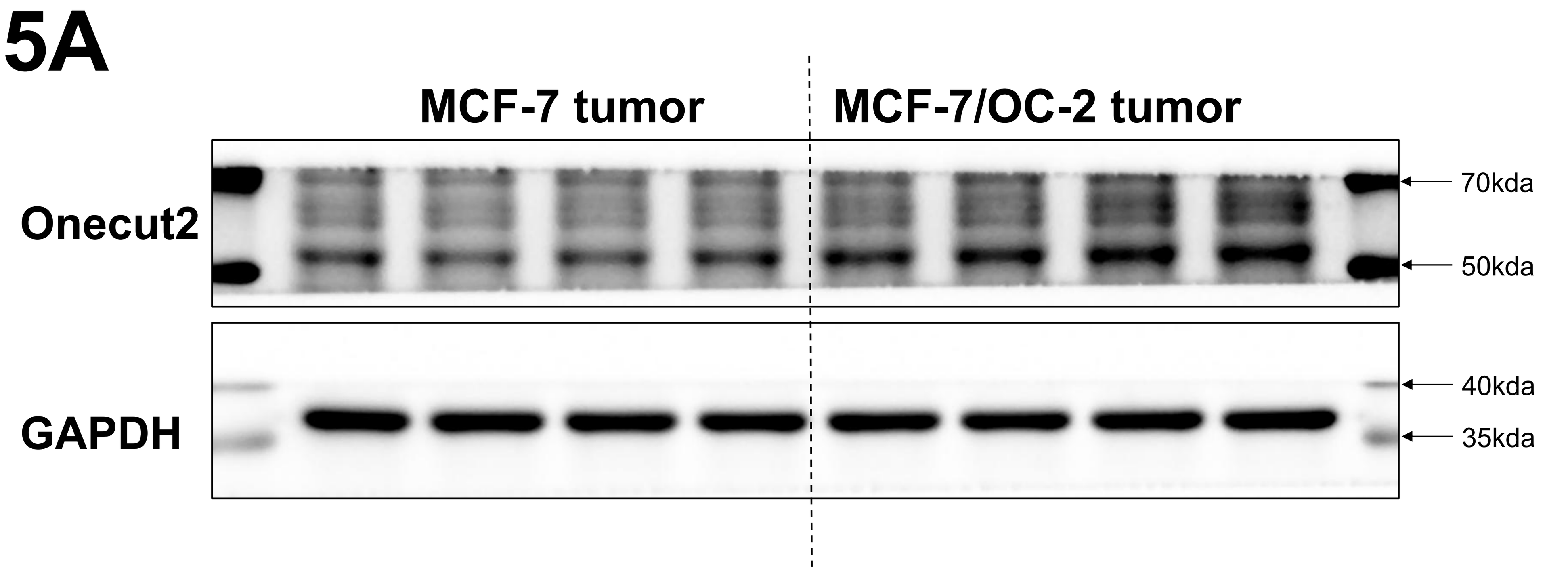

FigS15B

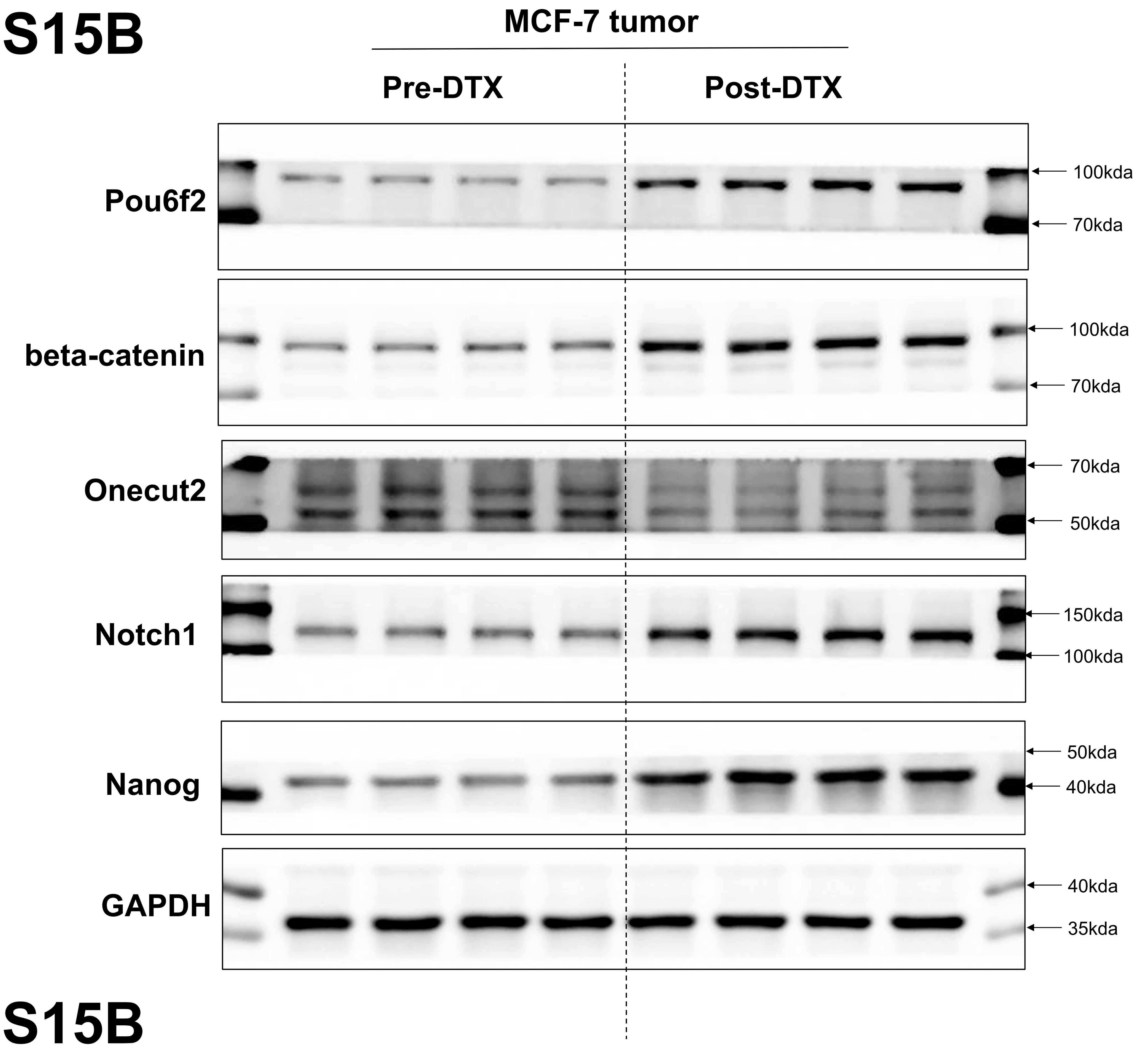

FigS15B

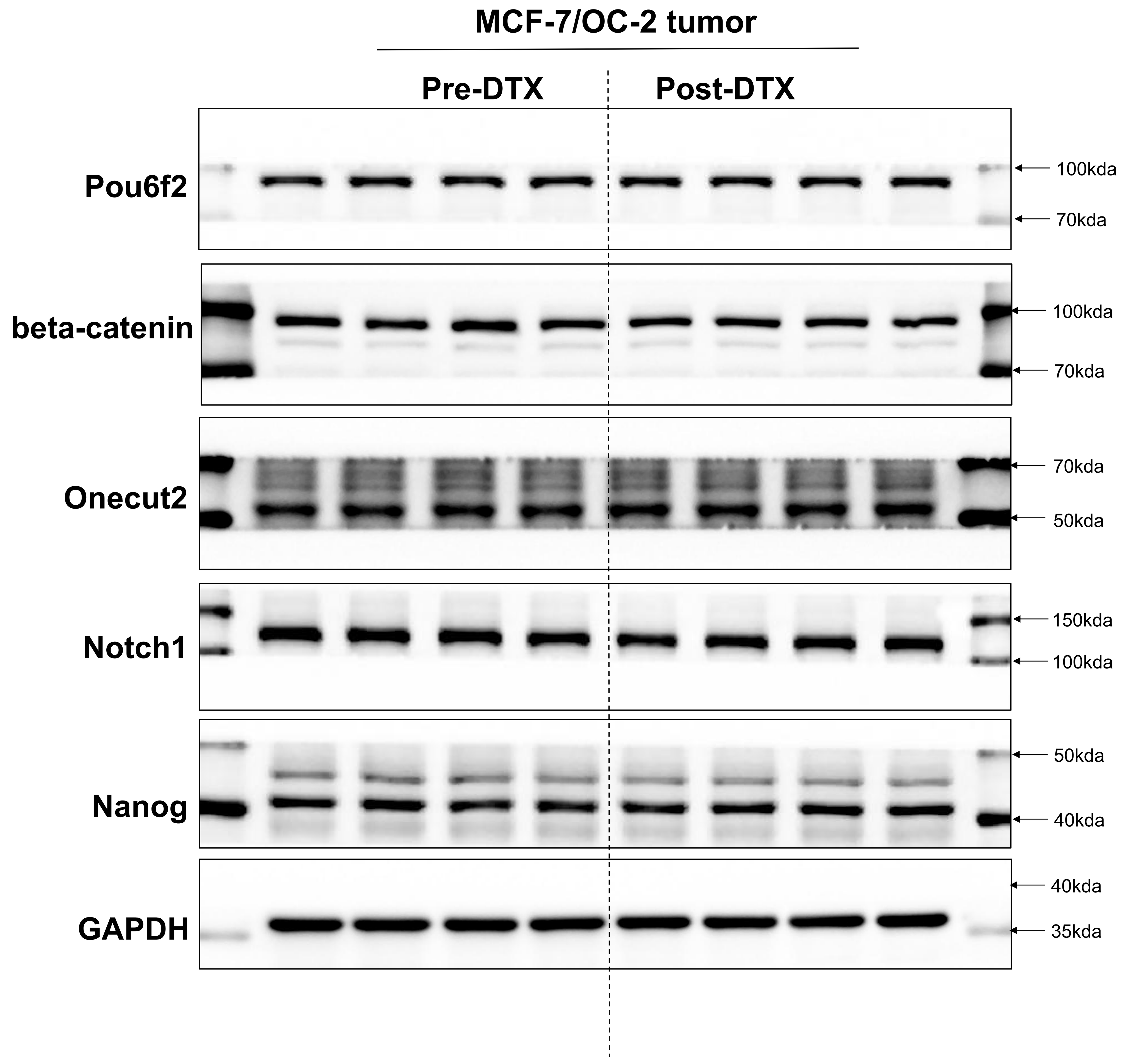

FigS16A

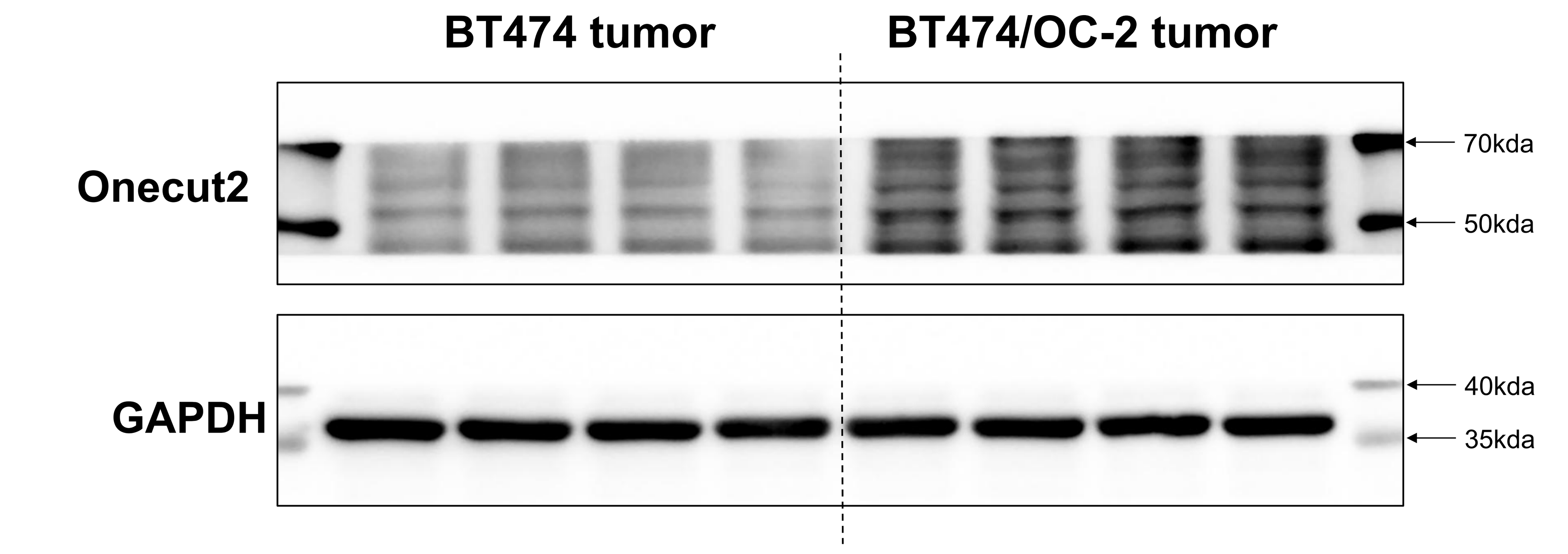

FigS16B

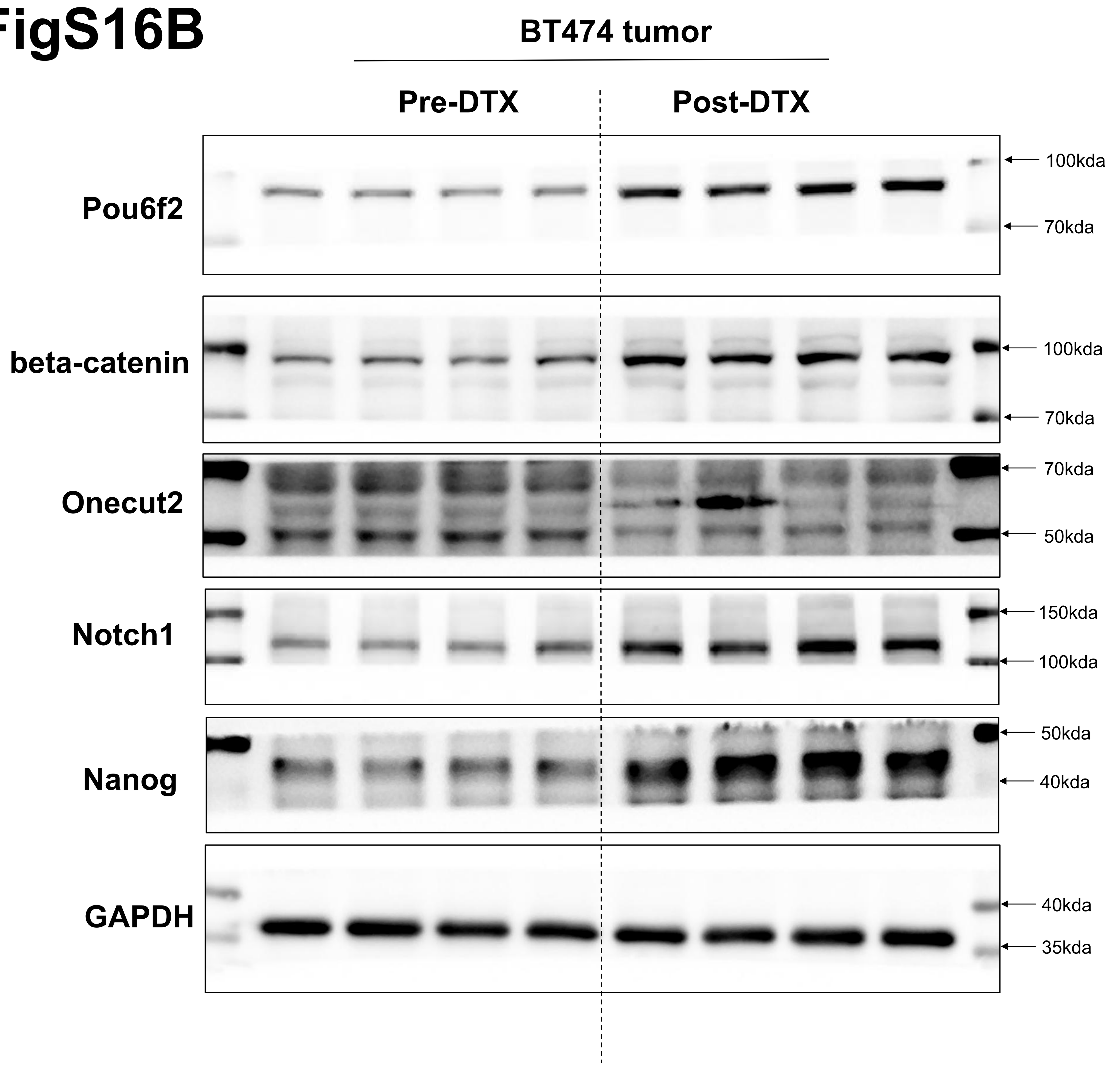

FigS16B

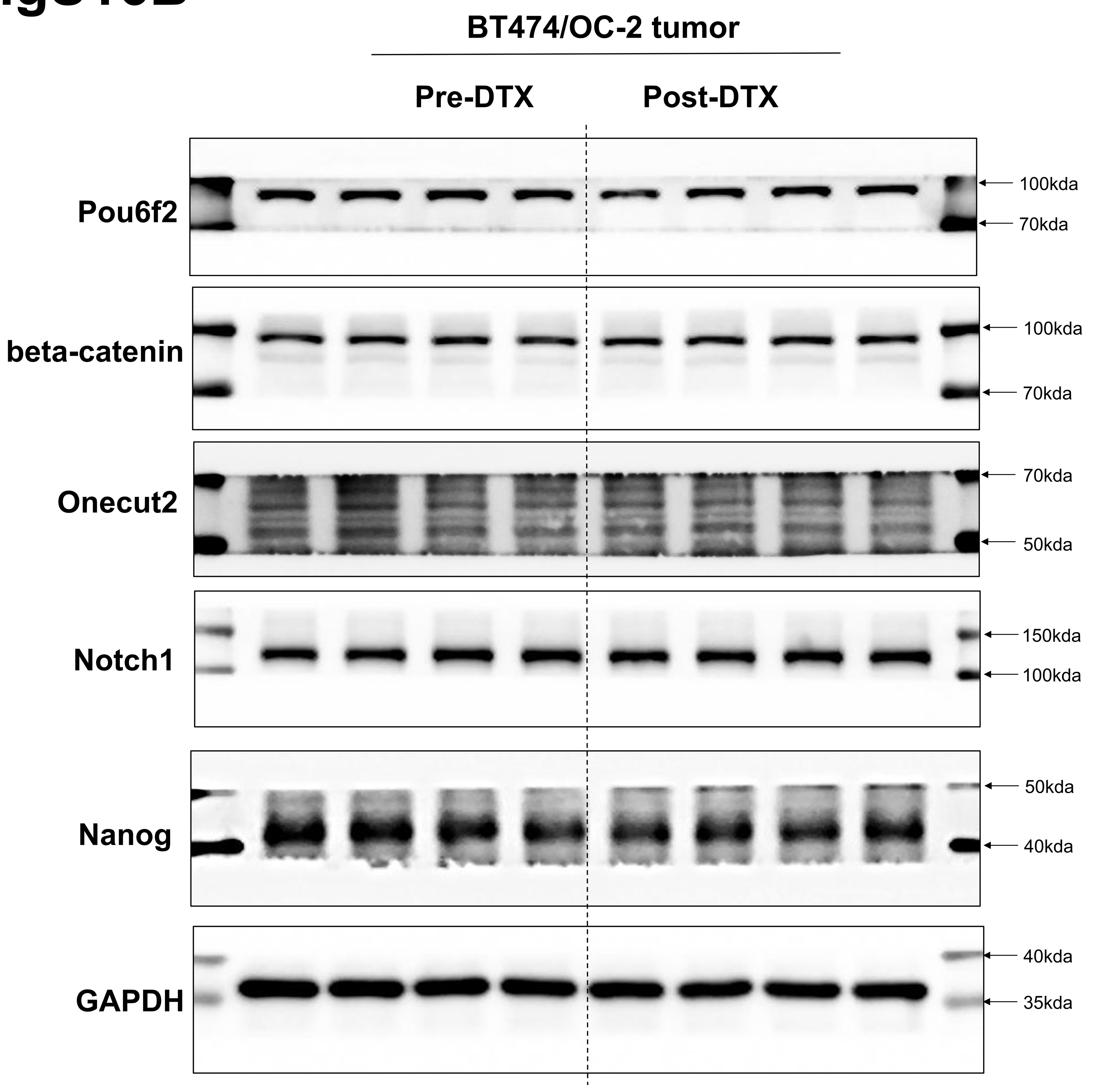

FigS20C

MCF-7

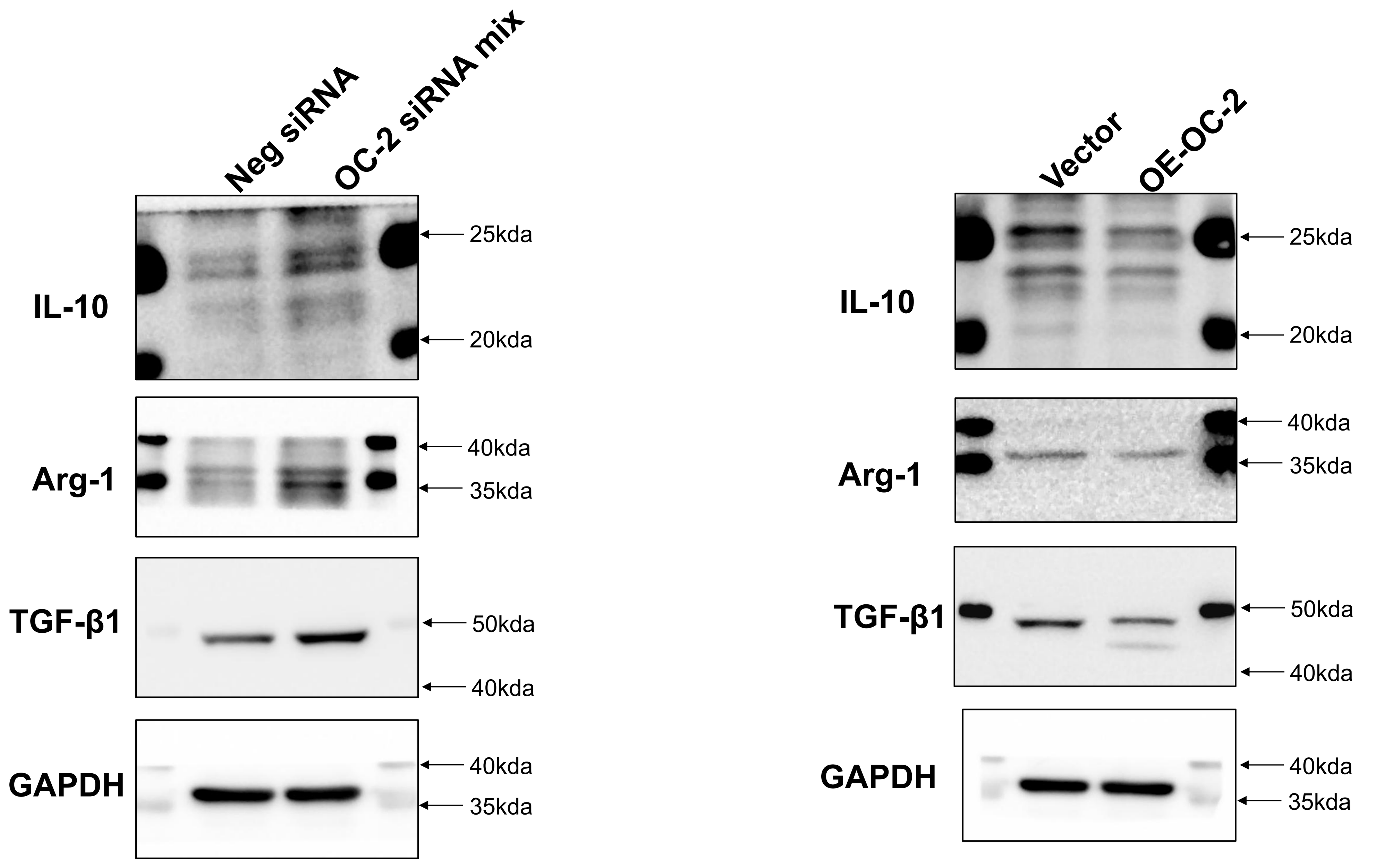

BT474

FigS20D

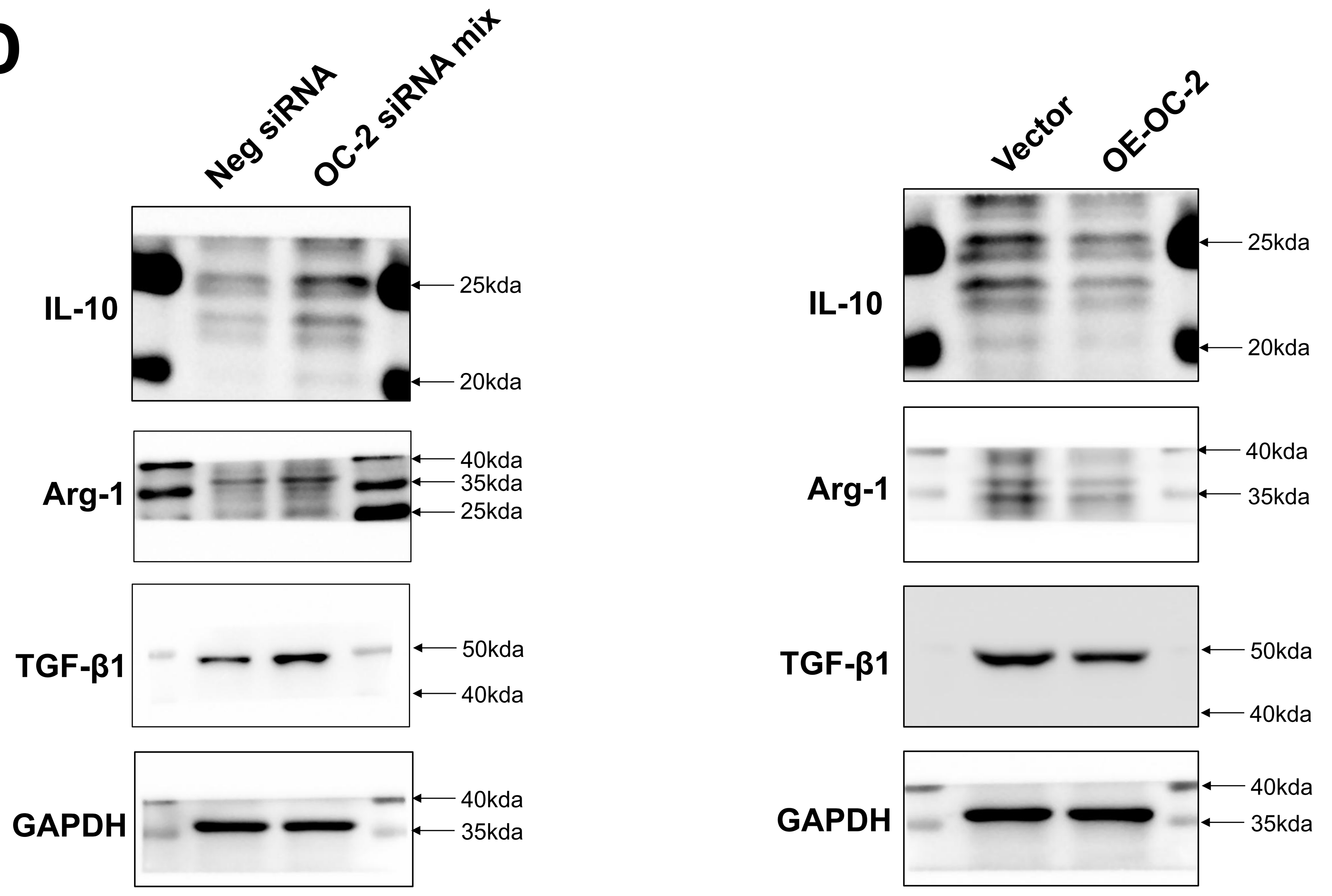

FigS25A

MCF-7

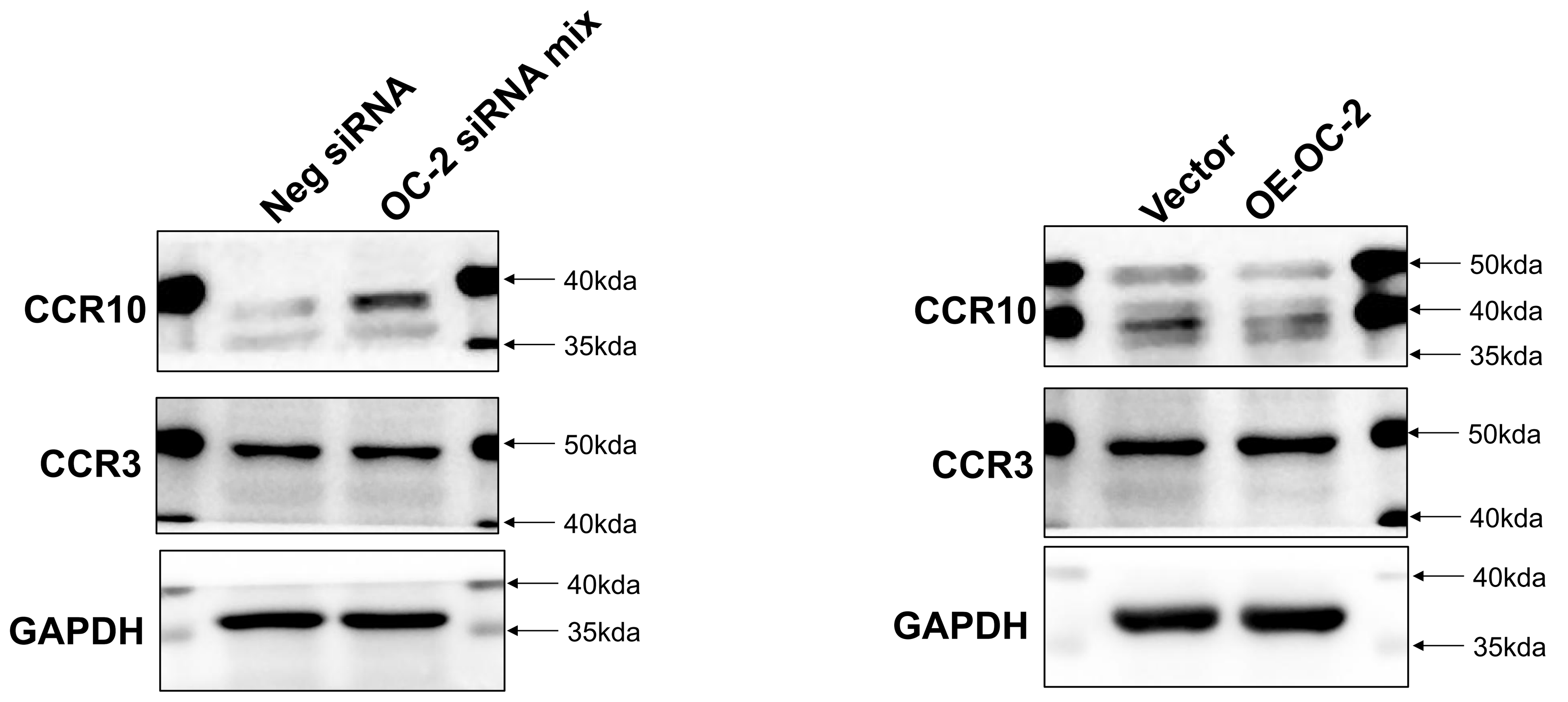

FigS25B

BT474

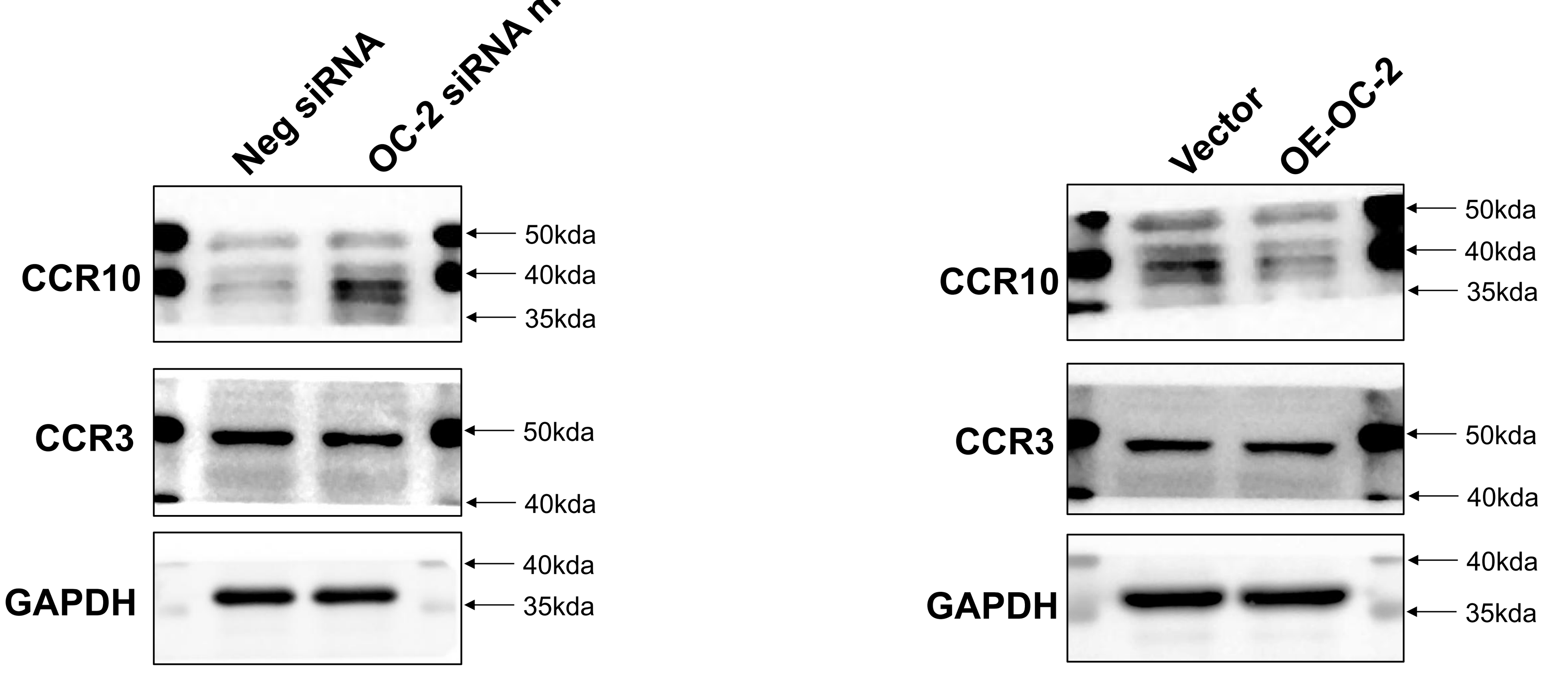

Supplement: Supplementary file 1 [file 41419_2026_8909_MOESM5_ESM.pdf]
