# Supplementary material for: Low expression of ONECUT2 governs the POU6F2-beta-catenin axis to modulate cancer stemness and drives the CCL28-dependent pathway for macrophage polarization in breast cancer
Source: Cell Death Dis. 2026 Jun 2;17(1):690. doi: 10.1038/s41419-026-08909-5 (PMC13443588; doi:10.1038/s41419-026-08909-5)
Supplement: Supplementary file 2 [file 41419_2026_8909_MOESM1_ESM.docx]

**Supplementary Information**

**Fig. S1** The transcriptional regulation of CTNNB1 by ONECUT2

**Fig. S2** ONECUT2 inhibition drives breast cancer stemness via the beta-catenin-dependent pathway

**Fig. S3** Prognostic significance and expression landscape of ONECUT2 in breast cancer subtypes

**Fig. S4** ONECUT2 regulates the EMT phenotype in breast cancer

**Fig. S5** Prognostic significance and expression landscape of POU6F2 in breast cancer subtypes

**Fig. S6** Co-IP validation of ONECUT2/POU6F2/BETA-CATENIN protein complex formation in MDA-231 cells

**Fig. S7** POU6F2 induces breast cancer stemness via beta-catenin activation

**Fig. S8** Dose-response profiling of chemotherapeutic sensitivity in POU6F2/ONECUT2-knockdown MCF-7 and BT474 cells

**Fig.S9** Direct effect of chemotherapy on breast cancer cell lines

**Fig.S10** Rescue of POU6F2-induced CSC traits by beta-catenin

**Fig. S11** Prognostic significance and expression landscape of BETA-CATENIN in breast cancer subtypes

**Fig.S12** Tumor growth in orthotopic 4T1 models with OC-2 overexpression or POU6F2 knockdown

**Fig. S13** Therapeutic effects and expression analysis in mouse models of breast cancer following chemotherapy

**Fig. S14** Characterization of DOXO-induced ONECUT2-POU6F2-beta-catenin axis in MDA-231 xenografts

**Fig. S15** Characterization of DTX-induced ONECUT2-POU6F2-beta-catenin axis in MCF-7 xenografts

**Fig. S16** Characterization of DTX-induced ONECUT2-POU6F2-beta-catenin axis in BT474 xenografts

**Fig. S17** Therapeutic effects of chemotherapy in mice bearing 4T1 or 4T1/shPOU6F2 tumors

**Fig. S18** Representative images from the FCM analysis of BALB/c tumor models

**Fig. S19** ONECUT2 expression in breast cancer cells influences M2 polarization of macrophages

**Fig. S20** Expression levels of cytokines released by macrophages that had been primed with conditioned medium from breast cancer cells under ONECUT2 inhibition or overexpression

**Fig. S21** Effects of breast cancer cell-educated THP-1 cells on the biological functions of tumor cells

**Fig. S22** ELISA assay for CCL28 secretion

**Fig. S23** CCL28 promotes M2 macrophage polarization in breast cancer

**Fig. S24** Gene expression changes in THP-1-derived macrophages treated with breast cancer cell conditioned medium

**Fig. S25** CCR3 and CCR10 expression levels in THP-1-derived macrophages treated with breast cancer cell conditioned medium

**Supplementary Materials and Methods**

**Table S1** Patient clinical parameters and their association with ONECUT2, POU6F2 and beta-catenin gene expression.

**Table S2** Primer sequences and antibodies.

**Table S3** The potential target genes of ONECUT2 using the GTRD.

**Fig. S1 The transcriptional regulation of CTNNB1 by ONECUT2** Dual-luciferase reporter assays were used to assess transcriptional regulation of CTNNB1 by ONECUT2 (ns: P＞0.05).

**Fig. S2 ONECUT2 inhibition drives breast cancer stemness via the beta-catenin-dependent pathway**

(A) Representative images of tumor spheres of MCF-7 cells transfected with a control siRNA, siONECUT2, or mixed siRNAs (ONECUT2 plus beta-catenin or ONECUT2 plus POU6F2). **(B, C)** MDA-231 cells (B) and BT474 (C) were transfected with a control siRNA, siONECUT2, or mixed siRNAs (ONECUT2 plus beta-catenin or ONECUT2 plus POU6F2), and collected for FCM to analyze the CD24^-^CD44^+^ cell population. **(D, E)** MDA-231 cells (D) and BT474 (E) were transfected with a control siRNA, siONECUT2, or mixed siRNAs (ONECUT2 plus beta-catenin or ONECUT2 plus POU6F2), and collected for sphere formation assay. Error bars represent mean ± SD of three independent experiments. *P<0.05, **P<0.01, ***P<0.001. Scale bar: 70 μm.

**Fig. S3 Prognostic significance and expression landscape of ONECUT2 in breast cancer subtypes**

(A) KM analysis reveals the prognostic correlation of ONECUT2 expression with OS in breast cancer patients (GEO: GSE9893, OS, n=155, p=0.0001). **(B)** KM analysis reveals the prognostic correlation of ONECUT2 expression with DFS and OS across breast cancer molecular subtypes in our cohort (n=99). **(C)** Expression profiling of ONECUT2 across breast cancer molecular subtypes in our cohort (n=99; p＞0.05). **(D)** Expression profiling of ONECUT2 across breast cancer molecular subtypes in TCGA database (n=942; TNBC vs HER2+: p=0.0050). **P<0.01.

**Fig. S4 ONECUT2 regulates the EMT phenotype in breast cancer**

**(A, B)** Transwell assay was used to assess the migration of MDA-231, MCF-7, and BT474 cells following ONECUT2 knockdown (A) or ONECUT2 overexpression (B). **(C)** Migration of MDA-231, MCF-7 and BT474 cells transfected with specific siRNAs was assessed by Transwell assay. **(D)** Western blots presenting the expression levels of indicated proteins in BC cells following transfection with specific siRNAs. *P<0.05, **P<0.01, ***P<0.001. Scale bar: 200 μm.

**Fig. S5 Prognostic significance and expression landscape of POU6F2 in breast cancer subtypes**

(A) KM analysis reveals the prognostic correlation of POU6F2 expression with DSS in breast cancer patients (GEO: GSE3494, n=234, disease-specific survival (DSS), p=0.0141). **(B)** KM analysis reveals the prognostic correlation of POU6F2 expression with DFS and OS across breast cancer molecular subtypes in our cohort (n=99). **(C)** Expression profiling of POU6F2 across breast cancer molecular subtypes in our cohort (n=99; p＞0.05). **(D)** Expression profiling of POU6F2 across breast cancer molecular subtypes in TCGA database (n=942; TNBC vs Luminal A: p=0.0033). **P<0.01.

**Fig. S6 Co-IP validation of ONECUT2/POU6F2/BETA-CATENIN protein complex formation in MDA-231 cells**

Co-IP assay using an anti-ONECUT2 antibody in MDA-231 cells was performed, followed by western blotting to verify the binding among ONECUT2, POU6F2 and BETA-CATENIN.

**Fig. S7 POU6F2 induces breast cancer stemness via beta-catenin activation**

**(A)** Representative images of tumor spheres of MCF-7 cells transfected with siPOU6F2 or a control siRNA. **(B)** Representative images of FCM analysis of MCF-7 cells transfected with siPOU6F2 or a control siRNA. **(C, D)** MDA-231 cells were transfected with siPOU6F2 or a control siRNA, and collected for sphere formation assay (C) and for FCM to analyze the CD24^-^CD44^+^ cell population (D). **(E, F)** BT474 cells were transfected with siPOU6F2 or a control siRNA, and collected for sphere formation assay (E) and for FCM to analyze the CD24^-^CD44^+^ cell population (F). Error bars represent mean ± SD of three independent experiments. *P<0.05, **P<0.01, ***P<0.001. Scale bar: 70 μm.

**Fig. S8 Dose-response profiling of chemotherapeutic sensitivity in POU6F2/ONECUT2-knockdown MCF-7 and BT474 cells**

**(A, B)** MCF-7 cells were transfected with siPOU6F2 or siONECUT2, followed by 48 h treatment with DTX (A, 0.01μM to 100μM) or DOXO (B, 0.001μM to 100μM). **(C, D)** BT474 cells were transfected with siPOU6F2 or siONECUT2, followed by 48 h treatment with DTX (C, 0.001μM to 100μM) or DOXO (D, 0.001μM to 100μM). Cell viability was measured by CCK-8 assay, with IC50 values calculated via nonlinear regression.

**Fig. S9 Direct effect of chemotherapy on breast cancer cell lines**

MDA231 (A), MCF-7 (B), and BT474 (C) cells were treated with DTX or DOXO at indicated concentrations or with PBS for 48 h. The protein levels of Onecut2, Pou6f2, and beta-catenin were detected by western blotting.

**Fig. S10 Rescue of POU6F2-induced CSC traits by beta-catenin**

MDA-231 (A), MCF-7 (B), and BT474 (C) cells were transfected with POU6F2-overexpression plasmid alone or in combination with beta-catenin siRNAs, then harvested for FCM to analyze the CD24^-^CD44^+^ cell population.

**Fig. S11 Prognostic significance and expression landscape of BETA-CATENIN in breast cancer subtypes**

(A) KM analysis reveals the prognostic correlation of BETA-CATENIN expression with OS in breast cancer patients (GEO: GSE20685, n=327, OS, p=0.0719). **(B)** KM analysis reveals the prognostic correlation of BETA-CATENIN expression with DFS and OS across breast cancer molecular subtypes in our cohort (n=99). **(C)** Expression profiling of BETA-CATENIN across breast cancer molecular subtypes in our cohort (n=99, p＞0.05). **(D)** Expression profiling of BETA-CATENIN across breast cancer molecular subtypes in TCGA database (n=942; TNBC vs HER2+: p=0.0002, TNBC vs Luminal B: p=0.0011). **P<0.01, ***P<0.001.

**Fig. S12 Tumor growth in orthotopic 4T1 models with OC-2 overexpression or POU6F2 knockdown**

**(A)** BALB/c mouse models were established by injection of 4T1 cells (n=4) or 4T1/OC-2 cells (n=4) into the mammary fat pad. Tumor onset and volume were measured. **(B)** BALB/c mouse models were established by injection of 4T1 cells (n=4) or 4T1/shPOU6F2 cells (n=4) into the mammary fat pad. Tumor onset and volume were measured.

**Fig. S13 Therapeutic effects and expression analysis in mouse models of breast cancer following chemotherapy**

**(A)** BALB/c tumor models were established by injection of 4T1 cells (n=4) or 4T1/OC-2 cells (n=4) into the 4# mammary fat pad. Tumor onset and volume were measured, with DTX or DOXO treatment marked by arrows. **(B)** Representative IHC images of ONECUT2, POU6F2, and BETA-CATENIN expression in MDA-231 NSG tumor tissues before and after DTX treatment.

**Fig. S14 Characterization of DOXO-induced ONECUT2-POU6F2-beta-catenin axis in MDA-231 xenografts**

**(A)** Xenograft tumors generated in NSG mice by injecting MDA-231 (n=4) and MDA-231/OC-2 (n=4) cells. Tumor volume was measured, with DOXO treatment marked by arrows (red arrows for MDA-231/OC-2 tumors and black arrows for MDA-231 tumors, indicating treatment with DOXO). **(B)** Western blots of indicated proteins in tumor tissues from MDA-231 mice (n=4, Left panel) and MDA-231/OC-2 mice (n=4, Right panel) collected before and after DOXO treatment.

**Fig. S15 Characterization of DTX-induced ONECUT2-POU6F2-beta-catenin axis in MCF-7 xenografts**

**(A)** Xenograft tumors generated in NSG mice by injecting MCF-7 (n=4) and MCF-7/OC-2 (n=4) cells. Tumor volume was measured, with DTX treatments marked by arrows (Left panel). The protein level of ONECUT2 in NSG tumor models was measured (Right panel).**(B)** Western blots of indicated proteins in tumor tissues from MCF-7 mice (n=4, Left panel) and MCF-7/OC-2 mice (n=4, Right panel) collected before and after DTX treatment.

**Fig. S16 Characterization of DTX-induced ONECUT2-POU6F2-beta-catenin axis in BT474 xenografts**

**(A)** Xenograft tumors generated in NSG mice by injecting BT474 (n=4) and BT474/OC-2 (n=4) cells. Tumor volume was measured, with DTX treatments marked by arrows (Left panel). The protein level of ONECUT2 in NSG tumor models was measured (Right panel).**(B)** Western blots of indicated proteins in tumor tissues from BT474 mice (n=4, Left panel) and BT474/OC-2 mice (n=4, Right panel) collected before and after DTX treatment.

**Fig. S17 Therapeutic effects of chemotherapy in mice bearing 4T1 or 4T1/shPOU6F2 tumors**

BALB/c mouse models were established by injection of 4T1 cells (n=4) or 4T1/shPOU6F2 cells (n=4) into the mammary fat pad. Tumor onset and volume were measured, with DTX (A) or DOXO (B) treatments marked by arrows (red arrows for 4T1/shPOU6F2 tumors and blue arrows for 4T1 tumors, indicating treatment with DTX or DOXO).

**Fig. S18 Representative images from the FCM analysis of BALB/c tumor models**

The percentages of immunocytes (M2 macrophages, macrophages, CD3^+^T cells, CD4^+^T cells, CD8^+^T cells, NK cells, B cells, and Tregs) in the spleen tissues from 4T1/OC-2 (n=4) or 4T1 (n=4) mice were elevated by FCM analysis.

**Fig. S19 ONECUT2 expression in breast cancer cells influences M2 polarization of macrophages**

**(A)** Representative images of FCM analysis in THP-1-derived macrophages. THP-1 cells were co-cultured with MDA-231 conditioned medium, and the CD163^+^CD68^+^ population was assessed by FCM analysis. **(B)** THP-1 cells were co-cultured with MCF-7 conditioned medium, and the CD163^+^CD68^+^ population was assessed by FCM analysis. **(C)** THP-1 cells were co-cultured with BT474 conditioned medium, and the CD163^+^CD68^+^ population was assessed by FCM analysis. **(D, E)** RT-qPCR of CD163 and CD206 in THP-1-derived macrophages treated with conditioned medium from MCF-7 (D) and BT474 (E) with ONECUT2 knockdown (Left panel) or overexpression (Right panel).**P<0.01, ***P<0.001.

**Fig. S20 Expression levels of cytokines released by macrophages that had been primed with conditioned medium from breast cancer cells under ONECUT2 inhibition or overexpression**

**(A, B)** RT-qPCR of IL-10, ARG1 and TGFB1 in THP-1-derived macrophages that had been primed with CM from MCF-7 (A) and BT474 (B) cells under ONECUT2 inhibition (Left panel) and overexpression (Right panel), compared to their respective control groups. **(C, D)** Western blots of IL-10, Arg-1 and TGF-β1 in THP-1-derived macrophages that had been primed with CM from MCF-7 (C) and BT474 (D) cells under ONECUT2 inhibition (Left panel) and overexpression (Right panel), compared to their respective control groups. **(E, F)** ELISA analysis of IL-10, Arg-1 and TGF-β1 in THP-1-derived macrophages that had been primed with CM from MCF-7 and BT474 cells under ONECUT2 inhibition (E) and overexpression (F), compared to their respective control groups. *P<0.05, **P<0.01, ***P<0.001.

**Fig. S21 Effects of breast cancer cell-educated THP-1 cells on the biological functions of tumor cells**

**(A, B)** FCM analysis of apoptosis in breast cancer cells co-cultured with CM from THP-1 cells primed with ONECUT2-downregulated (A) or ONECUT2-overexpressing (B) breast cancer cells, showing representative results for breast cancer cell apoptosis. **(C)** Migration of breast cancer cells was assessed by Transwell assay following exposure to CM from THP-1 cells that had been primed with ONECUT2-downregulated (Left panel) or ONECUT2-overexpressing (Right panel) breast cancer cells. **P<0.01, ***P<0.001, Scale bar: 200 μm.

**Fig. S22 ELISA assay for CCL28 secretion**

ELISA was performed to test the secretion of CCL28 by MDA-231 cells transfected with ONECUT2 siRNAs (Left panel) or an overexpression plasmid (Right panel). Error bars represent mean ± SD of three independent experiments. ***P<0.001.

**Fig. S23 CCL28 promotes M2 macrophage polarization in breast cancer**

**(A)** Representative images of FCM analysis in THP-1-derived macrophages. THP-1 cells were co-cultured with MDA-231 conditioned medium, and the CD163^+^CD68^+^ population was assessed by FCM analysis. **(B, C)** THP-1 cells were co-cultured with MCF-7 (B) and BT474 (C) conditioned medium, and the CD163^+^CD68^+^ population was assessed by FCM analysis. Error bars represent mean ± SD of three independent experiments. **P<0.01, ***P<0.001.

**Fig. S24 Gene expression changes in THP-1-derived macrophages treated with breast cancer cell conditioned medium**

RT-qPCR analysis of the mRNA levels of CD163 and CD206 in THP-1 cells that had been primed with CM from MDA-231 **(A)**, MCF-7 **(B)** and BT474 **(C)**. Error bars represent mean ± SD of three independent experiments. *P<0.05, **P<0.01, ***P<0.001.

**Fig. S25 CCR3 and CCR10 expression levels in THP-1-derived macrophages treated with breast cancer cell conditioned medium**

**(A, B)** RT-qPCR (Left panel) and western blotting (Right panel) analysis for the expression levels of CCR3 and CCR10 in THP-1 cells that had been primed with CM from MCF-7 (A) and BT474 cells (B). **(C-E)** RT-qPCR analysis of the mRNA levels of CCR3 and CCR10 in THP-1 cells that had been primed with CM from MDA-231 (C), MCF-7 (D), and BT474 cells (E). Error bars represent mean ± SD of three independent experiments. *P<0.05, **P<0.01, ***P<0.001.

**Supplementary Materials and Methods**

**Animals**

1. **4T1 Models in BALB/c Mice:**

4T1 cells and their stably overexpressing ONECUT2 (4T1/OC-2) or stably knocking down POU6F2 (4T1/shPOU6F2) cell lines were cultured in RPMI-1640 medium with 10% FBS. Cells were routinely passaged at 80-90% confluence and routinely screened for mycoplasma contamination.

1) **Fixed Endpoint Model**

Six-week-old female BALB/c mice were randomly assigned to groups (n=4) and received a blinded, orthotopic injection of 1×10^6^ 4T1 cells, 4T1/OC-2 cells or 4T1/shPOU6F2 cells into the #4 mammary fat pad. The tumor volume was measured using calipers. Tumors were harvested 20 days after injection and collected for subsequent experiments.

2) **Chemotherapy Response Model**

1×10^6^ 4T1 cells, 4T1/OC-2 cells and 4T1/shPOU6F2 cells were injected into #4 mammary fat pad of BALB/c mice. Once the tumor volume reached ~200 mm^3^, mice were randomly divided into two cohorts: a pre-treatment cohort, which was sacrificed immediately (designated as the Pre-DTX/DOXO group, n=4 per group), and a post-treatment cohort (designated as the Post-DTX/DOXO group, n=4 per group), which received DTX (15 mg/kg) or DOXO (2 mg/kg) IP once a week for three weeks and sacrificed three days after the final chemodrug treatment. Tumor tissues were collected for subsequent experiments.

2. **MDA-231 Models in NSG Mice:**

MDA-231 cells and the stable ONECUT2-overexpressing cells (MDA-231/OC-2) were cultured in RPMI-1640 medium with 10% FBS. Cells were routinely passaged at 80-90% confluence and routinely screened for mycoplasma contamination.

**Chemotherapy Response Model**

2×10^5^ MDA-231 or MDA-231/OC-2 cells were injected into #4 mammary fat pad of six-week-old female NSG mice in a blinded and random manner. At ~300 mm³ tumor volume, mice were split into two groups: a pre-treatment cohort, which was sacrificed immediately (designated as the Pre-DTX group, n=4 for MDA-231 mice, n=3 for MDA-231/OC-2 mice; or Pre-DOXO group, n=4 per group), and a post-treatment cohort (designated as the Post-DTX group, n=4 for MDA-231 mice, n=3 for MDA-231/OC-2 mice; or Post-DOXO group, n=4 per group), which received DTX (15 mg/kg) or DOXO (2 mg/kg) IP once a week for three weeks and sacrificed three days after the final chemodrug treatment. Tumor tissues were collected for subsequent experiments.

3. **MCF-7 Models in NSG Mice:**

MCF-7 cells and the stable ONECUT2-overexpressing cells (MCF-7/OC-2) were cultured in DMEM medium with 10% FBS. Cells were routinely passaged at 80-90% confluence and routinely screened for mycoplasma contamination.

**Chemotherapy Response Model**

2×10^6^ MCF-7 or MCF-7/OC-2 cells were injected into #4 mammary fat pad of six-week-old female NSG mice in a blinded and random manner. At ~200 mm³ tumor volume, mice were split into two groups: a pre-treatment cohort, which was sacrificed immediately (designated as the Pre-DTX group, n=4 per group), and a post-treatment cohort (designated as the Post-DTX group, n=4 per group), which received DTX (15 mg/kg) IP once a week for three weeks and sacrificed three days after the final chemodrug treatment. Tumor tissues were collected for subsequent experiments.

4. **BT474 Models in NSG Mice:**

BT474 cells and the stable ONECUT2-overexpressing cells (BT474/OC-2) were cultured in RPMI-1640 medium with 10% FBS. Cells were routinely passaged at 80-90% confluence and routinely screened for mycoplasma contamination.

**Chemotherapy Response Model**

2×10^6^ BT474 or BT474/OC-2 cells were injected into #4 mammary fat pad of six-week-old female NSG mice in a blinded and random manner. At ~200 mm³ tumor volume, mice were split into two groups: a pre-treatment cohort, which was sacrificed immediately (designated as the Pre-DTX group, n=4 per group), and a post-treatment cohort (designated as the Post-DTX group, n=4 per group), which received DTX (15 mg/kg) IP once a week for three weeks and sacrificed three days after the final chemodrug treatment. Tumor tissues were collected for subsequent experiments.
