# Supplementary material for: Low expression of ONECUT2 governs the POU6F2-beta-catenin axis to modulate cancer stemness and drives the CCL28-dependent pathway for macrophage polarization in breast cancer
Source: Cell Death Dis. 2026 Jun 2;17(1):690. doi: 10.1038/s41419-026-08909-5 (PMC13443588; doi:10.1038/s41419-026-08909-5)
Supplement: Supplementary file 3 [file 41419_2026_8909_MOESM3_ESM.docx]

**Table S2 Primer sequences and antibodies**

1. Primer sequences for RT-qPCR analysis

|  | **Forward primer ( 5′ to 3′)** | **Reverse primer ( 5′ to 3′)** |
| --- | --- | --- |
| CCL28 | GTCAGCCCGCACAACCATAC | CCTGTTACTGTTCCTCTTGCCATG |
| CCR3 | GGCAATGTGGTGGTGGTGATG | AATGGAAGGGTGACGAGGAAGAG |
| CCR10 | TTGGTCTCCGTCATCGTGTGG | CCTTCCCGCTGCCCATCC |
| ARG1 | GGACCTGCCCTTTGCTGACATC | TTCTTGACTTCTGCCACCTTGCC |
| IL-10 | GTTGTTAAAGGAGTCCTTGCTG | TTCACAGGGAAGAAATCGATGA |
| CD163 | ATCAACCCTGCATCTTTAGACA | CTTGTTGTCACATGTGATCCAG |
| CD206 | GACGTGGCTGTGGATAAATAAC | CAGAAGACGCATGTAAAGCTAC |
| β-Actin | ACAGAGCCTCGCCTTTGCCG | TTGCACATGCCGGAGCCGTT |
| TGFB1 | TACCTGAACCCGTGTTGCTCTC | GTTGCTGAGGTATCGCCAGGAA |

2. Primer sequences for ChIP-PCR analysis

| **Promoter** | **Region** | **Primers** | **Sequence** |
| --- | --- | --- | --- |
| POU6F2 | Site1 | Forward primer | TGTCCACTTCCAATTCACAAA |
|  |  | Reverse primer | TCTTAAGGGGTCCCTAATGATTT |
|  | Site2 | Forward primer | GCACAAAAATCAAAGTCTTAATGG |
|  |  | Reverse primer | TCTTCCGCTAAAGGTTTAACAA |
|  | Site3 | Forward primer | TTCGGTCCTTCATTCTTTGC |
|  |  | Reverse primer | GGTCGGGTAGCAATGAGAAA |
| CTNNB1 | Site1 | Forward primer | GCCCTGTAAAAGTGGTTAATCTG |
|  |  | Reverse primer | TGGTTGTGGTCACAATTCAAA |
|  | Site2 | Forward primer | GCCTAGAGAAACTGAATCGATCATA |
|  |  | Reverse primer | TCGATTAAGCAGCCTCCAAT |
|  | Site3 | Forward primer | CCAAAGAAAAATCCCCACAA |
|  |  | Reverse primer | CCGCAATTCAACAAGTCCTTA |
| CCL28 | Site1 | Forward primer | TGAAAACATAAAAAGAAATGGGTGAAA |
|  |  | Reverse primer | ACACTGGATTTCAAAGACTGGGT |
|  | Site2 | Forward primer | ACCTGGGAGTTCTGGTTTACAAAT |
|  |  | Reverse primer | CAGGGTCACTGTGAGGTGG |
|  | Site3 | Forward primer | GGAGGAGCCTGGTCTTAAGG |
|  |  | Reverse primer | CACAGAGAAAGTGCAGAGGA |

3. Primer sequences for dual-luciferase reporter assay

| CCL28 | Forward primer | GGTGCCAGAACATTTCTCTATCGAT |
| --- | --- | --- |
|  | Reverse primer | CAAGCTTACTTAGATCGCAGATCTC |
| POU6F2 | Forward primer | ACGACTCACTATAGGGAGACC |
|  | Reverse primer | GCCGCCACTGTGCTGGAT |
| CTNNB1 | Forward primer | CCAGAACATTTCTCTATCGATAGG |
|  | Reverse primer | GCTTTACCAACAGTACCGG |

4. siRNAs

| QIAGEN | Negative Control siRNA FlexiTube siRNA | SI03650325 |
| --- | --- | --- |
| QIAGEN | Hs_ONECUT2_7 FlexiTube siRNA | SI04340049 |
| QIAGEN | Hs_ONECUT2_8 FlexiTube siRNA | SI04346384 |
| QIAGEN | Hs_ONECUT2_6 FlexiTube siRNA | SI04181709 |
| QIAGEN | Hs_ONECUT2_4 FlexiTube siRNA | SI00665504 |
| QIAGEN | Hs_POU6F2_9 FlexiTube siRNA | SI05185635 |
| QIAGEN | Hs_POU6F2_6 FlexiTube siRNA | SI04200784 |
| QIAGEN | Hs_POU6F2_5 FlexiTube siRNA | SI03159499 |
| QIAGEN | Hs_POU6F2_1 FlexiTube siRNA | SI00690431 |
| QIAGEN | Hs_CTNNB1_5 FlexiTube siRNA | SI02662478 |
| QIAGEN | Hs_CTNNB1_9 FlexiTube siRNA | SI04379662 |
| QIAGEN | Hs_CTNNB1_8 FlexiTube siRNA | SI04379655 |
| QIAGEN | Hs_CTNNB1_4 FlexiTube siRNA | SI00029750 |

5. Sequences of overexpression plasmids

| CCL28 | Forward | ACTCACTATAGGGAGACCCAAGCTGGCTAGCGCCACCATGCAGCAGAGAGGACTCGCC |
| --- | --- | --- |
|  | Reverse | AGAATTCCACCACACTGGACTAGTGGATCCGAGCTCGGTACCAAGCTTCTACTTGTCATCGTC |
| POU6F2 | Forward | ACGACTCACTATAGGGAGACC |
|  | Reverse | GCCGCCACTGTGCTGGAT |

6. Antibodies for western blotting

| Proteintech | CCL28 Polyclonal antibody | 18214-1-AP | 1:1000 |
| --- | --- | --- | --- |
| ABclonal | CCR3 Rabbit pAb | A20253 | 1:1000 |
| Proteintech | CCR10 ployclonal antibody | 22071-1-AP | 1:1000 |
| Proteintech | ONECUT2 Polyclonal antibody | 21916-1-AP | 1:1000 |
| Invitrogen | Pou6f2 Polyclonal Antibody | PA5-148435 | 1:1000 |
| CellSignalingTechnology | β-Catenin Antibody | 9562S | 1:1000 |
| CellSignalingTechnology | Notch1 (D6F11) XP® Rabbit mAb | 4380S | 1:1000 |
| CellSignalingTechnology | Nanog Antibody | 3580S | 1:1000 |
| Proteintech | Lamin B1 Polyclonal antibody | 12987-1-AP | 1:1000 |
| CellSignalingTechnology | Phospho-GSK-3β (Ser9) (D85E12) XP® Rabbit mAb | 5558T | 1:1000 |
| CellSignalingTechnology | GSK-3β (D5C5Z) XP® Rabbit mAb #12456 | 12456T | 1:1000 |
| CellSignalingTechnology | GAPDH (14C10) Rabbit mAb | 2118S | 1:1000 |
| Proteintech | IL-10 Monoclonal antibody | 60269-1-Ig | 1:2000 |
| Proteintech | Arginase-1 Polyclonal antibody | 16001-1-AP | 1:5000 |
| Proteintech | TGF Beta 1 Recombinant monoclonal antibody | 81746-2-RR | 1:5000 |
| Abcam | Anti-N Cadherin antibody | ab76011 | 1:800 |
| Proteintech | E-cadherin Polyclonal antibody | 20874-1-AP | 1:5000 |
| Proteintech | Vimentin Polyclonal antibody | 10366-1-AP | 1:5000 |

7. Antibodies for IHC

| Proteintech | ONECUT2 Polyclonal antibody | 21916-1-AP | 1:600 |
| --- | --- | --- | --- |
| Invitrogen | Pou6f2 Polyclonal Antibody | PA5-148435 | 1:100 |
| CellSignalingTechnology | β-Catenin Antibody | 9562S | 1:1000 |
| Proteintech | CCL28 Polyclonal antibody | 18214-1-AP | 1:200 |

8. Antibodies for mIHC

| Proteintech | ONECUT2 Polyclonal antibody | 21916-1-AP | 1:200 |
| --- | --- | --- | --- |
| Invitrogen | Pou6f2 Polyclonal Antibody | PA5-148435 | 1:100 |
| CellSignalingTechnology | β-Catenin Antibody | 9562S | 1:100 |
| Sigma-Aldrich | Monoclonal anti-cytokeratin, PAN (mixture). | C2562 | 1:1000 |

9. Antibodies for flow cytometry

| Invitrogen™ | LIVE/DEAD™ Fixable Near IR (780) Viability Kit, for 633 nm excitation | L34994 |
| --- | --- | --- |
| biolegend | APC anti-mouse CD45 | 103112 |
| biolegend | PerCP/Cyanine5.5 anti-mouse CD3 | 100218 |
| biolegend | PE/Cyanine7 anti-mouse/human CD11b | 101215 |
| biolegend | Brilliant Violet 510™ anti-mouse CD4 | 100559 |
| biolegend | FITC anti-mouse F4/80 | 123107 |
| biolegend | PE/Cyanine7 anti-mouse CD8a | 100721 |
| biolegend | FITC anti-mouse CD19 | 152403 |
| biolegend | PE anti-mouse CD49b (pan-NK cells) | 108907 |
| biolegend | Brilliant Violet 421™ anti-mouse FOXP3 | 126419 |
| biolegend | PE anti-mouse CD163 | 155307 |
| biolegend | Zombie NlR Fixable Viability Kit | 423106 |
| biolegend | PerCP/Cyanine5.5 anti-human CD68 Antibody | 333814 |
| biolegend | APC anti-human CD163 Antibody | 326510 |
| BD Biosciences | PE Mouse Anti-Human CD24 | 555428 |
| biolegend | PerCP/Cyanine5.5 anti-mouse/human CD44 Antibody | 103032 |

10. Antibodies for Co-IP

| CellSignalingTechnology | Rabbit (DA1E) mAb IgG XP® Isotype Control | 3900S |
| --- | --- | --- |
| Proteintech | ONECUT2 Polyclonal antibody | 21916-1-AP |

11. Antibodies for ChIP-PCR

| Santa Cruz Biotechnology | POU6F2 antibody (F-7) | sc-376709 |
| --- | --- | --- |
| Proteintech | ONECUT2 Polyclonal antibody | 21916-1-AP |
